# A diagnostic model for Parkinson’s disease based on circadian rhythm-related genes

**DOI:** 10.1186/s12967-024-05424-z

**Published:** 2024-07-08

**Authors:** Lufeng Wang, Yiwen Bao, Xiaofan Duan, Hongxia Li, Hao Ding, Fei Yu, Jie yang, Yongbo Hu, Dongya Huang

**Affiliations:** 1 Department of Neurology, Shanghai East Hospital, School of Medicine, Tongji University, Shanghai, 200092 China; 2 Department of Oncology, Shanghai East Hospital, School of Medicine, Tongji University, Shanghai, 200092 China; 3 Department of Neurology, Shanghai Baoshan Luodian Hospital, Shanghai, 201908 China; 4 Department of Neurology, Changhai Hospital, Naval Medical University, Shanghai, 200433 China; 5 School Med, Tongji University, East Hospital, No. 150 Jimo Road, Shanghai, 200092 China

**Keywords:** Parkinson’s disease, Circadian rhythm, Bioinformatics, Nomogram

## Abstract

**Background:**

Circadian rhythm (CR) disturbance is intricately associated with Parkinson’s disease (PD). However, the involvement of CR-related mechanisms in the pathogenesis and progression of PD remains elusive.

**Methods:**

A total of 141 PD patients and 113 healthy participants completed CR-related clinical examinations in this study. To further investigate the CR-related mechanisms in PD, we obtained datasets (GSE7621, GSE20141, GSE20292) from the Gene Expression Omnibus database to identify differentially expressed genes between PD patients and healthy controls and further selected CR-related genes (CRRGs). Subsequently, the least absolute shrinkage and selection operator (LASSO) followed by logistic algorithms were employed to identify the hub genes and construct a diagnostic model. The predictive performance was evaluated by area under the curve (AUC), calibration curve, and decision curve analyses in the training set and external validation sets. Finally, RT‒qPCR and Western blotting were conducted to verify the expression of these hub genes in blood samples. In addition, Pearson correlation analysis was utilized to validate the association between expression of hub genes and circadian rhythm function.

**Results:**

Our clinical observational study revealed that even early-stage PD patients exhibited a higher likelihood of experiencing sleep disturbances, nocturnal hypertension, reverse-dipper blood pressure, and reduced heart rate variability compared to healthy controls. Furthermore, 4 CR-related hub genes (AGTR1, CALR, BRM14, and XPA) were identified and subsequently incorporated as candidate biomarkers to construct a diagnostic model. The model showed satisfactory diagnostic performance in the training set (AUC = 0.941), an external validation set GSE20295 (AUC = 0.842), and our clinical centre set (AUC = 0.805). Additionally, the up-regulation of CALR, BRM14 and the down-regulation of AGTR1, XPA were associated with circadian rhythm disruption.

**Conclusion:**

CR disturbance seems to occur in the early stage of PD. The diagnostic model based on CR-related genes demonstrated robust diagnostic efficacy, offering novel insights for future clinical diagnosis of PD and providing a foundation for further exploration into the role of CR-related mechanisms in the progression of PD.

**Supplementary Information:**

The online version contains supplementary material available at 10.1186/s12967-024-05424-z.

## Introduction

Parkinson’s disease (PD) is a prevalent neurodegenerative disorder characterized by the degeneration of dopaminergic neurons in the substantia nigra pars compacta (SNpc) [1]. PD patients often have typical movement disorders, such as bradykinesia, static tremor and postural instability [2]. However, before this range of motor symptoms occurs, PD patients often experience nonmotor symptoms such as sleep disturbances, hyposmia, and autonomic dysfunction [3]. Recent studies have revealed that the progression of nonmotor symptoms of PD is closely related to circadian rhythm (CR) disturbances [4–6].

The circadian rhythm, a 24-hour physiological and behavioural oscillation, is primarily regulated by the suprachiasmatic nucleus, exhibiting self-sustaining and synchronous electrical activity [7]. It is widely acknowledged that the sleep–wake cycle is intricately linked to circadian rhythm function [8]. In PD patients, impaired circadian rhythm may exacerbate disturbances in sleep-wake cycles through alterations in melatonin rhythmicity amplitude, leading to increased daytime sleepiness [9, 10]. A circadian clock control gene known as Tef has been identified to be associated with slow-wave sleep and movement disorders, including restless leg syndrome in in PD patients [11]. Additionally, the manifestation of CR disturbance in PD patients encompasses not only sleep-wake cycle disruption but also other dysfunctions within the autonomic nervous system, such as alterations in blood pressure and heart rate variability (HRV) [12–14]. Considering the close association between CR disturbance and early non-motor symptoms of PD, it is hypothesized that mechanisms related to CR disturbance may be activated during the early stages of PD. Therefore, investigating the mechanism underlying PD-related CR disturbance could unveil pathophysiological mechanisms associated with the initial phase of PD, which would hold significant implications for timely diagnosis and treatment initiation.

In this study, we conducted a comparative analysis of our clinical center to investigate CR disturbances in early-stage PD patients (Hoehn-Yahr stage I-II), suggesting potential involvement of CR-related mechanisms in the early progression of PD. To explore these mechanisms further, we employed bioinformatics and machine learning techniques to identify differentially expressed genes associated with circadian rhythms (DE-CRRGs), developed a diagnostic model for PD, and validated its effectiveness using an external validation dataset. Subsequently, we conducted an assessment of the expression levels of pertinent hub genes in participants’ blood samples to validate the significance of these identified hub genes as potential candidates, thereby further elucidating the crucial implications of CR-related genes in clinical diagnosis of PD.

## Materials and methods

### Participants

In this study, a total of 156 PD patients were recruited from the neurology department in our hospital, between September 2020 and October 2022. Among them, 12 patients did not cooperate with the completion of relevant examinations, and 3 refused to participate in the follow-up, resulting in a final inclusion of 141 PD patients. These patients were diagnosed with idiopathic PD according to the UK PD Society Brain Bank criteria. Additionally, we recruited a total of 113 matched healthy control participants from our hospital’s physical examination centre. All participants provided informed consent for their participation in this study which was approved by our hospital’s Ethics Committee.

### Clinical data collection and evaluation

Participants’ sleep-related clinical indicators were recorded using polysomnography (PSG). In this study, sleep fragmentation was defined as the occurrence of frequent microarousals lasting more than 3 s but less than 10 s [15]. Sleep-disordered breathing (SDB) was defined as the presence of five or more apnoeic or hypopnoeic events per hour [15]. The Disease Sleep Scale (PDSS), a standardized Chinese version consisting of 15 specific items, was used to assess the total sleep quality of PD patients [9]. Blood pressure (BP)-related indicators were monitored by measuring 24 h ambulatory blood pressures. In this study, (1) nocturnal hypertension was considered a mean nighttime blood pressure ≥ 120/70 mmHg; (2) awakening hypotension was considered a systolic blood pressure that had decreased by ≥ 20 mmHg within 60 min after getting up in the morning compared with the average systolic blood pressure (SBP) measured the last three times before getting up; (3) reverse dipping hypertension was considered a decrease in BP at night of less than 10% [the calculation formula is as follows: the decrease in BP at night = (average SBP during the day-average SBP at night)/average SBP during the day *100%]; and (4) weighted blood pressure variability (wBPV) was used as the evaluation index of blood pressure variability. The calculation formula was as follows: sum of standard deviations of daytime and nighttime systolic blood pressure after time correction [(daytime blood pressure variability*15 + nighttime blood pressure variability) *9/24]. HRV-related indicators were obtained by conducting 24-hour Holter electrocardiograms, while participants were instructed to engage in their routine daily activities, excluding any strenuous physical exertion. The root mean square of successive RR interval differences (RMSS) and the standard deviation of all NN intervals (SDNN) were measured as parameters for assessing HRV [13]. Cognitive assessments were performed by two neurologists who remained blinded to the groupings.

### Identification and analysis of DE-CRRGs

The Gene Expression Omnibus (GEO) database (http://www.ncbi.nlm.nih.gov/geo/) was utilized to obtain three expression profiles, namely, GSE7621, GSE20141, GSE20292. Following batch effect removal using the “sva” software package, these datasets were merged to create a total of 72 samples consisting of 37 PD patients and 35 healthy controls. The differentially expressed genes (DEGs) were identified by the thresholds of log fold change > 0 and *p* < 0.05 after correction. In addition, weighted gene coexpression network analysis (WGCNA) was used to identify the PD-related DEGs. The “goodSamplesGenes” function from the WGCNA package was utilized to filter out genes and samples that did not meet the quality criteria. The dynamic tree cutting method was employed to identify modules exhibiting the strongest correlation with clinical features. Additionally, CR-related genes (CRRGs) were obtained from the GeneCards database (https://www.genecards.org/), excluding those with correlation scores lower than 0.4 (as shown in Table S1). Finally, DE-CRRGs were determined as the intersection between DEGs, WGCNA and CRRGs.

The distribution pattern of genes was analysed by gene set enrichment analysis (GSEA). By employing GSEA, distinct differences in pathway enrichment between PD patients and healthy controls were identified. Gene Ontology (GO) functional analysis was employed to annotate the gene characteristics. To gain insights into the pathways, Kyoto Encyclopedia of Genes and Genomes (KEGG) analysis was conducted on these DE-CRRGs. The disease enrichment analysis was conducted by DisGeNET (https://www.disgenet.org/). The protein‒protein interaction (PPI) network was established by utilizing the STRING database.

### Identification of hub genes from DE-CRRGs

In order to further select the most disease-relevant characteristic variables from DE-CRRGs, we opted to employ regularization techniques for variable reduction in order to mitigate issues of overfitting and multicollinearity. LASSO, a commonly utilized regularization technique, simplifies the model by shrinking coefficients of less correlated variables towards zero. The resultant relevant characteristic variables selected through this approach were subsequently fine-tuned using 10-fold cross-validation [16]. Logistic regression allows for interpretable coefficients that demonstrate the correlation between each characteristic variable and the outcome [17]; hence, we utilized logistic regression to evaluate the impact of LASSO’s selected variables on outcomes, identifying hub genes with *p* < 0.05 as having significant correlations with disease, which were subsequently used for constructing predictive models.

### Construction and validation of the model

A nomogram was constructed by multiplying the expression level of each hub gene (expi) with the risk coefficient (coefi) to obtain a risk score, which was then combined with the patient’s clinical information (age, sex). The effectiveness of the nomogram was verified using the “pROC” package to calculate the area under the receiver operating characteristic curve (AUC). Calibration curves were constructed by the “Hmisc” package to assess consistency between predicted probability and actual probability. Furthermore, a decision curve analysis (DCA) was performed by the “rmda” package to show the net benefit of intervention based on the diagnostic model for PD diagnosis (net benefit was defined as the treatment benefit for PD patients diagnosed by the model minus the harm for non-PD patients diagnosed by the model). Additionally, external independent datasets (GSE20295) and blood samples from our clinical centre were used to validate the robustness and reliability of this model.

### Single-gene gene set variation analysis (GSVA) enrichment

The GSVA method is widely recognized for its heightened sensitivity in detecting subtle variations in pathway activation levels within a sample population [18]. We compared the differences in GSVA scores between high- and low-expression samples of hub genes to observe differences in pathway enrichment. In the group exhibiting high expression of target genes, a positive t value (> 0) signified the activation of associated pathways. Conversely, in the group displaying low expression of the target gene, a negative t value (< 0) indicated activation of the relevant pathway.

### Prediction of hub gene-related interactive genes, ceRNA networks and targeted drugs

The GeneMANIA database was employed to predict the gene interactions. A combination of MIRDB, TargetScan, and miRanda databases was used to construct the ceRNA network. The DSigDB database was utilized to identify potential target drugs, aiding in the search for potential therapeutic targets related to PD.

### Immunoinfiltration analysis

The “CIBERSORT” package was utilized for quantitative analysis to compare and contrast the relative proportions of diverse invasive immune cells across different samples. Subsequently, correlation analysis was conducted to further investigate the associations between these immune cells as well as between these hub genes and the aforementioned immune cells.

### Real-time quantitative PCR (RT-qPCR)

For mRNA detection, total RNA was extracted and transcribed into cDNA according to the manufacturer’s protocol (Invitrogen, CA, USA). The mRNA levels of the hub genes were analysed using the 2^−ΔΔCt^ method. The sequences of the primers used are provided in Table S2.

### Western blotting

We collected blood samples from 74 patients in the PD group and 62 patients in the healthy control group. These blood samples were centrifuged at a speed of 3000 rpm for 10 min to obtain serum, which was then frozen at − 80 °C for subsequent analysis. The expression of these proteins was verified by Western blotting. The following primary antibodies were used for Western blotting: anti-AGTR1 (1:1000, Proteintech, 25343-1-AP), anti-CALR (1:1000, Abcam, ab92516) 10292-1-AP), anti-RBM14 (1:1000, Proteintech, 10196-1-AP) and anti-XPA (1:1000, Proteintech, 16462-1-AP). Finally, ImageJ software was used to quantitatively analyse the relative density of the bands.

### Data analysis

In this study, the unpaired T test and Mann‒Whitney U test were used to compare means and medians between groups, respectively. The chi-square test was employed to compare classified data across groups. Statistical analysis was conducted using SPSS 26 software and R software (version 4.3.1). Statistical significance was determined at a two-tailed *p* < 0.05.

## Results

### Circadian rhythm disturbance occurs in patients with early-stage PD

As shown in Table 1, there were no significant differences in baseline clinical characteristics between the two groups. After adjusting for covariates including age, sex, and dopamine equivalent dose (LED), both early-stage (Hoehn–Yahr stages I-II) and late-stage (Hoehn–Yahr stages III-V) PD patients with sleep disorders exhibited notable reductions in total sleep score (PDSS) compared to healthy controls (*P* < 0.05) (Table 2). In addition, PD patients exhibited a significant increase in sleep fragmentation (*P* < 0.05) compared to healthy controls, accompanied by an elevated ratio of periodic limb movement (PLM) (*P* < 0.05). However, no significant difference was observed in SDB between early-stage PD patients and healthy controls, whereas late-stage PD patients had a higher likelihood of experiencing SDB than healthy controls (*P* = 0.047). Through the utilization of ambulatory blood pressure monitoring over a period of 24 h, it was found that both early- and late-stage PD patients were more susceptible to nocturnal hypertension and reverse dipping hypertension blood pressure patterns than healthy controls (*P* < 0.05). Awakening hypotension also served as an indicator of autonomic nervous system disturbance; however, it was found that morning hypotension occurred more frequently only among late-stage PD patients (*P* = 0.037), while no significant difference was observed between early-stage PD patients and healthy controls. Moreover, there was no significant disparity observed in the wBPV between the PD group and the healthy controls. Additionally, 24-hour Holter electrocardiogram monitoring revealed significantly diminished nocturnal SDNN in both early- and late-stage PD patients compared to healthy controls (*P* < 0.05), indicating partial impairment of autonomic nervous function in PD patients. With regard to cognitive assessment, no notable distinction was found between patients with early PD and healthy controls; however, patients with late-stage PD exhibited varying degrees of cognitive impairment compared to healthy controls (*P* < 0.001).

**Table 1:** Clinical and demographic information about PD patients and non-PD controls

Variables	PD patients					Non-PD controls	P*
	Hoehn-Yahr Stage				All		
	I	II	III	IV/V			
Participants, n	35	32	41	33	141	113	
Age, years	69.3 ± 7.1	69.1 ± 8.6	70.5 ± 6.6	72.4 ± 7.4	70.3 ± 7.3	69.9 ± 9.2	0.57
Gender, male, n (%)	18/35	14/32	25/41	19/33	76(53.9)	57(50.4)	0.61
Education, years	6.5 ± 1.6	6.7 ± 1.4	6.4 ± 1.7	6.4 ± 1.8	6.4 ± 1.7	6.5 ± 1.8	0.65
Smoking, n (%)	2	4	3	3	12(8.5)	8(7.1)	0.81
Drinking, n (%)	5	5	6	3	19(13.5)	12(10.6)	0.57
Hypertension, n (%)	20	19	25	22	86(61.0)	66(58.4)	0.7
Diabetes mellitus, n (%)	4	4	5	5	18(12.8)	12(10.6)	0.69
BMI, kg/m^2^	24.6 ± 3.1	24.5 ± 3.9	24.6 ± 3.5	24.4 ± 4.0	24.5 ± 3.8	24.6 ± 4.1	0.75
PD duration, median, month	29 (12, 48)	44 (22, 69)	71(39, 114)	114(65, 176)	55(31, 108)		
LED, mg	176.3 ± 146.8	416.3 ± 273.1	594.5 ± 316.7	749.2 ± 514.2	486.5 ± 296.7		

^a^ P value was obtained by comparing all PD patients and non-PD controls

Abbreviations: PD, Parkinson’s disease; BMI, Body Mass Index; LED, levodopa equivalent dose

**Table 2:** Circadian rhythm related characteristics between Non-PD controls and PD patients

Variables	Non-PD controls	PD patients			
		Hoehn-Yahr Stage	*P* ^a^	Hoehn-Yahr Stage	*P* ^b^
		Early (I and II)		Late (III, IV, and V)	
Participants, n	113	67		74	
**Sleep disturbances**					
Sleep fragmentation, n (%)	38(33.6)	33(46.5)	0.041^*^	41(55.4)	0.004^**^
SDB, n (%)	39(34.5)	24(35.8)	0.873	36(48.6)	0.047^*^
PLM, n (%)	5(4.4)	9(13.4)	0.042^*^	13(17.6)	0.004^**^
PDSS	126.5 ± 7.9	111.6 ± 12.6	< 0.001^***^	85.4 ± 15.7	< 0.001^***^
**Blood pressure**					
Nocturnal hypertension, n (%)	29(25.7)	30(44.8)	0.013^*^	31(41.9)	0.025^*^
Awakening hypotension	12(10.6)	12(17.9)	0.179	17(23.0)	0.037^*^
Reverse-dipper BP, n (%)	22(19.5)	23(34.3)	0.033^*^	33(44.6)	< 0.001^***^
WBPV	14.29 ± 6.93	15.39 ± 6.32	0.286	16.24 ± 6.67	0.067
**Heart rate variability**					
RMSS D, ms	23.5 ± 7.7	22.4 ± 8.3	0.372	21.6 ± 7.9	0.124
RMSS N, ms	31.7 ± 10.3	30.5 ± 11.1	0.435	29.3 ± 11.7	0.186
SDNN D, ms	99.2 ± 34.6	98.6 ± 36.2	0.832	94.7 ± 41.6	0.186
SDNN N, ms	113.9 ± 37.1	103.2 ± 38.7	0.048^*^	99.5 ± 42.9	0.031^*^
**Cognitive function**					
MMSE	27.6 ± 1.3	27.2 ± 1.5	0.117	26.5 ± 1.8	< 0.001^***^
MoCA	26.8 ± 2.1	26.3 ± 1.9	0.083	24.4 ± 2.6	< 0.001^***^

^a^ P value was obtained by comparing early-stage PD patients and non-PD controls, ^b^ P value was obtained by comparing late-stage PD patients and non-PD controls

**Table 3:** Information for microarray datasets from GEO database

GSE accession	Samples		Platform	Country	Contributor	Attribute
	PD (*n*)	Control (*n*)				
GSE7621	16	9	GPL570	USA	Ffrench-Mullen JM	Training set
GSE20141	10	8	GPL570	USA	Middleton FA et al.	Training set
GSE20292	11	18	GPL96	USA	Middleton FA et al.	Training set
GSE20295	40	53	GPL96	USA	Zhang Y et al.	Validation set

### Identification and analysis of PD-related DE-CRRGs

**Fig. 1:**
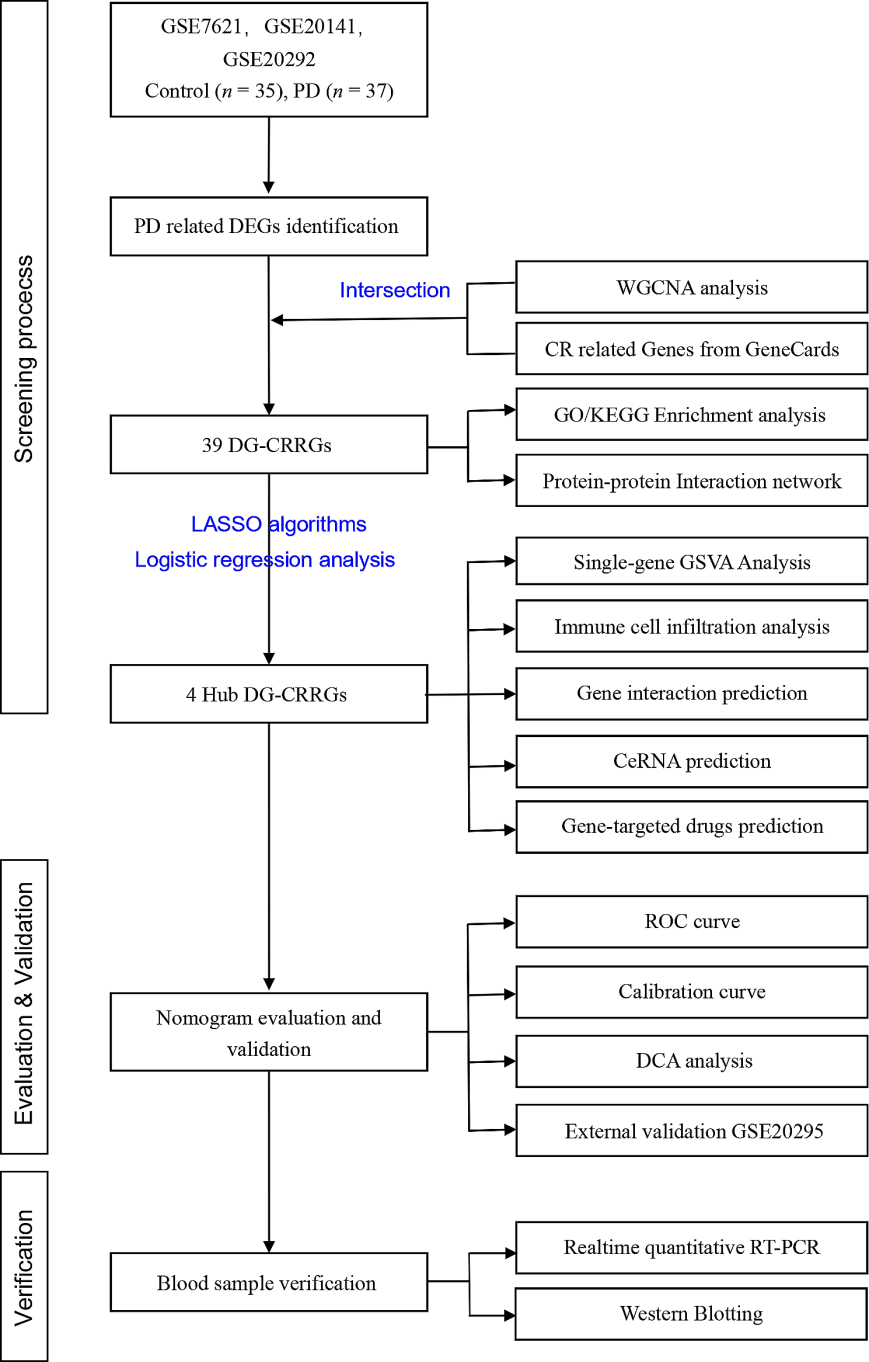
Flowchart of the screening and analysis strategy

**Fig. 2:**
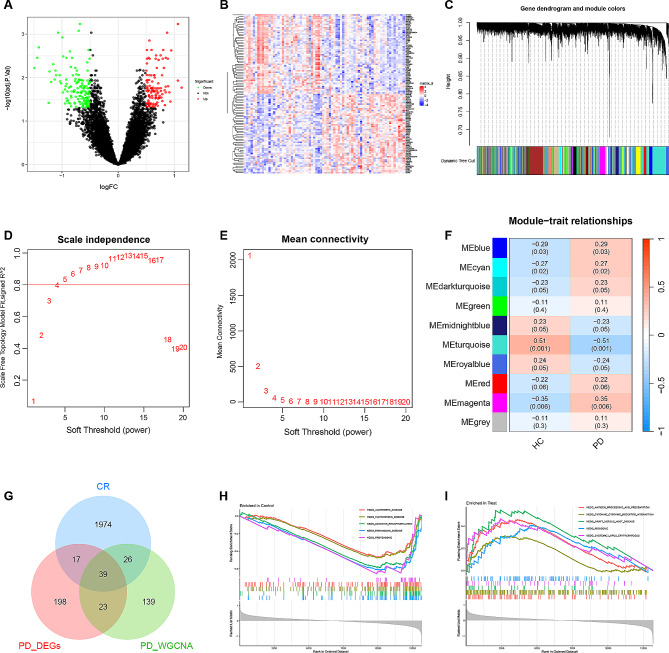
**Screening process of DE-CRRGs and GSEA.** (**A**) Volcano graph displaying the DEGs associated with Parkinson’s disease (PD). (**B**) Heatmap of the DEGs. (**C**) Dendrogram of the PD gene cluster. (**D**, **E**) Scaleless index and mean connectivity of soft thresholds. (**F**) Heatmap of correlations between modules and clinical traits. (**G**) Venn diagram showing the intersecting genes as DE-CRRGs. (**H**, **I**) GSEA of DEG sets in the PD and control groups

**Fig. 3:**
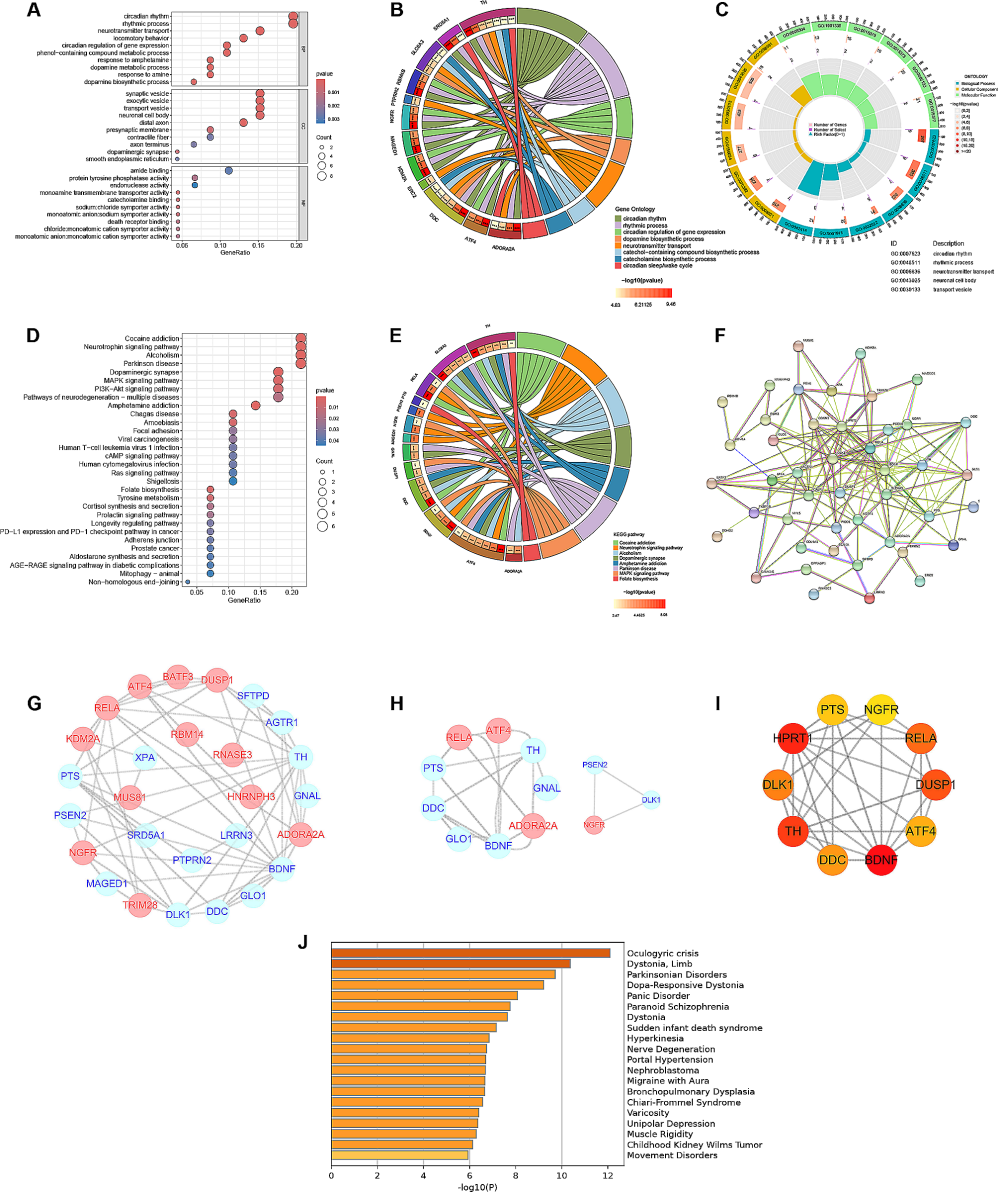
**Analysis of DE-CRRGs.** (**A**) Bubble plot of the Gene Ontology (GO) enrichment analysis. (**B**) Circos plot presenting the correlations between the top 8 GO functions and DE-CRRGs. (**C**) Circos plot presenting the GO functions (largest circle), the number of genes involved in each pathway (middle circle), and the percentage of DEGs in each function (inner circle). (**D**) Bubble plot of KEGG signalling pathways. (**E**) Circos plot presenting the correlations between the top 8 pathways and DE-CRRGs. (**F**) PPI network of CR-related DEGs. (**G**) PPI network of upregulated and downregulated proteins. Red dots represent upregulated proteins, and blue dots represent downregulated proteins. (**H**) Key clusters calculated by MCODE. (**I**) Top 10 genes chosen by cytoHubba. (**J**) Disease enrichment analysis of DE-CRRGs

The flowchart is shown in Fig. 1. A total of 277 PD-related DEGs were screened from three datasets, namely, (GSE7621, GSE20141, GSE20292), comprising 72 samples (Fig. 2A, B). With a soft threshold β set at 4, the WGCNA method successfully identified the module genes of utmost importance. Through hierarchical clustering and dynamic tree function analysis, it was observed that the turquoise module exhibited a robust negative correlation with PD (*r* = − 0.51, *p* = 0.001), while the magenta module displayed a significant positive correlation with PD (*r* = 0.35, *p* = 0.006) (Fig. 2C-F). By intersecting with 2056 CR-related genes from GeneCards, 39 DE-CRRGs were further identified (Fig. 2G). The GSEA indicated that the different pathways in the two sample groups were primarily enriched in PD, Alzheimer’s disease, and Huntington’s disease (Fig. 2H, I). Subsequently, enrichment analyses were performed separately on these DE-CRRGs (Fig. 3A-C). The results of GO analysis revealed significant enrichment of DE-CRRGs in the Biological Process module associated with circadian rhythm and neurotransmitter transport. In terms of cellular component module analysis, these genes exhibited enrichment in neuronal cell bodies and synaptic vesicles (Fig. 3A-C). Furthermore, KEGG pathway enrichment analysis demonstrated that these genes predominantly participated in PD progression as well as neurotrophic factor signalling pathways and dopamine synapses, among others (Fig. 3D, E). These findings suggest that not only are these DE-CRRGs involved in the progression of PD, but they may also be closely associated with circadian rhythm function. Additionally, protein interaction relationships between DE-CRRGs were visualized using Cytoscape to present subnetworks exhibiting high correlation (Fig. 3F-I). The disease enrichment analysis also revealed that these DE-CRRGs were tightly associated with Parkinsonian disorders, DOPA-responsive distonia and nerve degeneration (Fig. 3J).

### Construction and validation of a diagnostic model

**Fig. 4:**
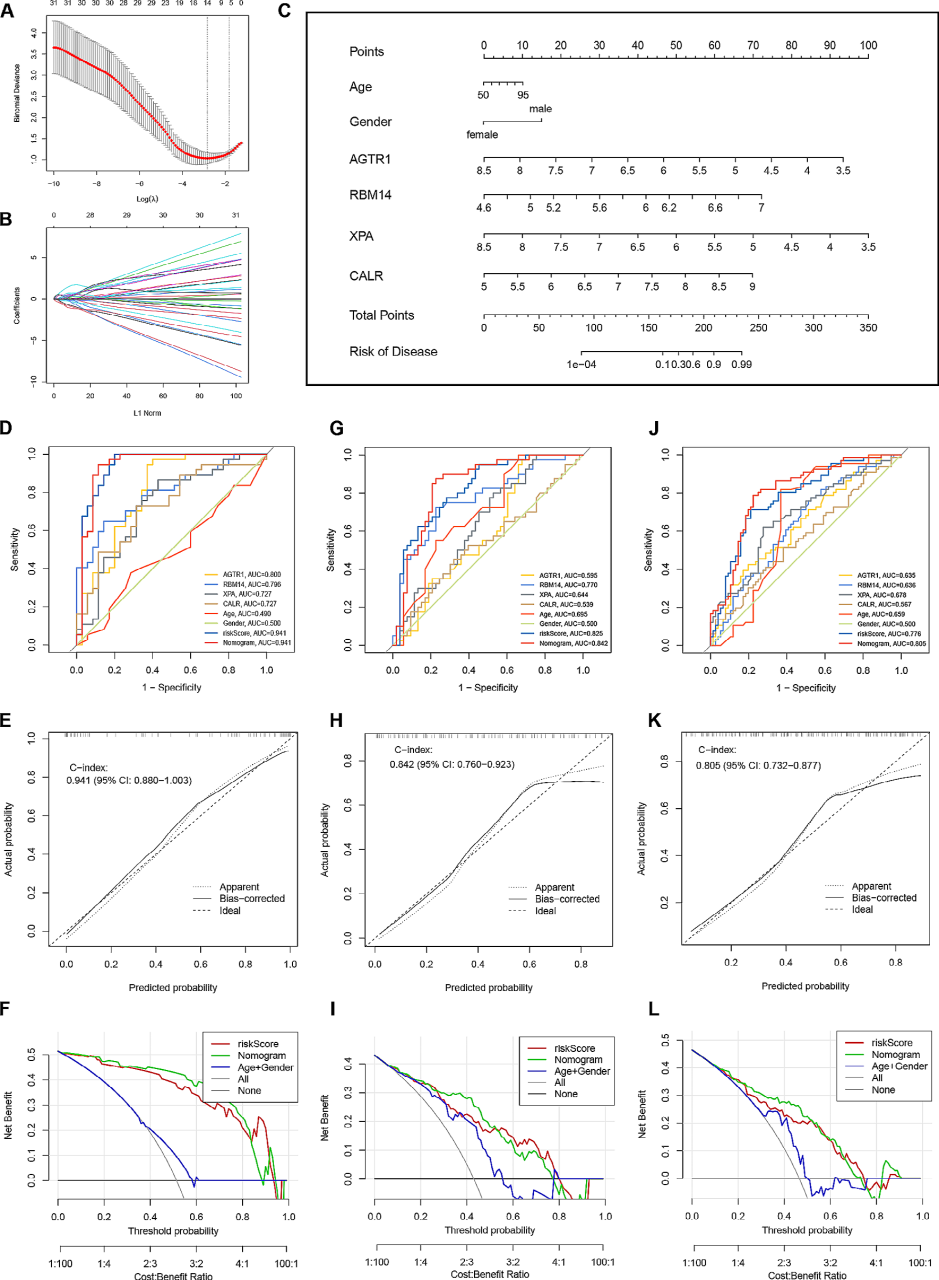
**Identification of hub genes and construction of the nomogram.** (**A**, **B**) LASSO algorithm. Fourteen characteristic genes were screened out by the LASSO algorithm. (**C**) Nomogram incorporating age, sex, and four hub genes (AGTR1, RBM14, XPA, CALR). (**D**, **G**, **J**) Receiver operating characteristic (ROC) curves of the nomogram in the training datasets (GSE7621, GSE20141, GSE20292), the validation dataset (GSE20295) and our external clinical dataset. (**E**, **H**, **K**) Calibration plots of the nomogram in the training datasets (GSE7621, GSE20141, GSE20292), the validation dataset (GSE20295) and our external clinical dataset. (**F**, **I**, **L**) Decision curve analysis (DCA) curves of the nomogram in the training datasets (GSE7621, GSE20141, GSE20292), the validation dataset (GSE20295) and our external clinical dataset

We employed the machine learning LASSO algorithm in conjunction with logistic regression to further identify the hub genes most strongly associated with PD. By applying a penalty coefficient, LASSO selected 14 characteristic variables, which were subsequently subjected to logistic regression analysis to eliminate variables with *P* < 0.05 (Fig. 4A, B). Subsequently, the logistic regression algorithm further identified four hub genes, namely, RNA binding motif protein 14 (RBM14), calreticulin (CALR), angiotensin II receptor type 1 (AGTR1), and xeroderma pigmentosum group A-complementing protein (XPA), with a significance threshold of *p* < 0.05. A diagnostic model was constructed using a nomogram model in the training dataset based on these four hub genes (Fig. 4C). The nomogram allows for projecting each indicator’s amount onto the top scale to obtain its corresponding score. The scores obtained from each index were then added together and projected onto the bottom scale to determine the probability of PD in the subject. Through ROC analysis, we found that each gene had an AUC greater than 0.7 in the training set, resulting in an overall AUC of 0.941 for the nomogram in the training set (Fig. 4D). Furthermore, the external independent validation dataset (GSE20295) also showed an overall AUC of 0.842, indicating the satisfactory applicability and reliability of this nomogram (Fig. 4G, J). Moreover, calibration curves demonstrated high consistency between actual measurements and predictions (Fig. 4E, H). DCA also revealed satisfactory clinical benefits associated with this nomogram in training and external validation datasets (Fig. 4F, I). Finally, the model also demonstrated a satisfactory applicability (AUC = 0.805), robust consistency, and acceptable clinically net benefit in our clinical blood sample validation dataset obtained from our clinical centre (Fig. J, K, L).

**Fig. 5:**
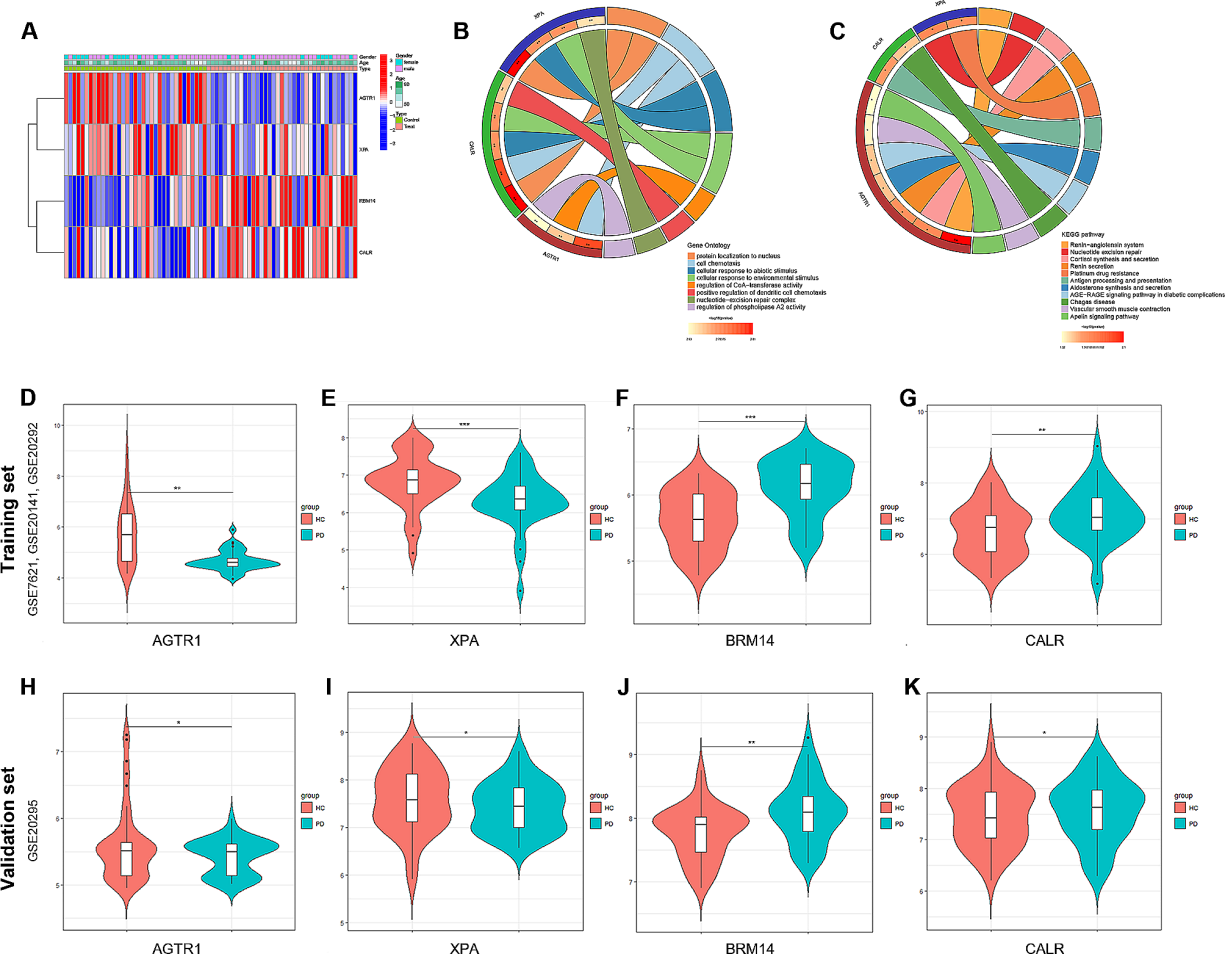
**Enrichment analysis of four hub genes.** (**A**) Heatmap of the four hub genes. (**B**) Circos plot presenting the correlations between the hub genes and GO functions. (**C**) Circos plot presenting the correlations between the hub genes and KEGG pathways. (**D**-**G**) Violin plots presenting the differential expression of hub genes in the training set. (**H**-**K**) Violin plots presenting the differential expression of hub genes in the external validation set

The cluster heatmap shown in Fig. 5A visually represents the differential expression patterns of these hub genes between the PD and control groups. The GO enrichment analyses revealed significant associations of these hub genes with CoA-transferase activity and the regulation of dendritic cell chemotaxis (Fig. 5B). The KEGG analysis indicated a significant enrichment of these genes in the renin-angiotensin system pathway (Fig. 5C). The expression of hub genes was represented by violin plots in the training set and the external validation set GSE20295 (Fig. 5D-K).

### Hub genes associated with PD-related pathways

**Fig. 6:**
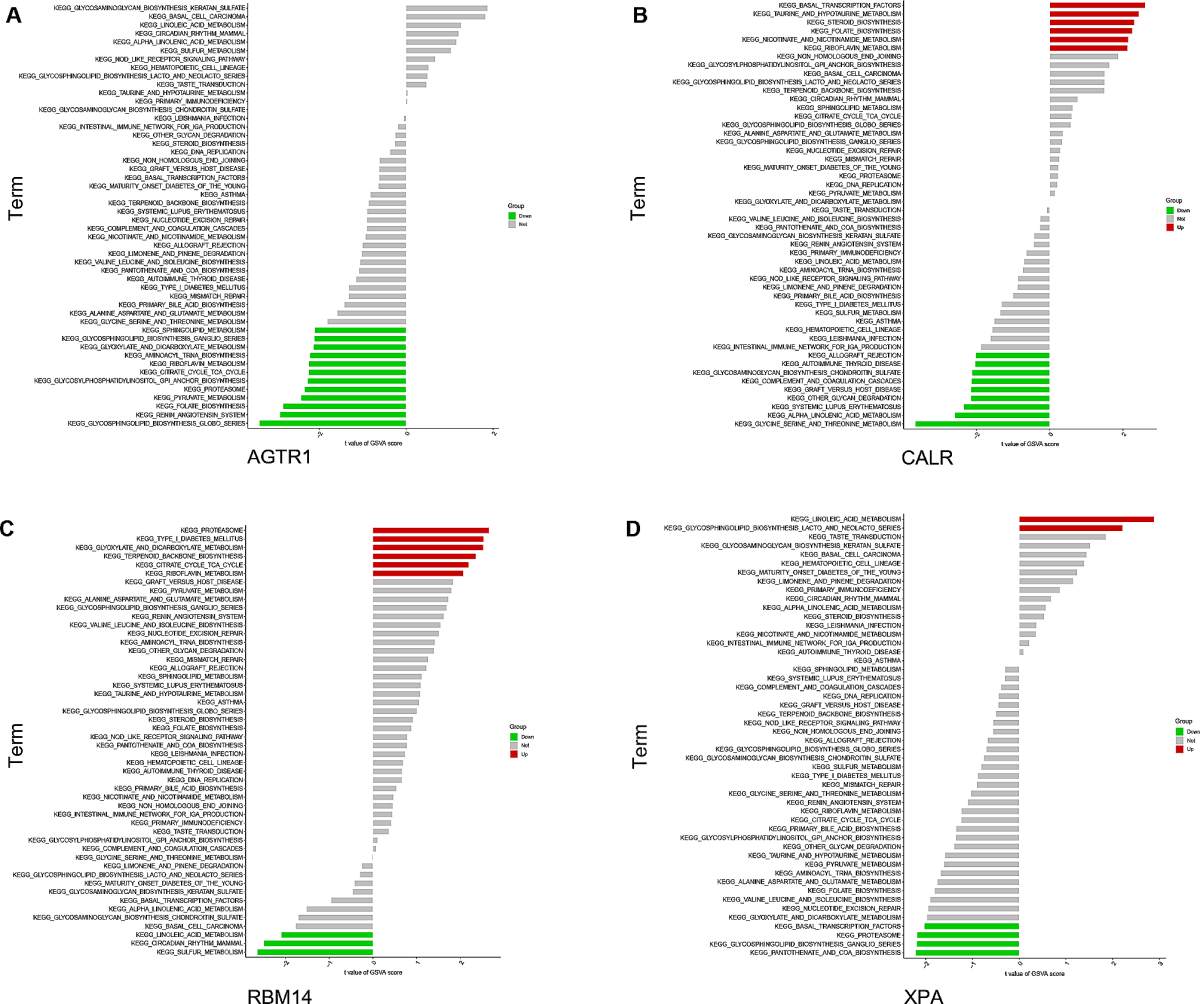
**Gene set variation analysis (GSVA) of each hub gene.** Red signalling pathways are enriched with high expression of the hub genes, while the green signalling pathways are enriched with low expression of the hub genes. (**A**) AGTR1. (**B**) XPA. (**C**) RBM14. (**D**) CALR

Single-gene GSVA pathway enrichment analysis was performed to reveal the activated pathways between the groups with high and low expression of each hub gene (Fig. 6). The high expression of AGTR1 was found to be closely associated with active pathways, namely, glycosphingolipid biosynthesis and proteasome activity. Similar to AGTR1, it is also highly correlated with the proteasome in the high expression of RBM14. The low expression of RBM14 was associated with circadian rhythm mammals and sulfur metabolism. The high expression of CALR was related to taurine and hypo taurine metabolism and steroid biosynthesis, while the low expression of CALR was related to glycine, serine and threonine metabolism. In addition, the high expression of XPA may activate the linoleic acid metabolism pathway, while the low expression of XPA was closely related to proteasome, pantothenate and CoA biosynthesis.

### Relationships between hub genes and immune cells

**Fig. 7:**
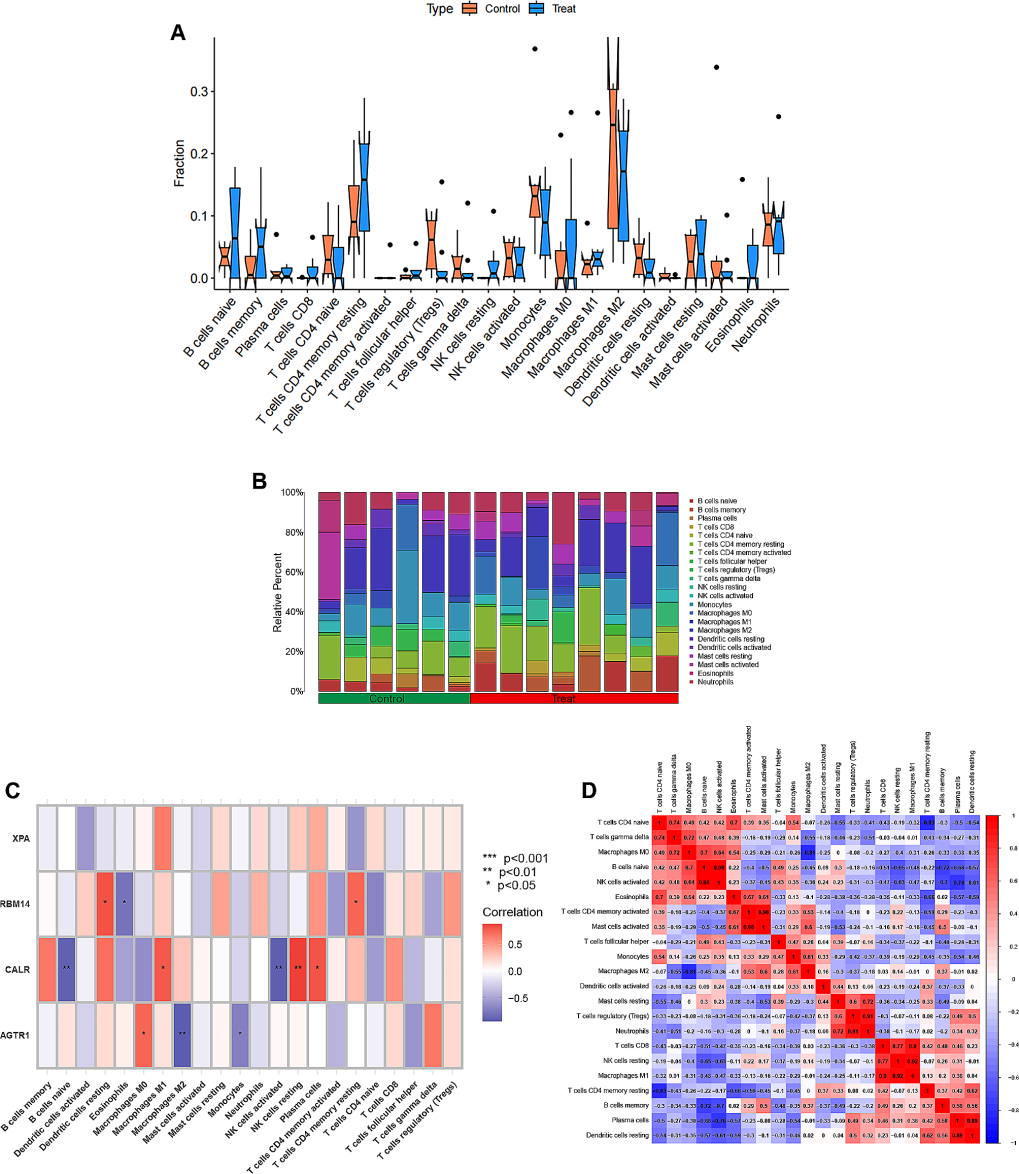
**Immunoinfiltration analysis.** (**A**) Discrepancy in the 22 types of immune cell infiltration between the control and PD groups. (**B**) Stacked histogram presenting the proportions of immune cells among the control and PD groups. (**C**) Correlation analysis of each hub genes and the different types of immune cells. (**D**) Correlation analysis among the different types of immune cells. *p < 0.05, **p < 0.01, *** *p* < 0.001

**Fig. 8:**
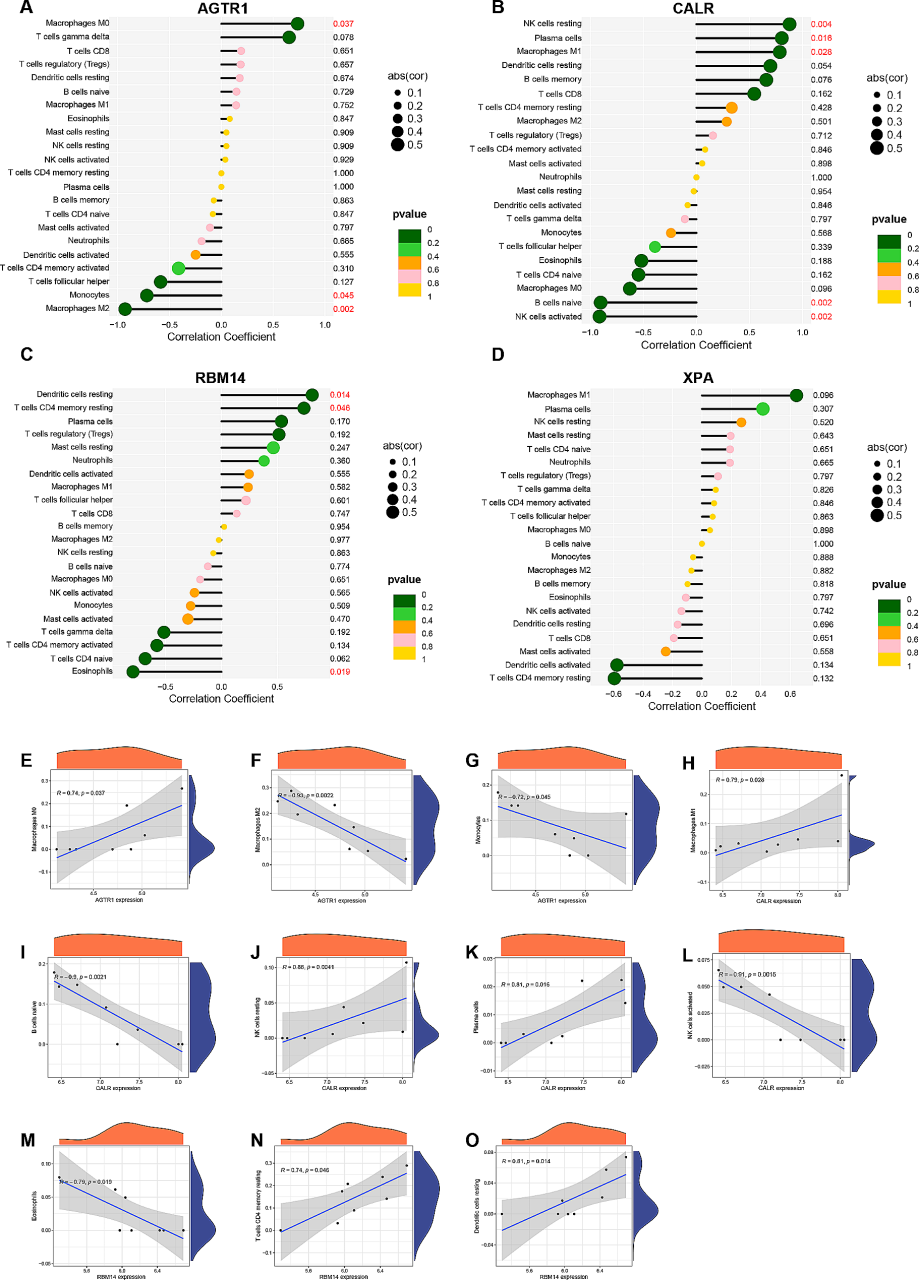
**Correlation analysis of hub genes and immune cells.** (**A**-**D**) Lollipop graphs presenting the correlations between each hub gene and immune cells. (**E**-**F**) Scatter plots presenting significant correlations between each hub gene and immune cells

The CIBERSORT algorithm is commonly employed for the analysis and comparison of immune cell infiltration in PD and healthy control groups [19]. We observed disparities in the infiltrating components of immune cells between these two groups (Fig. 7A, B). Compared to the control group, the PD group had higher proportions of infiltrating plasma cells, CD8 T cells, T follicular helper cells, M0 macrophages, and eosinophils. Conversely, there were decreases in the proportions of infiltrating Tregs, T gamma delta cells, activated mast cells and neutrophils when compared with the control group. Subsequently, Pearson correlation analysis was performed to investigate the correlations between hub genes and immune cell types (Fig. 7C). Correlation analyses among various immune cell types were also performed (Fig. 7D). As shown in the lollipop diagrams (Fig. 8A-D), AGTR1 demonstrated close relationships with M0 macrophages (*p* = 0.037), monocytes (*p* = 0.045) and M2 macrophages (*p* = 0.002). CALR showed strong positive correlations with plasma cells (*p =* 0.016), naive B cells (*p* = 0.002), and active and resting NK cells (*p* < 0.01). RBM14 exhibited significant positive correlations with dendritic cells (*p* = 0.014) and eosinophils (*p* = 0.019). However, no significant associations were observed between XPA and any immune cell type in the correlation analysis. Scatter plots (Fig. 8E-L) were then employed to visually depict the significant correlations between each hub gene and immune cells.

### Prediction of hub gene-related interacting genes, ceRNA networks and targeted drugs

**Fig. 9:**
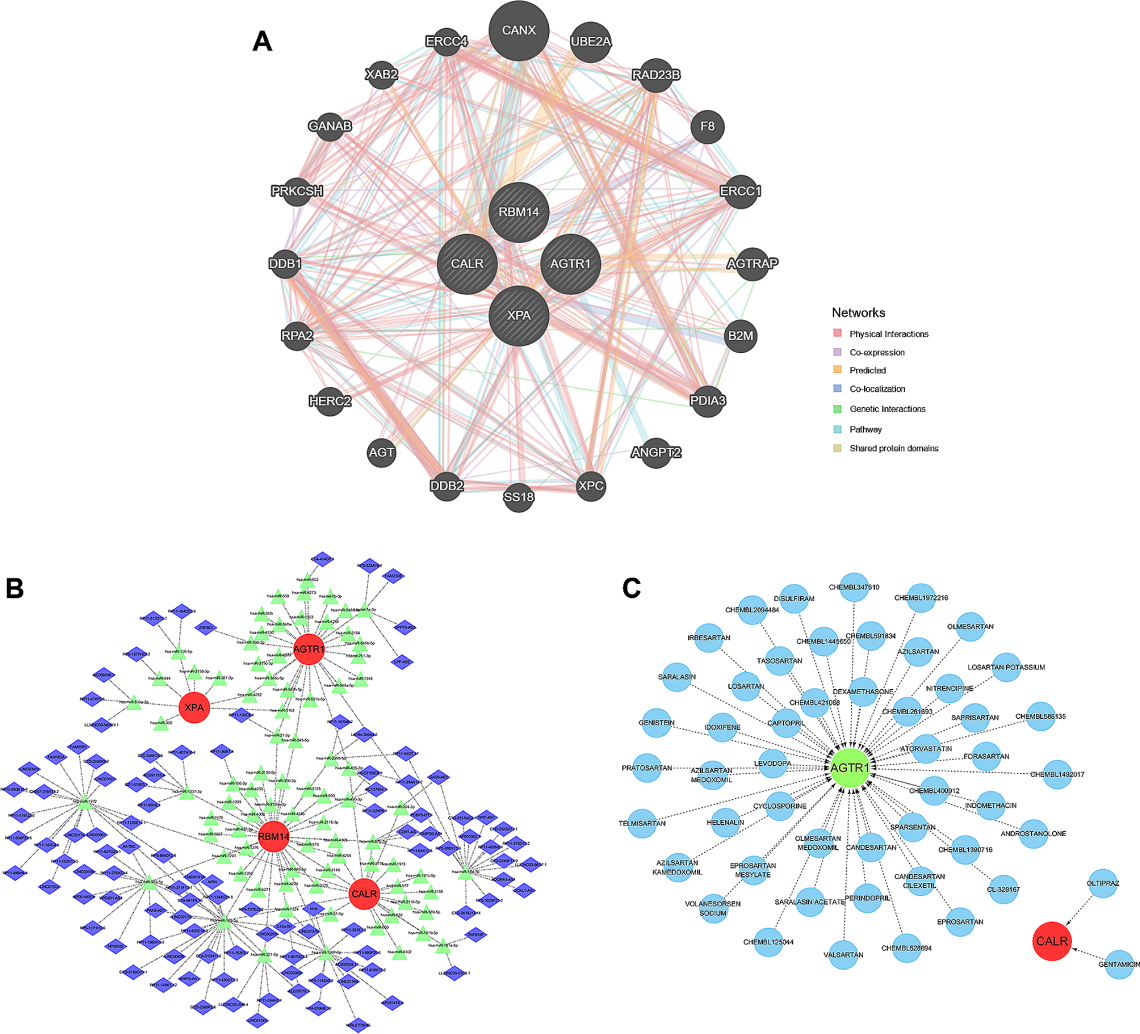
**Prediction of interactive genes, ceRNA networks, and target drugs.** (**A**) Prediction of hub gene-related interactive genes. (**B**) Prediction of hub gene-related ceRNA networks. (**C**) Prediction of hub gene-targeted drugs

To explore other genes that had potential interactions with the 4 hub genes, we obtained 20 potential genes that interacted with the hub genes from GeneMANIA (Fig. 9A). In addition, we used the MIRDB, TargetScan and miRanda databases to jointly screen potential miRNAs interacting with the hub genes and then constructed the ceRNA network using the SpongeScan database (Fig. 9B). Upon screening for potential drugs targeting the hub genes, we found 43 potential drugs targeting AGTR1 and 2 potential drugs targeting CALR through the DGIdb database (Fig. 9C). Unfortunately, we failed to identify targeted drugs for the remaining two hub genes, RBM14 and XPA, in the database.

### Verification of hub gene expression and association with sleep disorders in PD patients

**Fig. 10:**
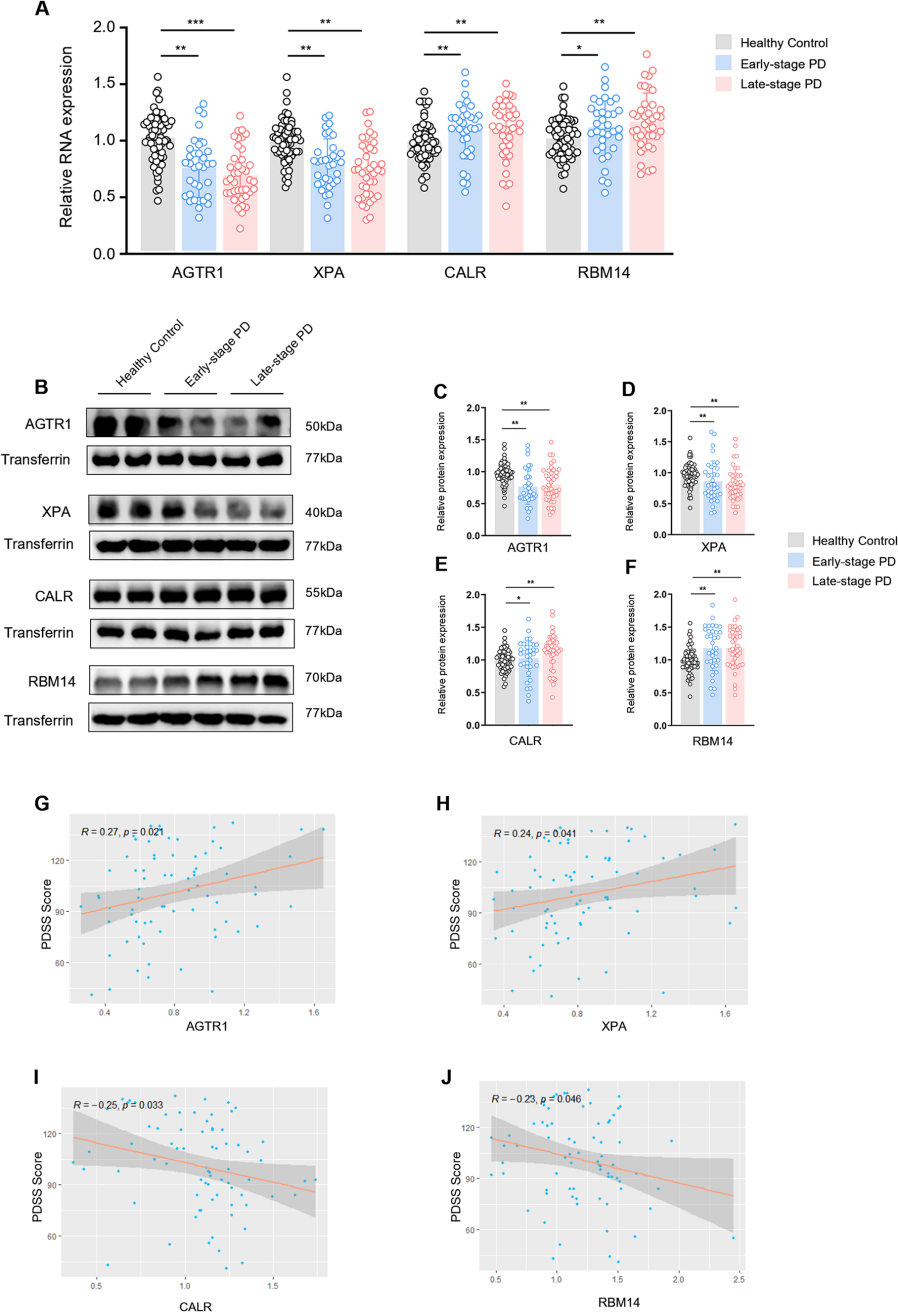
**Verification of hub gene expression and associations with circadian rhythm function (sleep disorders).** (**A**) Expression of mRNA was detected by RT‒qPCR. (**B**) Protein expression was detected by Western blotting. (**C**-**F**) Relative protein expression was calculated as the integrated density value relative to that of transferrin as a reference. (**G**-**J**) Pearson correlations between AGTR1, XPA, CALR, and RBM14 protein levels and PDSS scores. *p < 0.05, **p < 0.01, ***p < 0.001

We further verified the expression levels of relevant hub genes in blood samples obtained from 74 PD patients and 62 healthy control participants at our clinical centre. Through Western blotting and RT‒qPCR detection, we found that compared with those in the healthy control group, the expression levels of RBM14 and CALR in the PD group were increased, while AGTR1 and XPA were significantly downregulated (Fig. 10). Table 2 reveals the occurrence of significant sleep disorders in early-stage PD patients, which prompted us to conduct Pearson correlation analysis between hub gene expression and PDSS score. Our findings indicated positive correlations between AGTR1 (*R* = 0.27, *p* = 0.021) and XPA (*R* = 0.24, *p* = 0.041) and PDSS scores, while CALR (*R*=-0.25, *p* = 0.033) and RBM14 (*R*=-0.23, *p* = 0.046) exhibited negative correlations with PDSS scores, suggesting their potential role in circadian rhythm disturbance among PD patients (Fig. 10G-J).

## Discussion

As a neurodegenerative disorder, PD diagnosis currently mainly relies on clinical symptoms; however, by the time typical motor symptoms manifest, significant disease progression may have already occurred, leading to delayed treatment initiation [20]. Our clinical observational study revealed that circadian rhythm disturbance exists in the early stage of PD. The identification of these potential DE-CRRGs through our study holds significant implications for elucidating the mechanisms underlying the early-stage pathogenesis of PD. To the best of our knowledge, this is the first study to identify genes related to the circadian rhythm in PD through bioinformatics followed by machine learning techniques. The LASSO algorithm followed by logistic regression enabled us to identify 4 hub genes, which were subsequently utilized for developing a diagnostic model for PD. Additionally, we confirmed the protein expression of these hub genes in blood samples.

In this study, we found that sleep disorders exist in early-stage PD patients, which is consistent with previous research findings [10, 21]. Notably, the incidence of sleep fragmentation was found to be as high as 52.48% among PD patients, with a higher prevalence observed in late-stage PD individuals than in early-stage PD individuals. Sleep periodic limb movement, characterized by repetitive episodes of rigid flexion movements primarily affecting the lower limbs during the first half of the night, represents another significant indicator of sleep disturbance [22]. Previous studies have reported a higher occurrence of PLM in PD patients, either alone or cooccurring with restless legs syndrome (RLS) [23]. Covassin et al. demonstrated a positive correlation between PLM severity and Unified Parkinson’s Disease Rating Scale (UPDRS) III scores [24]. Similarly, severe PLM also tended to be associated with more pronounced motor symptoms in PD patients according to questionnaire assessments [25]. These findings further support an intimate relationship between PLM and disease progression within the context of PD pathology. Consistent with prior studies, our study revealed a higher prevalence of PLM among late-stage PD patients than among early-stage PD patients.

Furthermore, a higher prevalence of SDB was observed in PD patients at a late stage, potentially attributed to pharyngeal muscle tissue dysfunction resulting in upper airway obstruction. A previous study reported that the likelihood of obstructive sleep apnoea (OSA) is more than three times higher in individuals with laryngeal motor dysfunction than in those without [26], indicating that laryngeal motor dysfunction may be associated with the development of SDB. Interestingly, no significant increase in SDB incidence was observed among early-stage PD patients compared to healthy controls. We hypothesize that PD is a chronic neurodegenerative disorder characterized by α-synuclein accumulation, mitochondrial dysfunction, neuronal degeneration, and subsequent muscle motor impairment—a gradual process [27]. In turn, hypoxia during sleep can also elevate α-synuclein levels in individuals with respiratory sleep disorders [28, 29]; thus, SDB may also exacerbate or accelerate the progression of PD. Consequently, there exists an intricate interplay between PD and sleep apnoea disorders that might exhibit cumulative effects, particularly during later stages of the disease.

In terms of HRV, our findings indicated that patients with both early and late stages of PD exhibited significantly reduced SDNN only during nighttime, suggesting impaired autonomic function compared to healthy controls. However, no differences in HRV were observed during the day in this study. We speculate that increased vagus nerve excitability at night may contribute to the detectable differences in HRV at night among PD patients and healthy controls. A recent study also revealed significant reductions in HRV indicators, including SDNN, low-frequency, and high-frequency, among tremor-dominant PD patients in the early stage compared to healthy controls [30]. Tomomichi et al. found a close relationship between HRV and the degree of striatal dopamine consumption in PD patients, indirectly indicating an association between HRV and disease progression in PD [31]. Overall, our findings revealed circadian rhythm disturbance characterized by sleep disorders, changes in blood pressure rhythm, and reduced HRV emerging in early-stage PD patients, suggesting the potential involvement of circadian rhythm-related mechanisms in the early stage of PD.

AGTR1 belongs to the angiotensin group of G-protein-coupled receptors and serves as a potent primary regulator of vasopressor hormone and aldosterone secretion [32]. A previous study based on postmortem in vitro autoradiography on brains from late-stage PD patients revealed reduced angiotensin receptor content in the SNpc and striatum of PD patients [33]. Kamath et al. also identified that the SOC6_AGTR1 subgroup of neurons exhibited the most significant loss in PD patients through the mononuclear RNA test area of human SNpc dopaminergic neurons, suggesting a potential association between AGTR1 expression and susceptibility to neurodegeneration [34]. Consistent with previous research, we also observed a significant downregulation of AGTR1 expression in PD patients compared to healthy controls. Activation of AGTR1 by the brain renin–angiotensin system (RAS) is believed to promote oxidative stress and inflammatory responses, thereby accelerating the degeneration of dopaminergic neurons [35–37]. Angiotensin was also found to enhance the toxic effect of 6-OHDA in rat models treated with 6-OHDA-induced PD [38]. The AGTR1 antagonist ZD 7155 effectively attenuated 6-OHDA-induced lipid peroxidation and protein oxidation while concurrently mitigating the degeneration of dopaminergic neurons [39]. Moreover, AGTR1 activation was found to be closely associated with the upregulation of NLRP3, pro-IL-18, and other components implicated in inflammasome activation [40]. Recent clinical studies have focused on investigating the potential therapeutic and protective effects of angiotensin antagonists in patients with PD [41–43]. It has been observed that treatment with AGTR1 antagonists significantly reduces the total UPDRS score after one year in PD patients with hypertension, whereas this effect is not observed in patients receiving angiotensin-converting enzyme (ACE) inhibitor treatment [41]. These findings suggest a potential neuroprotective role of AGTR1 antagonists in PD. Simultaneously, renin angiotensin receptors exhibit a close association with circadian rhythm function, particularly in relation to blood pressure rhythm [44]. Therefore, conducting further investigations into the intricate mechanism linking renin angiotensin and the circadian rhythm of blood pressure during the early stage of PD holds immense significance in unravelling the underlying mechanisms behind its initial pathogenesis.

In addition, we observed a significant downregulation of XPA in PD patients compared to healthy controls. XPA is a gene that encodes a zinc finger protein and plays a crucial role in nucleotide excision repair, which ensures genomic stability for healthy ageing and cognitive maintenance [45]. Mutations in genes encoding crucial DNA repair proteins can lead to diseases characterized by accelerated ageing phenotypes [46]. XPA-deficient cells exhibit deficiencies in mitochondrial autophagy, excessive division of PTEN-induced putative kinase 1 (PINK1), increased mitochondrial membrane potential, and depletion of cellular nicotinamide adenine dinucleotide (NAD+), resulting in impaired mitochondrial autophagy, disturbances in energy supply, and ultimately neuronal degeneration [46, 47]. Low expression of XPA may contribute to defects in DNA repair mechanisms, leading to mitochondrial dysfunction and subsequent degeneration within dopaminergic neurons. Consistent with previous findings, our study also revealed decreased expression levels of XPA in PD patients. Moreover, it has been discovered that the circadian oscillation characteristics exhibited by the XPA protein are vital for repairing cisplatin-induced damage through nucleotide excision repair pathways [48]. Therefore, investigating whether these circadian oscillation features are associated with clinical symptoms related to circadian disturbance observed in PD warrants further exploration.

In PD patients, we also identified two upregulated proteins, CALR and RBM14, which are associated with circadian rhythm regulation. CALR is a highly conserved chaperone protein predominantly localized in the endoplasmic reticulum (ER) and is involved in various cellular processes, including protein folding and calcium homeostasis [49]. The ER stress response often serves as a defence mechanism against the accumulation of misfolded proteins in the ER and is closely associated with neurodegenerative disorders such as PD [50, 51]. Dukes et al., through dopamine-induced ER stress response, revealed that activation of chaperones in the ER, including CALR, may accelerate PD progression via allosteric protein response [52]. Lee et al. treated mouse dopaminergic neurons with 6-hydroxydopamine hydrobromide (6-OHDA) and observed a time-dependent increase in CALR expression, suggesting its association with cell stress tolerance and cell death signalling in PD [53]. A study revealed an increased number of cholinergic interneurons expressing CALR in the striatum of cynomolgus monkey models of PD, implying that secondary changes induced by PD pathology might influence alterations in CALR expression [54]. In addition, it has been discovered that melatonin, a key regulator of circadian rhythm function, could exert its influence by interacting with membrane melatonin 1 and melatonin 2 receptors and modulating intracellular proteins such as CALR [55]. Therefore, considering the potential involvement of CALR in circadian rhythm regulation and the pathogenesis of PD, investigating CALR-related mechanisms may contribute to a deeper understanding of the underlying mechanisms behind circadian rhythm disruption in PD.

RBM14, which encodes a ribonucleoprotein that serves as a general nuclear coactivator and regulator of RNA splicing, is recruited to DNA double-strand breaks in a poly(ADP-ribose)-dependent manner [56]. DNA-dependent poly(ADP-ribose) polymerase 1 (PARP1), which plays a pivotal role in the DNA damage response network, is closely associated with the progression of neurological diseases, particularly neurodegenerative disorders such as amyotrophic lateral sclerosis and frontotemporal lobe degeneration. In PD, alongside mitochondrial dysfunction, oxidative stress levels increase, and DNA damage worsens [57]. Therefore, the activation of RBM14 may occur in response to the corresponding DNA repair process to maintain homeostasis. We also observed the upregulation of RBM14 expression in PD patients. However, limited research has been conducted on RBM14 within the field of PD; thus, further investigations are warranted to elucidate its potential function in PD.

We developed a diagnostic model based on DE-CRRGs and constructed a nomogram. Nomograms have gained significant popularity in the medical field due to their exceptional predictive value, serving as simple and convenient tools for prognosis assessments [58]. A nomogram represents a logistic regression model that calculates the probability of clinical events by assigning scores to known variables. Our constructed model exhibited robust diagnostic efficacy in both the training and external validation datasets, thereby offering valuable insights into early clinical PD diagnosis and a comprehensive understanding of CR-related mechanisms.

There are some limitations in our study. First, the sample size is relatively small, which may have impacted the extrapolation and reliability of this diagnostic model. Second, we focused solely on exploring DE-CRRGs without considering other PD biomarkers, potentially affecting the accuracy of our model to some extent. In future studies, it will be necessary to validate our model using larger datasets. Additionally, further fundamental investigations are imperative to elucidate the potential mechanisms underlying the involvement of these genes in circadian rhythm disturbance associated with PD.

## Conclusion

We observed circadian rhythm disturbance in early-stage PD patients. Four CR-related hub genes were identified by bioinformatic methods integrated with machine learning techniques. Based on these 4 hub genes, we developed a nomogram for diagnosing PD, which was preliminarily validated in training and validation sets. These findings provide novel insights into the clinical diagnosis of PD and shed light on potential CR-related mechanisms in PD.

### Electronic supplementary material

Below is the link to the electronic supplementary material.

Supplementary Material 1

Supplementary Material 2

## Data Availability

The datasets are freely available from NCBI GEO database (http://www.ncbi.nlm.nih.gov/geo); further reasonable inquiries can be directed to the corresponding author.

## Abbreviations

CR: Circadian rhythm
PD: Parkinson’s disease
GEO: Gene Expression Omnibus
DEGs: Differential expression genes
CRRGs: CR-related genes
DE-CRRGs: differentially expressed CR-related genes
LASSO: least absolute shrinkage and selection operator
AUC: area under the curve
RT-qPCR: Real-time quantitative polymerase chain reaction
SNpc: substantia nigra pars compacta
HRV: heart rate variability
PSG: polysomnography
PDSS: Parkinson’s Disease Sleep Scale
BP: Blood pressure
SBP: Systolic blood pressure
wBPV: weighted Blood pressure variability
RMSS: Root Mean Square of Successive RR Interval Differences
SDNN: Standard Deviation of all NN Intervals
WGCNA: Weighted gene co‑expression network analysis
GO: Gene Ontology function
KEGG: Kyoto Encyclopedia of Genes and Genomes
PPI: Protein-protein interaction
GSVA: Gene Set Variation Analysis
AGTR1: Angiotensin II Receptor Type 1
CALR: Calreticulin
RBM14: RNA Binding Motif Protein 14
XPA: Xeroderma Pigmentosum Group A-Complementing Protein
SDB: Sleep-disordered breathing
LED: Dopamine equivalent dose
PLM: Periodic limb movement
RLS: Restless legs syndrome
UPDRS: Unified Parkinson’s Disease Rating Scale
OSA: Obstructive sleep apnea
NLRP3: NOD-like receptor protein 3
RAS: Renin-angiotensin system
ACE: Angiotensin converting enzyme
PINK1: PTEN induced putative kinase 1
NAD: Nicotinamide adenine dinucleotide
ER: Endoplasmic reticulum
6-OHDA: 6-Hydroxydopamine hydrobromide
PARP1: Poly ADP ribose polymerase 1
