# Supplementary material for: A diagnostic model for Parkinson’s disease based on circadian rhythm-related genes
Source: J Transl Med. 2024 Jul 8;22:635. doi: 10.1186/s12967-024-05424-z (PMC11229228; doi:10.1186/s12967-024-05424-z)
Supplement: Supplementary file 1 — Supplementary Material 1 [file 12967_2024_5424_MOESM1_ESM.docx]

| **Supplement table 1. Circadian rhythm related genes from Genecards database.** | |
| --- | --- |
| Gene Symbol | Description |
| PER2 | Period Circadian Regulator 2 |
| CLOCK | Clock Circadian Regulator |
| PER3 | Period Circadian Regulator 3 |
| CRY1 | Cryptochrome Circadian Regulator 1 |
| PER1 | Period Circadian Regulator 1 |
| BMAL1 | Basic Helix-Loop-Helix ARNT Like 1 |
| CRY2 | Cryptochrome Circadian Regulator 2 |
| TIMELESS | Timeless Circadian Regulator |
| LINC-ROR | Long Intergenic Non-Protein Coding RNA, Regulator Of Reprogramming |
| CSNK1D | Casein Kinase 1 Delta |
| NR1D1 | Casein Kinase 1 Delta |
| NPAS2 | Neuronal PAS Domain Protein 2 |
| CSNK1E | Casein Kinase 1 Epsilon |
| BMAL2 | Basic Helix-Loop-Helix ARNT Like 2 |
| SCN5A | Sodium Voltage-Gated Channel Alpha Subunit 5 |
| CIART | Circadian Associated Repressor Of Transcription |
| BHLHE41 | Basic Helix-Loop-Helix Family Member E41 |
| RORA | RAR Related Orphan Receptor A |
| DBP | D-Box Binding PAR BZIP Transcription Factor |
| BHLHE40 | Basic Helix-Loop-Helix Family Member E40 |
| AANAT | Aralkylamine N-Acetyltransferase |
| NPPA | Natriuretic Peptide A |
| NR1D2 | Nuclear Receptor Subfamily 1 Group D Member 2 |
| DELEC1 | Deleted In Esophageal Cancer 1 |
| MTNR1A | Melatonin Receptor 1A |
| KCNQ1 | Potassium Voltage-Gated Channel Subfamily Q Member 1 |
| RORB | RAR Related Orphan Receptor B |
| FBXL3 | F-Box And Leucine Rich Repeat Protein 3 |
| OPN4 | Opsin 4 |
| CACNA1C | Calcium Voltage-Gated Channel Subunit Alpha1 C |
| BDNF-AS | BDNF Antisense RNA |
| PRL | Prolactin |
| NFIL3 | Nuclear Factor, Interleukin 3 Regulated |
| LMNA | Lamin A/C |
| NOCT | Nocturnin |
| SMAD5-AS1 | SMAD5 Antisense RNA 1 |
| GSK3B | Glycogen Synthase Kinase 3 Beta |
| NPY | Neuropeptide Y |
| RYR2 | Ryanodine Receptor 2 |
| CIPC | CLOCK Interacting Pacemaker |
| LEP | Leptin |
| MYBPC3 | Myosin Binding Protein C3 |
| SIRT1 | Sirtuin 1 |
| MTNR1B | Melatonin Receptor 1B |
| RORC | RAR Related Orphan Receptor C |
| RAI1 | Retinoic Acid Induced 1 |
| CERNA3 | Competing Endogenous LncRNA 3 For MiR-645 |
| PROK2 | Prokineticin 2 |
| LOC110121269 | VISTA Enhancer Hs2177 |
| PASD1 | PAS Domain Containing Repressor 1 |
| ACE | Angiotensin I Converting Enzyme |
| LINC01672 | Long Intergenic Non-Protein Coding RNA 1672 |
| RNY5 | RNA, Ro60-Associated Y5 |
| SERPINE1 | Serpin Family E Member 1 |
| INS | Insulin |
| HCRT | Hypocretin Neuropeptide Precursor |
| BTRC | Beta-Transducin Repeat Containing E3 Ubiquitin Protein Ligase |
| NMS | Neuromedin S |
| CREB1 | CAMP Responsive Element Binding Protein 1 |
| REN | Renin |
| TNF | Tumor Necrosis Factor |
| VIP | Vasoactive Intestinal Peptide |
| GH1 | Growth Hormone 1 |
| NONO | Non-POU Domain Containing Octamer Binding |
| NR3C1 | Nuclear Receptor Subfamily 3 Group C Member 1 |
| IL6 | Interleukin 6 |
| DSPS | Delayed Sleep Phase Syndrome, Susceptibility To |
| PPARGC1A | PPARG Coactivator 1 Alpha |
| CRH | Corticotropin Releasing Hormone |
| CSNK2A1 | Casein Kinase 2 Alpha 1 |
| PPARG | Peroxisome Proliferator Activated Receptor Gamma |
| TP53 | Tumor Protein P53 |
| MALAT1 | Metastasis Associated Lung Adenocarcinoma Transcript 1 |
| MAGEL2 | MAGE Family Member L2 |
| NPPB | Natriuretic Peptide B |
| FLNC | Filamin C |
| AKAP9 | A-Kinase Anchoring Protein 9 |
| HTR7 | 5-Hydroxytryptamine Receptor 7 |
| EMD | Emerin |
| SLC6A4 | Solute Carrier Family 6 Member 4 |
| HLF | HLF Transcription Factor, PAR BZIP Family Member |
| GHRL | Ghrelin And Obestatin Prepropeptide |
| CREM | CAMP Responsive Element Modulator |
| SOD2-OT1 | SOD2 Overlapping Transcript 1 |
| HCRTR2 | Hypocretin Receptor 2 |
| POMC | Proopiomelanocortin |
| THRAP3 | Thyroid Hormone Receptor Associated Protein 3 |
| PPP1CC | Protein Phosphatase 1 Catalytic Subunit Gamma |
| GATA4 | GATA Binding Protein 4 |
| RBM4 | RNA Binding Motif Protein 4 |
| CHEK2 | Checkpoint Kinase 2 |
| NAMPT | Nicotinamide Phosphoribosyltransferase |
| HDAC3 | Histone Deacetylase 3 |
| IGF1 | Insulin Like Growth Factor 1 |
| NALCN | Sodium Leak Channel, Non-Selective |
| TRPM4 | Transient Receptor Potential Cation Channel Subfamily M Member 4 |
| SPSB4 | SplA/Ryanodine Receptor Domain And SOCS Box Containing 4 |
| ALB | Albumin |
| PLAT | Plasminogen Activator, Tissue Type |
| PPARA | Peroxisome Proliferator Activated Receptor Alpha |
| EMSLR | E2F1 MRNA Stabilizing LncRNA |
| PPP1CA | Protein Phosphatase 1 Catalytic Subunit Alpha |
| PPP1CB | Protein Phosphatase 1 Catalytic Subunit Beta |
| PHLPP1 | PH Domain And Leucine Rich Repeat Protein Phosphatase 1 |
| ADCYAP1 | Adenylate Cyclase Activating Polypeptide 1 |
| DRD2 | Dopamine Receptor D2 |
| HNF4A | Hepatocyte Nuclear Factor 4 Alpha |
| H19 | H19 Imprinted Maternally Expressed Transcript |
| ASMT | Acetylserotonin O-Methyltransferase |
| KCNQ1OT1 | KCNQ1 Opposite Strand/Antisense Transcript 1 |
| EZH2 | Enhancer Of Zeste 2 Polycomb Repressive Complex 2 Subunit |
| NCOA2 | Nuclear Receptor Coactivator 2 |
| CRP | C-Reactive Protein |
| TEF | TEF Transcription Factor, PAR BZIP Family Member |
| DPYD | Dihydropyrimidine Dehydrogenase |
| KDM2A | Lysine Demethylase 2A |
| CREBBP | CREB Binding Protein |
| MIR182 | MicroRNA 182 |
| MIR494 | MicroRNA 494 |
| PRNP | Prion Protein |
| TPTEP2-CSNK1E | TPTEP2-CSNK1E Readthrough |
| TMX2-CTNND1 | TMX2-CTNND1 Readthrough (NMD Candidate) |
| HULC | Hepatocellular Carcinoma Up-Regulated Long Non-Coding RNA |
| LAMP2 | Lysosomal Associated Membrane Protein 2 |
| TRC-GCA24-1 | TRNA-Cys (GCA) 24-1 |
| EDN1 | Endothelin 1 |
| MTA1 | Metastasis Associated 1 |
| SERPINC1 | Serpin Family C Member 1 |
| SRRD | SRR1 Domain Containing |
| EP300 | E1A Binding Protein P300 |
| PIWIL2 | Piwi Like RNA-Mediated Gene Silencing 2 |
| AHR | Aryl Hydrocarbon Receptor |
| KLF9 | KLF Transcription Factor 9 |
| IMPDH2 | Inosine Monophosphate Dehydrogenase 2 |
| USP2 | Ubiquitin Specific Peptidase 2 |
| CD36 | CD36 Molecule |
| NRIP1 | Nuclear Receptor Interacting Protein 1 |
| ADIPOQ | Adiponectin, C1Q And Collagen Domain Containing |
| F7 | Coagulation Factor VII |
| KAT5 | Lysine Acetyltransferase 5 |
| RMRP | RNA Component Of Mitochondrial RNA Processing Endoribonuclease |
| DRD4 | Dopamine Receptor D4 |
| SIRT6 | Sirtuin 6 |
| CSNK2A2 | Casein Kinase 2 Alpha 2 |
| AGT | Angiotensinogen |
| ZFHX3 | Zinc Finger Homeobox 3 |
| KLF15 | KLF Transcription Factor 15 |
| UBE3A | Ubiquitin Protein Ligase E3A |
| SST | Somatostatin |
| BDNF | Brain Derived Neurotrophic Factor |
| APP | Amyloid Beta Precursor Protein |
| TCAP | Titin-Cap |
| TIPIN | TIMELESS Interacting Protein |
| PPP5C | Protein Phosphatase 5 Catalytic Subunit |
| ASS1 | Argininosuccinate Synthase 1 |
| MIR21 | MicroRNA 21 |
| LEPQTL1 | Leptin, Serum Levels Of |
| TPH1 | Tryptophan Hydroxylase 1 |
| SNORD15A | Small Nucleolar RNA, C/D Box 15A |
| IL1B | Interleukin 1 Beta |
| FBXW11 | F-Box And WD Repeat Domain Containing 11 |
| FBXL21P | F-Box And Leucine Rich Repeat Protein 21, Pseudogene |
| KCND2 | Potassium Voltage-Gated Channel Subfamily D Member 2 |
| APOE | Apolipoprotein E |
| RBM4B | RNA Binding Motif Protein 4B |
| NR0B2 | Nuclear Receptor Subfamily 0 Group B Member 2 |
| PCA3 | Prostate Cancer Associated 3 |
| GCG | Glucagon |
| VIPR2 | Vasoactive Intestinal Peptide Receptor 2 |
| NR3C2 | Nuclear Receptor Subfamily 3 Group C Member 2 |
| PTH | Parathyroid Hormone |
| MAPK1 | Mitogen-Activated Protein Kinase 1 |
| KDM5A | Lysine Demethylase 5A |
| CUL1 | Cullin 1 |
| ADRB1 | Adrenoceptor Beta 1 |
| GNAQ | G Protein Subunit Alpha Q |
| NCOR1 | Nuclear Receptor Corepressor 1 |
| BGLAP | Bone Gamma-Carboxyglutamate Protein |
| VWF | Von Willebrand Factor |
| CHEK1 | Checkpoint Kinase 1 |
| HTR1A | 5-Hydroxytryptamine Receptor 1A |
| LOC110806262 | Solute Carrier Family 6 Member 4 Gene Promoter |
| GJA1 | Gap Junction Protein Alpha 1 |
| NOS3 | Nitric Oxide Synthase 3 |
| TARDBP | TAR DNA Binding Protein |
| NPY5R | Neuropeptide Y Receptor Y5 |
| GNRH1 | Gonadotropin Releasing Hormone 1 |
| PF4 | Platelet Factor 4 |
| XK | X-Linked Kx Blood Group Antigen, Kell And VPS13A Binding Protein |
| MAPK8 | Mitogen-Activated Protein Kinase 8 |
| USP9X | Ubiquitin Specific Peptidase 9 X-Linked |
| FOS | Fos Proto-Oncogene, AP-1 Transcription Factor Subunit |
| MDM2 | MDM2 Proto-Oncogene |
| METTL3 | Methyltransferase 3, N6-Adenosine-Methyltransferase Complex Catalytic Subunit |
| NDUFA9 | NADH:Ubiquinone Oxidoreductase Subunit A9 |
| ADCY10 | Adenylate Cyclase 10 |
| MLIP | Muscular LMNA Interacting Protein |
| MTOR | Mechanistic Target Of Rapamycin Kinase |
| TOP1 | DNA Topoisomerase I |
| SKP1 | S-Phase Kinase Associated Protein 1 |
| KMT2A | Lysine Methyltransferase 2A |
| EGR3 | Early Growth Response 3 |
| CDKN2A | Cyclin Dependent Kinase Inhibitor 2A |
| NAGLU | N-Acetyl-Alpha-Glucosaminidase |
| AVP | Arginine Vasopressin |
| LINC02605 | Long Intergenic Non-Protein Coding RNA 2605 |
| PML | PML Nuclear Body Scaffold |
| F3 | Coagulation Factor III, Tissue Factor |
| SFPQ | Splicing Factor Proline And Glutamine Rich |
| SELP | Selectin P |
| PGR-AS1 | PGR Antisense RNA 1 |
| GHR | Growth Hormone Receptor |
| SLX1A-SULT1A3 | SLX1A-SULT1A3 Readthrough (NMD Candidate) |
| NKX2-1 | NK2 Homeobox 1 |
| DDB1 | Damage Specific DNA Binding Protein 1 |
| SERPINA3 | Serpin Family A Member 3 |
| SPR | Sepiapterin Reductase |
| SOD1 | Superoxide Dismutase 1 |
| MIR126 | MicroRNA 126 |
| AVPR1A | Arginine Vasopressin Receptor 1A |
| HMOX1 | Heme Oxygenase 1 |
| HDAC1 | Histone Deacetylase 1 |
| MIR155 | MicroRNA 155 |
| TRE-TTC3-1 | TRNA-Glu (Anticodon TTC) 3-1 |
| GPR50 | G Protein-Coupled Receptor 50 |
| SLC12A3 | Solute Carrier Family 12 Member 3 |
| PRKAA2 | Protein Kinase AMP-Activated Catalytic Subunit Alpha 2 |
| WDR5 | WD Repeat Domain 5 |
| NODAL | Nodal Growth Differentiation Factor |
| KLF10 | KLF Transcription Factor 10 |
| EPO | Erythropoietin |
| FBN1 | Fibrillin 1 |
| USP7 | Ubiquitin Specific Peptidase 7 |
| HIF1A | Hypoxia Inducible Factor 1 Subunit Alpha |
| PROX1 | Prospero Homeobox 1 |
| MIR7-3HG | MIR7-3 Host Gene |
| PWAR1 | Prader Willi/Angelman Region RNA 1 |
| CCK | Cholecystokinin |
| OPN5 | Opsin 5 |
| TRA-TGC7-1 | TRNA-Ala (Anticodon TGC) 7-1 |
| MYC | MYC Proto-Oncogene, BHLH Transcription Factor |
| SIAH2 | Siah E3 Ubiquitin Protein Ligase 2 |
| APOB | Apolipoprotein B |
| CHKB-CPT1B | CHKB-CPT1B Readthrough (NMD Candidate) |
| PRKAA1 | Protein Kinase AMP-Activated Catalytic Subunit Alpha 1 |
| SYNE2 | Spectrin Repeat Containing Nuclear Envelope Protein 2 |
| HCCAT5 | Hepatocellular Carcinoma Associated Transcript 5 |
| PRKCG | Protein Kinase C Gamma |
| PPBP | Pro-Platelet Basic Protein |
| HUWE1 | HECT, UBA And WWE Domain Containing E3 Ubiquitin Protein Ligase 1 |
| ID2 | Inhibitor Of DNA Binding 2 |
| ADA | Adenosine Deaminase |
| CPNE8 | Copine 8 |
| NTS | Neurotensin |
| ADORA2A | Adenosine A2a Receptor |
| TAB2 | TGF-Beta Activated Kinase 1 (MAP3K7) Binding Protein 2 |
| CSNK1A1 | Casein Kinase 1 Alpha 1 |
| TET2 | Tet Methylcytosine Dioxygenase 2 |
| VCAM1 | Vascular Cell Adhesion Molecule 1 |
| MC3R | Melanocortin 3 Receptor |
| NR1H2 | Nuclear Receptor Subfamily 1 Group H Member 2 |
| KDM8 | Lysine Demethylase 8 |
| SETX | Senataxin |
| CGA | Glycoprotein Hormones, Alpha Polypeptide |
| GPR176 | G Protein-Coupled Receptor 176 |
| RGS16 | Regulator Of G Protein Signaling 16 |
| GPT | Glutamic--Pyruvic Transaminase |
| VEGFA | Vascular Endothelial Growth Factor A |
| SIN3A | SIN3 Transcription Regulator Family Member A |
| MYCBP2 | MYC Binding Protein 2 |
| SCN8A | Sodium Voltage-Gated Channel Alpha Subunit 8 |
| CYP7A1 | Cytochrome P450 Family 7 Subfamily A Member 1 |
| MIR320A | MicroRNA 320a |
| TH | Tyrosine Hydroxylase |
| KAT2B | Lysine Acetyltransferase 2B |
| CYP21A2 | Cytochrome P450 Family 21 Subfamily A Member 2 |
| OXTR | Oxytocin Receptor |
| MIR211 | MicroRNA 211 |
| ELF3 | E74 Like ETS Transcription Factor 3 |
| MT-TL1 | Mitochondrially Encoded TRNA-Leu (UUA/G) 1 |
| PSEN1 | Presenilin 1 |
| F2 | Coagulation Factor II, Thrombin |
| PRKG1 | Protein Kinase CGMP-Dependent 1 |
| MAGED1 | MAGE Family Member D1 |
| MAPK10 | Mitogen-Activated Protein Kinase 10 |
| MIR181D | MicroRNA 181d |
| CCAR2 | Cell Cycle And Apoptosis Regulator 2 |
| HNRNPU | Heterogeneous Nuclear Ribonucleoprotein U |
| ADCY1 | Adenylate Cyclase 1 |
| GHRH | Growth Hormone Releasing Hormone |
| MAPK9 | Mitogen-Activated Protein Kinase 9 |
| GRP | Gastrin Releasing Peptide |
| NPY2R | Neuropeptide Y Receptor Y2 |
| ATOH7 | Atonal BHLH Transcription Factor 7 |
| IGFBP1 | Insulin Like Growth Factor Binding Protein 1 |
| NGFR | Nerve Growth Factor Receptor |
| ID3 | Inhibitor Of DNA Binding 3 |
| FLNA | Filamin A |
| MIR132 | MicroRNA 132 |
| CNR1 | Cannabinoid Receptor 1 |
| PER3P1 | PER3 Pseudogene 1 |
| EGR1 | Early Growth Response 1 |
| LOC654780 | Splicing Factor Proline/Glutamine-Rich |
| TKT | Transketolase |
| APOH | Apolipoprotein H |
| HADHA | Hydroxyacyl-CoA Dehydrogenase Trifunctional Multienzyme Complex Subunit Alpha |
| IGFBP3 | Insulin Like Growth Factor Binding Protein 3 |
| MIR133B | MicroRNA 133b |
| LINC01587 | Long Intergenic Non-Protein Coding RNA 1587 |
| HERC2 | HECT And RLD Domain Containing E3 Ubiquitin Protein Ligase 2 |
| SERPINA6 | Serpin Family A Member 6 |
| LGR4 | Leucine Rich Repeat Containing G Protein-Coupled Receptor 4 |
| CHGA | Chromogranin A |
| FBXW7 | F-Box And WD Repeat Domain Containing 7 |
| FBXL13 | F-Box And Leucine Rich Repeat Protein 13 |
| FGA | Fibrinogen Alpha Chain |
| SHBG | Sex Hormone Binding Globulin |
| TF | Transferrin |
| ATF4 | Activating Transcription Factor 4 |
| RASD1 | Ras Related Dexamethasone Induced 1 |
| TGFB1 | Transforming Growth Factor Beta 1 |
| MIR125A | MicroRNA 125a |
| IFNG | Interferon Gamma |
| HP | Haptoglobin |
| RPS27A | Ribosomal Protein S27a |
| EPAS1 | Endothelial PAS Domain Protein 1 |
| MIR92B | MicroRNA 92b |
| HNRNPD | Heterogeneous Nuclear Ribonucleoprotein D |
| NMUR2 | Neuromedin U Receptor 2 |
| PPP2CB | Protein Phosphatase 2 Catalytic Subunit Beta |
| MECP2 | Methyl-CpG Binding Protein 2 |
| GAS5 | Growth Arrest Specific 5 |
| PTCH1 | Patched 1 |
| SIK1 | Salt Inducible Kinase 1 |
| THORLNC | Testis Associated Oncogenic LncRNA |
| CLCNKB | Chloride Voltage-Gated Channel Kb |
| SCARNA5 | Small Cajal Body-Specific RNA 5 |
| COMT | Catechol-O-Methyltransferase |
| ALG10B | ALG10 Alpha-1,2-Glucosyltransferase B |
| ACADS | Acyl-CoA Dehydrogenase Short Chain |
| ROCK2 | Rho Associated Coiled-Coil Containing Protein Kinase 2 |
| CALB1 | Calbindin 1 |
| ERC2 | ELKS/RAB6-Interacting/CAST Family Member 2 |
| CPT1A | Carnitine Palmitoyltransferase 1A |
| CSNK1A1L | Casein Kinase 1 Alpha 1 Like |
| TFPI | Tissue Factor Pathway Inhibitor |
| DCLK1 | Doublecortin Like Kinase 1 |
| UBXN11 | UBX Domain Protein 11 |
| TERT | Telomerase Reverse Transcriptase |
| PRKDC | Protein Kinase, DNA-Activated, Catalytic Subunit |
| PRKCA | Protein Kinase C Alpha |
| HSPA8 | Heat Shock Protein Family A (Hsp70) Member 8 |
| GNB2 | G Protein Subunit Beta 2 |
| AGTR1 | Angiotensin II Receptor Type 1 |
| SMC1A | Structural Maintenance Of Chromosomes 1A |
| ATR | ATR Serine/Threonine Kinase |
| MIR146B | MicroRNA 146b |
| ATG14 | Autophagy Related 14 |
| HFE | Homeostatic Iron Regulator |
| HSPD1 | Heat Shock Protein Family D (Hsp60) Member 1 |
| RXRA | Retinoid X Receptor Alpha |
| SREBF1 | Sterol Regulatory Element Binding Transcription Factor 1 |
| TFF2 | Trefoil Factor 2 |
| CDK1 | Cyclin Dependent Kinase 1 |
| SERPINF2 | Serpin Family F Member 2 |
| MIR17 | MicroRNA 17 |
| PTEN | Phosphatase And Tensin Homolog |
| PRMT5 | Protein Arginine Methyltransferase 5 |
| NEAT1 | Nuclear Paraspeckle Assembly Transcript 1 |
| SNHG14 | Small Nucleolar RNA Host Gene 14 |
| ADORA1 | Adenosine A1 Receptor |
| MIR181A1 | MicroRNA 181a-1 |
| SMAD3 | SMAD Family Member 3 |
| XIST | X Inactive Specific Transcript |
| APOA1 | Apolipoprotein A1 |
| RELA | RELA Proto-Oncogene, NF-KB Subunit |
| LEMD2 | LEM Domain Nuclear Envelope Protein 2 |
| MAOA | Monoamine Oxidase A |
| LPA | Lipoprotein(A) |
| ESR1 | Estrogen Receptor 1 |
| SGCB | Sarcoglycan Beta |
| CP | Ceruloplasmin |
| SSB | Small RNA Binding Exonuclease Protection Factor La |
| YWHAZ | Tyrosine 3-Monooxygenase/Tryptophan 5-Monooxygenase Activation Protein Zeta |
| MEG8 | Maternally Expressed 8, Small Nucleolar RNA Host Gene |
| TSPO | Translocator Protein |
| SLC2A1 | Solute Carrier Family 2 Member 1 |
| MIR145 | MicroRNA 145 |
| PROKR2 | Prokineticin Receptor 2 |
| ARNT | Aryl Hydrocarbon Receptor Nuclear Translocator |
| PSMC3 | Proteasome 26S Subunit, ATPase 3 |
| PLEC | Plectin |
| UGP2 | UDP-Glucose Pyrophosphorylase 2 |
| LEPR | Leptin Receptor |
| HTR1B | 5-Hydroxytryptamine Receptor 1B |
| TNPO1 | Transportin 1 |
| FGF21 | Fibroblast Growth Factor 21 |
| PAX8 | Paired Box 8 |
| RELB | RELB Proto-Oncogene, NF-KB Subunit |
| LINC02610 | Long Intergenic Non-Protein Coding RNA 2610 |
| GJC1 | Gap Junction Protein Gamma 1 |
| MIR96 | MicroRNA 96 |
| CEBPA | CCAAT Enhancer Binding Protein Alpha |
| LIPE | Lipase E, Hormone Sensitive Type |
| FGF23 | Fibroblast Growth Factor 23 |
| SFTPD | Surfactant Protein D |
| NGF | Nerve Growth Factor |
| HSPA1A | Heat Shock Protein Family A (Hsp70) Member 1A |
| FOXO1 | Forkhead Box O1 |
| ACHE | Acetylcholinesterase (Cartwright Blood Group) |
| SKAP1-AS2 | SKAP1 Antisense RNA 2 |
| CAVIN3 | Caveolae Associated Protein 3 |
| SERPINA12 | Serpin Family A Member 12 |
| ESR2 | Estrogen Receptor 2 |
| NCOA3 | Nuclear Receptor Coactivator 3 |
| NFE2L2 | NFE2 Like BZIP Transcription Factor 2 |
| CYP11B2 | Cytochrome P450 Family 11 Subfamily B Member 2 |
| KLK3 | Kallikrein Related Peptidase 3 |
| RNASE3 | Ribonuclease A Family Member 3 |
| NOS2 | Nitric Oxide Synthase 2 |
| IFNA1 | Interferon Alpha 1 |
| CTNNB1 | Catenin Beta 1 |
| GAST | Gastrin |
| PYY | Peptide YY |
| TNFRSF11B | TNF Receptor Superfamily Member 11b |
| HSD11B2 | Hydroxysteroid 11-Beta Dehydrogenase 2 |
| HSP90AA1 | Heat Shock Protein 90 Alpha Family Class A Member 1 |
| GPI | Glucose-6-Phosphate Isomerase |
| TOP2A | DNA Topoisomerase II Alpha |
| G6PD | Glucose-6-Phosphate Dehydrogenase |
| MMP9 | Matrix Metallopeptidase 9 |
| TBL1XR1 | TBL1X/Y Related 1 |
| FKBP1B | FKBP Prolyl Isomerase 1B |
| GNB3 | G Protein Subunit Beta 3 |
| MIR34A | MicroRNA 34a |
| PSMC4 | Proteasome 26S Subunit, ATPase 4 |
| RETN | Resistin |
| CAT | Catalase |
| MIF | Macrophage Migration Inhibitory Factor |
| PDE6B | Phosphodiesterase 6B |
| MTHFR | Methylenetetrahydrofolate Reductase |
| MEF2C | Myocyte Enhancer Factor 2C |
| TERC | Telomerase RNA Component |
| MIR183 | MicroRNA 183 |
| CSF3 | Colony Stimulating Factor 3 |
| CYP2D6 | Cytochrome P450 Family 2 Subfamily D Member 6 |
| IGF2-AS | IGF2 Antisense RNA |
| LGALS3 | Galectin 3 |
| PLCB4 | Phospholipase C Beta 4 |
| THBD | Thrombomodulin |
| LMAN1 | Lectin, Mannose Binding 1 |
| CSMD1 | CUB And Sushi Multiple Domains 1 |
| GNAS | GNAS Complex Locus |
| DDX5 | DEAD-Box Helicase 5 |
| DHX9 | DExH-Box Helicase 9 |
| SNORD118 | Small Nucleolar RNA, C/D Box 118 |
| PRRT1 | Proline Rich Transmembrane Protein 1 |
| HRH1 | Histamine Receptor H1 |
| GSR | Glutathione-Disulfide Reductase |
| HTT | Huntingtin |
| CALCA | Calcitonin Related Polypeptide Alpha |
| GCK | Glucokinase |
| IFNAR2 | Interferon Alpha And Beta Receptor Subunit 2 |
| SCN1A | Sodium Voltage-Gated Channel Alpha Subunit 1 |
| SNCA | Synuclein Alpha |
| CAMK2G | Calcium/Calmodulin Dependent Protein Kinase II Gamma |
| CAD | Carbamoyl-Phosphate Synthetase 2, Aspartate Transcarbamylase, And Dihydroorotase |
| HOTTIP | HOXA Distal Transcript Antisense RNA |
| ABCB1 | ATP Binding Cassette Subfamily B Member 1 |
| MIR142 | MicroRNA 142 |
| FHIT | Fragile Histidine Triad Diadenosine Triphosphatase |
| MAPK3 | Mitogen-Activated Protein Kinase 3 |
| NCAM1 | Neural Cell Adhesion Molecule 1 |
| DYRK1A | Dual Specificity Tyrosine Phosphorylation Regulated Kinase 1A |
| B2M | Beta-2-Microglobulin |
| PAPPA-AS1 | PAPPA Antisense RNA 1 |
| EPX | Eosinophil Peroxidase |
| ELK1 | ETS Transcription Factor ELK1 |
| SCN9A | Sodium Voltage-Gated Channel Alpha Subunit 9 |
| PRRT2 | Proline Rich Transmembrane Protein 2 |
| BVES | Blood Vessel Epicardial Substance |
| HRAS | HRas Proto-Oncogene, GTPase |
| NPY1R | Neuropeptide Y Receptor Y1 |
| MFSD2B | MFSD2 Lysolipid Transporter B, Sphingolipid |
| PEX1 | Peroxisomal Biogenesis Factor 1 |
| MIR451A | MicroRNA 451a |
| IL3 | Interleukin 3 |
| GABBR1 | Gamma-Aminobutyric Acid Type B Receptor Subunit 1 |
| NCOA1 | Nuclear Receptor Coactivator 1 |
| MED1 | Mediator Complex Subunit 1 |
| GFAP | Glial Fibrillary Acidic Protein |
| HSD3BP4 | Hydroxy-Delta-5-Steroid Dehydrogenase, 3 Beta, Pseudogene 4 |
| HTR5A | 5-Hydroxytryptamine Receptor 5A |
| HNRNPK | Heterogeneous Nuclear Ribonucleoprotein K |
| CRHR1 | Corticotropin Releasing Hormone Receptor 1 |
| IRS1 | Insulin Receptor Substrate 1 |
| SOCS1 | Suppressor Of Cytokine Signaling 1 |
| CDKN2B-AS1 | CDKN2B Antisense RNA 1 |
| PCSK9 | Proprotein Convertase Subtilisin/Kexin Type 9 |
| MIR18A | MicroRNA 18a |
| NDN | Necdin, MAGE Family Member |
| IGF2 | Insulin Like Growth Factor 2 |
| CHKA | Choline Kinase Alpha |
| GAPDH | Glyceraldehyde-3-Phosphate Dehydrogenase |
| OXT | Oxytocin/Neurophysin I Prepropeptide |
| PSPC1 | Paraspeckle Component 1 |
| CST3 | Cystatin C |
| CALR | Calreticulin |
| SLC41A1 | Solute Carrier Family 41 Member 1 |
| MATR3 | Matrin 3 |
| TXN | Thioredoxin |
| ABHD5 | Abhydrolase Domain Containing 5, Lysophosphatidic Acid Acyltransferase |
| GNS | Glucosamine (N-Acetyl)-6-Sulfatase |
| GBA1 | Glucosylceramidase Beta 1 |
| IL4 | Interleukin 4 |
| LINC00689 | Long Intergenic Non-Protein Coding RNA 689 |
| NOS1 | Nitric Oxide Synthase 1 |
| MAPT | Microtubule Associated Protein Tau |
| STX1A | Syntaxin 1A |
| APLN | Apelin |
| PSEN2 | Presenilin 2 |
| IL1RN | Interleukin 1 Receptor Antagonist |
| SCN4A | Sodium Voltage-Gated Channel Alpha Subunit 4 |
| RPE65 | Retinoid Isomerohydrolase RPE65 |
| CDK5 | Cyclin Dependent Kinase 5 |
| MT1F | Metallothionein 1F |
| TAT | Tyrosine Aminotransferase |
| PTPA | Protein Phosphatase 2 Phosphatase Activator |
| MIR29B1 | MicroRNA 29b-1 |
| ANP32A | Acidic Nuclear Phosphoprotein 32 Family Member A |
| HNRNPA1 | Heterogeneous Nuclear Ribonucleoprotein A1 |
| KISS1 | KiSS-1 Metastasis Suppressor |
| MIR155HG | MIR155 Host Gene |
| BSCL2 | BSCL2 Lipid Droplet Biogenesis Associated, Seipin |
| YTHDF2 | YTH N6-Methyladenosine RNA Binding Protein F2 |
| SUV39H1 | SUV39H1 Histone Lysine Methyltransferase |
| SLK | STE20 Like Kinase |
| CYP3A4 | Cytochrome P450 Family 3 Subfamily A Member 4 |
| ADRB2 | Adrenoceptor Beta 2 |
| AQP2 | Aquaporin 2 |
| KITLG | KIT Ligand |
| FAS-AS1 | FAS Antisense RNA 1 |
| PIWIL1 | Piwi Like RNA-Mediated Gene Silencing 1 |
| ATF1 | Activating Transcription Factor 1 |
| SDHA | Succinate Dehydrogenase Complex Flavoprotein Subunit A |
| AGRP | Agouti Related Neuropeptide |
| GRK4 | G Protein-Coupled Receptor Kinase 4 |
| ID1 | Inhibitor Of DNA Binding 1 |
| RRAS2 | RAS Related 2 |
| NR1I3 | Nuclear Receptor Subfamily 1 Group I Member 3 |
| CES1 | Carboxylesterase 1 |
| PPY | Pancreatic Polypeptide |
| STAT3 | Signal Transducer And Activator Of Transcription 3 |
| SFTA3 | Surfactant Associated 3 |
| NMU | Neuromedin U |
| SELE | Selectin E |
| FDFT1 | Farnesyl-Diphosphate Farnesyltransferase 1 |
| GLO1 | Glyoxalase I |
| FOXN3-AS1 | FOXN3 Antisense RNA 1 |
| MIR219B | MicroRNA 219b |
| HBB | Hemoglobin Subunit Beta |
| KCNC2 | Potassium Voltage-Gated Channel Subfamily C Member 2 |
| TMPO | Thymopoietin |
| UCP3 | Uncoupling Protein 3 |
| CDKN1A | Cyclin Dependent Kinase Inhibitor 1A |
| RPSA | Ribosomal Protein SA |
| NPSR1 | Neuropeptide S Receptor 1 |
| CCL2 | C-C Motif Chemokine Ligand 2 |
| USP30 | Ubiquitin Specific Peptidase 30 |
| GLUL | Glutamate-Ammonia Ligase |
| ALDOA | Aldolase, Fructose-Bisphosphate A |
| KRAS | KRAS Proto-Oncogene, GTPase |
| FNDC5 | Fibronectin Type III Domain Containing 5 |
| HK2 | Hexokinase 2 |
| CARM1 | Coactivator Associated Arginine Methyltransferase 1 |
| SYNCRIP | Synaptotagmin Binding Cytoplasmic RNA Interacting Protein |
| GSN | Gelsolin |
| PCCA | Propionyl-CoA Carboxylase Subunit Alpha |
| PCCB | Propionyl-CoA Carboxylase Subunit Beta |
| BTBD9 | BTB Domain Containing 9 |
| EIF2AK3 | Eukaryotic Translation Initiation Factor 2 Alpha Kinase 3 |
| PIK3R1 | Phosphoinositide-3-Kinase Regulatory Subunit 1 |
| KCNMA1 | Potassium Calcium-Activated Channel Subfamily M Alpha 1 |
| ZACN | Zinc Activated Ion Channel |
| MIR133A1 | MicroRNA 133a-1 |
| SMC3 | Structural Maintenance Of Chromosomes 3 |
| RAD21 | RAD21 Cohesin Complex Component |
| ZNF704 | Zinc Finger Protein 704 |
| LINC00963 | Long Intergenic Non-Protein Coding RNA 963 |
| KDM7A-DT | KDM7A Divergent Transcript |
| LINC01508 | Long Intergenic Non-Protein Coding RNA 1508 |
| CHRNA4 | Cholinergic Receptor Nicotinic Alpha 4 Subunit |
| TRH | Thyrotropin Releasing Hormone |
| LBR | Lamin B Receptor |
| ATF2 | Activating Transcription Factor 2 |
| KPNB1 | Karyopherin Subunit Beta 1 |
| CCNE1 | Cyclin E1 |
| RACK1 | Receptor For Activated C Kinase 1 |
| MDH2 | Malate Dehydrogenase 2 |
| PIGK | Phosphatidylinositol Glycan Anchor Biosynthesis Class K |
| AK5 | Adenylate Kinase 5 |
| PLCL1 | Phospholipase C Like 1 (Inactive) |
| C1orf54 | Chromosome 1 Open Reading Frame 54 |
| VTRNA3-1P | Vault RNA 3-1, Pseudogene |
| EZR | Ezrin |
| NMUR1 | Neuromedin U Receptor 1 |
| HTR2C | 5-Hydroxytryptamine Receptor 2C |
| C3orf70 | Chromosome 3 Open Reading Frame 70 |
| JAK2 | Janus Kinase 2 |
| RNASEL | Ribonuclease L |
| MIRLET7C | MicroRNA Let-7c |
| ARRB1 | Arrestin Beta 1 |
| SUV39H2 | SUV39H2 Histone Lysine Methyltransferase |
| TDRKH | Tudor And KH Domain Containing |
| NPPC | Natriuretic Peptide C |
| PNPLA3 | Patatin Like Phospholipase Domain Containing 3 |
| GIP | Gastric Inhibitory Polypeptide |
| OPN3 | Opsin 3 |
| PDE4D | Phosphodiesterase 4D |
| EYA1 | EYA Transcriptional Coactivator And Phosphatase 1 |
| OPCML | Opioid Binding Protein/Cell Adhesion Molecule Like |
| MYRIP | Myosin VIIA And Rab Interacting Protein |
| PIK3C2A | Phosphatidylinositol-4-Phosphate 3-Kinase Catalytic Subunit Type 2 Alpha |
| RNY1 | RNA, Ro60-Associated Y1 |
| PSMD11 | Proteasome 26S Subunit, Non-ATPase 11 |
| LINC01192 | Long Intergenic Non-Protein Coding RNA 1192 |
| TSC22D3 | TSC22 Domain Family Member 3 |
| LDLR | Low Density Lipoprotein Receptor |
| RFX4 | Regulatory Factor X4 |
| RNU1-1 | RNA, U1 Small Nuclear 1 |
| CD40 | CD40 Molecule |
| ALPK1 | Alpha Kinase 1 |
| A2M | Alpha-2-Macroglobulin |
| DAXX | Death Domain Associated Protein |
| TOP3A | DNA Topoisomerase III Alpha |
| MIR29A | MicroRNA 29a |
| PPOX | Protoporphyrinogen Oxidase |
| XPA | XPA, DNA Damage Recognition And Repair Factor |
| VEGFC | Vascular Endothelial Growth Factor C |
| CASR | Calcium Sensing Receptor |
| RHO | Rhodopsin |
| BAX | BCL2 Associated X, Apoptosis Regulator |
| ATP1A3 | ATPase Na+/K+ Transporting Subunit Alpha 3 |
| MEF2A | Myocyte Enhancer Factor 2A |
| HNRNPR | Heterogeneous Nuclear Ribonucleoprotein R |
| ADIPOR1 | Adiponectin Receptor 1 |
| ADIPOR2 | Adiponectin Receptor 2 |
| MAX | MYC Associated Factor X |
| CACNA1A | Calcium Voltage-Gated Channel Subunit Alpha1 A |
| BMP2 | Bone Morphogenetic Protein 2 |
| RS1 | Retinoschisin 1 |
| RRH | Retinal Pigment Epithelium-Derived Rhodopsin Homolog |
| PTPRN | Protein Tyrosine Phosphatase Receptor Type N |
| PTPRN2 | Protein Tyrosine Phosphatase Receptor Type N2 |
| CARTPT | CART Prepropeptide |
| MYH14 | Myosin Heavy Chain 14 |
| HAT1 | Histone Acetyltransferase 1 |
| GC | GC Vitamin D Binding Protein |
| FAM83B | Family With Sequence Similarity 83 Member B |
| FBXL15 | F-Box And Leucine Rich Repeat Protein 15 |
| CACUL1 | CDK2 Associated Cullin Domain 1 |
| GUSB | Glucuronidase Beta |
| HTR2A | 5-Hydroxytryptamine Receptor 2A |
| SERPINA7 | Serpin Family A Member 7 |
| SCARNA28 | Small Cajal Body-Specific RNA 28 |
| NFKB1 | Nuclear Factor Kappa B Subunit 1 |
| GCH1 | GTP Cyclohydrolase 1 |
| PROKR1 | Prokineticin Receptor 1 |
| P2RX7 | Purinergic Receptor P2X 7 |
| ACTN4 | Actinin Alpha 4 |
| GZMB | Granzyme B |
| TRP-AGG2-5 | TRNA-Pro (Anticodon AGG) 2-5 |
| MIR215 | MicroRNA 215 |
| BTNL2 | Butyrophilin Like 2 |
| IGFBP7 | Insulin Like Growth Factor Binding Protein 7 |
| RNU4ATAC | RNA, U4atac Small Nuclear |
| HMGCR | 3-Hydroxy-3-Methylglutaryl-CoA Reductase |
| GSTP1 | Glutathione S-Transferase Pi 1 |
| WAPL | WAPL Cohesin Release Factor |
| CD84 | CD84 Molecule |
| SLC7A3 | Solute Carrier Family 7 Member 3 |
| TNRC6B | Trinucleotide Repeat Containing Adaptor 6B |
| LEMD3 | LEM Domain Containing 3 |
| ALDH5A1 | Aldehyde Dehydrogenase 5 Family Member A1 |
| TLX1NB | TLX1 Neighbor |
| MMP3 | Matrix Metallopeptidase 3 |
| SUMO1 | Small Ubiquitin Like Modifier 1 |
| TGFB2 | Transforming Growth Factor Beta 2 |
| SCT | Secretin |
| CLTC | Clathrin Heavy Chain |
| RCE1 | Ras Converting CAAX Endopeptidase 1 |
| ABCB7 | ATP Binding Cassette Subfamily B Member 7 |
| IL10 | Interleukin 10 |
| MIR148A | MicroRNA 148a |
| VCP | Valosin Containing Protein |
| LRPPRC | Leucine Rich Pentatricopeptide Repeat Containing |
| CSNK2B | Casein Kinase 2 Beta |
| CSTA | Cystatin A |
| TSHB | Thyroid Stimulating Hormone Subunit Beta |
| GAL | Galanin And GMAP Prepropeptide |
| CDKN3 | Cyclin Dependent Kinase Inhibitor 3 |
| ELN | Elastin |
| ATP1A2 | ATPase Na+/K+ Transporting Subunit Alpha 2 |
| SYT10 | Synaptotagmin 10 |
| NALT1 | NOTCH1 Associated LncRNA In T Cell Acute Lymphoblastic Leukemia 1 |
| SRC | SRC Proto-Oncogene, Non-Receptor Tyrosine Kinase |
| SLC6A3 | Solute Carrier Family 6 Member 3 |
| CSF2 | Colony Stimulating Factor 2 |
| KL | Klotho |
| COG2 | Component Of Oligomeric Golgi Complex 2 |
| CEBPB | CCAAT Enhancer Binding Protein Beta |
| VRK2 | VRK Serine/Threonine Kinase 2 |
| PCAT1 | Prostate Cancer Associated Transcript 1 |
| CHRNB2 | Cholinergic Receptor Nicotinic Beta 2 Subunit |
| HSP90B1 | Heat Shock Protein 90 Beta Family Member 1 |
| TIMP3 | TIMP Metallopeptidase Inhibitor 3 |
| ATP5F1A | ATP Synthase F1 Subunit Alpha |
| INSL6 | Insulin Like 6 |
| ZPBP2 | Zona Pellucida Binding Protein 2 |
| TRPV1 | Transient Receptor Potential Cation Channel Subfamily V Member 1 |
| MIR199A1 | MicroRNA 199a-1 |
| ATF6 | Activating Transcription Factor 6 |
| PIK3R2 | Phosphoinositide-3-Kinase Regulatory Subunit 2 |
| GDF15 | Growth Differentiation Factor 15 |
| PPP2R1A | Protein Phosphatase 2 Scaffold Subunit Aalpha |
| PPP2R1B | Protein Phosphatase 2 Scaffold Subunit Abeta |
| BBOX1 | Gamma-Butyrobetaine Hydroxylase 1 |
| KCNK18 | Potassium Two Pore Domain Channel Subfamily K Member 18 |
| MLEC | Malectin |
| HSPA5 | Heat Shock Protein Family A (Hsp70) Member 5 |
| PPAT | Phosphoribosyl Pyrophosphate Amidotransferase |
| OPHN1 | Oligophrenin 1 |
| TRV-AAC1-4 | TRNA-Val (Anticodon AAC) 1-4 |
| MIR6883 | MicroRNA 6883 |
| PRKACA | Protein Kinase CAMP-Activated Catalytic Subunit Alpha |
| LOC102724058 | Uncharacterized LOC102724058 |
| COPA | COPI Coat Complex Subunit Alpha |
| RHOA | Ras Homolog Family Member A |
| HADHB | Hydroxyacyl-CoA Dehydrogenase Trifunctional Multienzyme Complex Subunit Beta |
| CYP11B1 | Cytochrome P450 Family 11 Subfamily B Member 1 |
| COPS2 | COP9 Signalosome Subunit 2 |
| GRM1 | Glutamate Metabotropic Receptor 1 |
| C1QBP | Complement C1q Binding Protein |
| MFSD2A | MFSD2 Lysolipid Transporter A, Lysophospholipid |
| UBA52 | Ubiquitin A-52 Residue Ribosomal Protein Fusion Product 1 |
| SF3A3 | Splicing Factor 3a Subunit 3 |
| CACNA1B | Calcium Voltage-Gated Channel Subunit Alpha1 B |
| YWHAE | Tyrosine 3-Monooxygenase/Tryptophan 5-Monooxygenase Activation Protein Epsilon |
| PARP1 | Poly(ADP-Ribose) Polymerase 1 |
| CYP2A6 | Cytochrome P450 Family 2 Subfamily A Member 6 |
| LCN2 | Lipocalin 2 |
| RARA | Retinoic Acid Receptor Alpha |
| PURA | Purine Rich Element Binding Protein A |
| PCNA | Proliferating Cell Nuclear Antigen |
| APH1A | Aph-1 Homolog A, Gamma-Secretase Subunit |
| TARS2 | Threonyl-TRNA Synthetase 2, Mitochondrial |
| HTR6 | 5-Hydroxytryptamine Receptor 6 |
| NBL1 | NBL1, DAN Family BMP Antagonist |
| TRAF3IP1 | TRAF3 Interacting Protein 1 |
| ASB1 | Ankyrin Repeat And SOCS Box Containing 1 |
| RASA4 | RAS P21 Protein Activator 4 |
| BOLL | Boule Homolog, RNA Binding Protein |
| NMD3 | NMD3 Ribosome Export Adaptor |
| ZNF410 | Zinc Finger Protein 410 |
| ARMC10 | Armadillo Repeat Containing 10 |
| LRRC17 | Leucine Rich Repeat Containing 17 |
| GFRAL | GDNF Family Receptor Alpha Like |
| LINC01107 | Long Intergenic Non-Protein Coding RNA 1107 |
| ENSG00000269918 | Novel Transcript |
| RNU6-234P | RNA, U6 Small Nuclear 234, Pseudogene |
| RF00017-6603 |  |
| ACP3 | Acid Phosphatase 3 |
| CYP3A5 | Cytochrome P450 Family 3 Subfamily A Member 5 |
| CTSB | Cathepsin B |
| PSME2 | Proteasome Activator Subunit 2 |
| LINC00908 | Long Intergenic Non-Protein Coding RNA 908 |
| GUCY2C | Guanylate Cyclase 2C |
| IL2 | Interleukin 2 |
| SCN11A | Sodium Voltage-Gated Channel Alpha Subunit 11 |
| IL2RA | Interleukin 2 Receptor Subunit Alpha |
| ALPP | Alkaline Phosphatase, Placental |
| TRD-GTC9-1 | TRNA-Asp (Anticodon GTC) 9-1 |
| ARHGEF5 | Rho Guanine Nucleotide Exchange Factor 5 |
| TFRC | Transferrin Receptor |
| IDS | Iduronate 2-Sulfatase |
| BLK | BLK Proto-Oncogene, Src Family Tyrosine Kinase |
| CLN5 | CLN5 Intracellular Trafficking Protein |
| BTBD10 | BTB Domain Containing 10 |
| SLC12A2 | Solute Carrier Family 12 Member 2 |
| CPSF6 | Cleavage And Polyadenylation Specific Factor 6 |
| IPW | Imprinted In Prader-Willi Syndrome |
| HS3ST2 | Heparan Sulfate-Glucosamine 3-Sulfotransferase 2 |
| MT-TP | Mitochondrially Encoded TRNA-Pro (CCN) |
| IGF1R | Insulin Like Growth Factor 1 Receptor |
| TWIST2 | Twist Family BHLH Transcription Factor 2 |
| UBE2B | Ubiquitin Conjugating Enzyme E2 B |
| CCDC81 | Coiled-Coil Domain Containing 81 |
| MBD5 | Methyl-CpG Binding Domain Protein 5 |
| RAB3A | RAB3A, Member RAS Oncogene Family |
| ADAR | Adenosine Deaminase RNA Specific |
| PTGDS | Prostaglandin D2 Synthase |
| PEPC | Peptidase C |
| ITGA2B | Integrin Subunit Alpha 2b |
| PDC | Phosducin |
| EEF2 | Eukaryotic Translation Elongation Factor 2 |
| FASN | Fatty Acid Synthase |
| MCM3 | Minichromosome Maintenance Complex Component 3 |
| PAICS | Phosphoribosylaminoimidazole Carboxylase And Phosphoribosylaminoimidazolesuccinocarboxamide Synthase |
| CCT2 | Chaperonin Containing TCP1 Subunit 2 |
| CCT8 | Chaperonin Containing TCP1 Subunit 8 |
| HNRNPH2 | Heterogeneous Nuclear Ribonucleoprotein H2 |
| HNRNPA3 | Heterogeneous Nuclear Ribonucleoprotein A3 |
| CKAP4 | Cytoskeleton Associated Protein 4 |
| CDKL5 | Cyclin Dependent Kinase Like 5 |
| P2RX5-TAX1BP3 | P2RX5-TAX1BP3 Readthrough (NMD Candidate) |
| SLC2A2 | Solute Carrier Family 2 Member 2 |
| MLN | Motilin |
| MPO | Myeloperoxidase |
| HES1 | Hes Family BHLH Transcription Factor 1 |
| FCGRT | Fc Gamma Receptor And Transporter |
| ATM | ATM Serine/Threonine Kinase |
| DNAH8 | Dynein Axonemal Heavy Chain 8 |
| HSD11B1 | Hydroxysteroid 11-Beta Dehydrogenase 1 |
| SNAI1 | Snail Family Transcriptional Repressor 1 |
| HSF1 | Heat Shock Transcription Factor 1 |
| GGT1 | Gamma-Glutamyltransferase 1 |
| CCND1 | Cyclin D1 |
| HSPG2 | Heparan Sulfate Proteoglycan 2 |
| GLUD1 | Glutamate Dehydrogenase 1 |
| ADRA1B | Adrenoceptor Alpha 1B |
| AQP3 | Aquaporin 3 (Gill Blood Group) |
| ERN1 | Endoplasmic Reticulum To Nucleus Signaling 1 |
| TNFRSF1B | TNF Receptor Superfamily Member 1B |
| UBA1 | Ubiquitin Like Modifier Activating Enzyme 1 |
| KRT17 | Keratin 17 |
| CD63 | CD63 Molecule |
| CSNK1G2 | Casein Kinase 1 Gamma 2 |
| SERPINA1 | Serpin Family A Member 1 |
| TRIM28 | Tripartite Motif Containing 28 |
| RPS6 | Ribosomal Protein S6 |
| CSE1L | Chromosome Segregation 1 Like |
| CXCL8 | C-X-C Motif Chemokine Ligand 8 |
| RPS3 | Ribosomal Protein S3 |
| LPL | Lipoprotein Lipase |
| MSN | Moesin |
| RDX | Radixin |
| RND3 | Rho Family GTPase 3 |
| RBM14 | RNA Binding Motif Protein 14 |
| PEMT | Phosphatidylethanolamine N-Methyltransferase |
| PKM | Pyruvate Kinase M1/2 |
| COL2A1 | Collagen Type II Alpha 1 Chain |
| NECTIN1 | Nectin Cell Adhesion Molecule 1 |
| MIR16-1 | MicroRNA 16-1 |
| FST | Follistatin |
| LIN28A | Lin-28 Homolog A |
| ADSL | Adenylosuccinate Lyase |
| SLC25A17 | Solute Carrier Family 25 Member 17 |
| YIF1A | Yip1 Interacting Factor Homolog A, Membrane Trafficking Protein |
| RNF157 | Ring Finger Protein 157 |
| MYOD1 | Myogenic Differentiation 1 |
| STAR | Steroidogenic Acute Regulatory Protein |
| SPSB1 | SplA/Ryanodine Receptor Domain And SOCS Box Containing 1 |
| MAPK14 | Mitogen-Activated Protein Kinase 14 |
| P4HB | Prolyl 4-Hydroxylase Subunit Beta |
| CYB5R3 | Cytochrome B5 Reductase 3 |
| KRT6A | Keratin 6A |
| MCCC1 | Methylcrotonyl-CoA Carboxylase Subunit 1 |
| CA6 | Carbonic Anhydrase 6 |
| ABAT | 4-Aminobutyrate Aminotransferase |
| HSPD1P15 | Heat Shock Protein Family D (Hsp60) Member 1 Pseudogene 15 |
| TYMS | Thymidylate Synthetase |
| TAC1 | Tachykinin Precursor 1 |
| IL7 | Interleukin 7 |
| SLC5A1 | Solute Carrier Family 5 Member 1 |
| CS | Citrate Synthase |
| HNF1A | HNF1 Homeobox A |
| UCP2 | Uncoupling Protein 2 |
| S100B | S100 Calcium Binding Protein B |
| PPARD | Peroxisome Proliferator Activated Receptor Delta |
| TRAPPC9 | Trafficking Protein Particle Complex Subunit 9 |
| WNT10A | Wnt Family Member 10A |
| ST8SIA2 | ST8 Alpha-N-Acetyl-Neuraminide Alpha-2,8-Sialyltransferase 2 |
| VRK3 | VRK Serine/Threonine Kinase 3 |
| CT69 | Cancer/Testis Associated Transcript 69 |
| LINC01193 | Long Intergenic Non-Protein Coding RNA 1193 |
| IL13 | Interleukin 13 |
| PLAU | Plasminogen Activator, Urokinase |
| GSK3A | Glycogen Synthase Kinase 3 Alpha |
| DYRK1B | Dual Specificity Tyrosine Phosphorylation Regulated Kinase 1B |
| SIX1 | SIX Homeobox 1 |
| SIX2 | SIX Homeobox 2 |
| FXN | Frataxin |
| PTGDR | Prostaglandin D2 Receptor |
| ATRIP | ATR Interacting Protein |
| GRK1 | G Protein-Coupled Receptor Kinase 1 |
| ARID1A | AT-Rich Interaction Domain 1A |
| MAT2A | Methionine Adenosyltransferase 2A |
| DDC | Dopa Decarboxylase |
| TXNIP | Thioredoxin Interacting Protein |
| FBXO5 | F-Box Protein 5 |
| TRAT1 | T Cell Receptor Associated Transmembrane Adaptor 1 |
| NOTCH1 | Notch Receptor 1 |
| OPN1LW | Opsin 1, Long Wave Sensitive |
| PDE4A | Phosphodiesterase 4A |
| CYP8B1 | Cytochrome P450 Family 8 Subfamily B Member 1 |
| H3-3B | H3.3 Histone B |
| HTR3A | 5-Hydroxytryptamine Receptor 3A |
| NR6A1 | Nuclear Receptor Subfamily 6 Group A Member 1 |
| ADM | Adrenomedullin |
| APOA4 | Apolipoprotein A4 |
| RPL3 | Ribosomal Protein L3 |
| MSRA | Methionine Sulfoxide Reductase A |
| SCYL1 | SCY1 Like Pseudokinase 1 |
| PSME4 | Proteasome Activator Subunit 4 |
| RPRD2 | Regulation Of Nuclear Pre-MRNA Domain Containing 2 |
| TOX3 | TOX High Mobility Group Box Family Member 3 |
| CASC16 | Cancer Susceptibility 16 |
| NTRK1 | Neurotrophic Receptor Tyrosine Kinase 1 |
| PRKCB | Protein Kinase C Beta |
| TRPA1 | Transient Receptor Potential Cation Channel Subfamily A Member 1 |
| EPB41 | Erythrocyte Membrane Protein Band 4.1 |
| FANCL | FA Complementation Group L |
| GRM7 | Glutamate Metabotropic Receptor 7 |
| CBLB | Cbl Proto-Oncogene B |
| GRIK1 | Glutamate Ionotropic Receptor Kainate Type Subunit 1 |
| HABP2 | Hyaluronan Binding Protein 2 |
| PTPRT | Protein Tyrosine Phosphatase Receptor Type T |
| AFF2 | ALF Transcription Elongation Factor 2 |
| COL9A1 | Collagen Type IX Alpha 1 Chain |
| MMADHC | Metabolism Of Cobalamin Associated D |
| SLC10A2 | Solute Carrier Family 10 Member 2 |
| ST3GAL1 | ST3 Beta-Galactoside Alpha-2,3-Sialyltransferase 1 |
| DLGAP1 | DLG Associated Protein 1 |
| DOCK4 | Dedicator Of Cytokinesis 4 |
| DSCAM | DS Cell Adhesion Molecule |
| NTM | Neurotrimin |
| RBCK1 | RANBP2-Type And C3HC4-Type Zinc Finger Containing 1 |
| ANKS1B | Ankyrin Repeat And Sterile Alpha Motif Domain Containing 1B |
| CD180 | CD180 Molecule |
| RGS6 | Regulator Of G Protein Signaling 6 |
| RNF19A | Ring Finger Protein 19A, RBR E3 Ubiquitin Protein Ligase |
| SORCS1 | Sortilin Related VPS10 Domain Containing Receptor 1 |
| SEC23IP | SEC23 Interacting Protein |
| ING3 | Inhibitor Of Growth Family Member 3 |
| ITPK1 | Inositol-Tetrakisphosphate 1-Kinase |
| LRRC4C | Leucine Rich Repeat Containing 4C |
| ARL4A | ADP Ribosylation Factor Like GTPase 4A |
| FOXN3 | Forkhead Box N3 |
| IRX1 | Iroquois Homeobox 1 |
| LCORL | Ligand Dependent Nuclear Receptor Corepressor Like |
| NAALADL2 | N-Acetylated Alpha-Linked Acidic Dipeptidase Like 2 |
| NCOA7 | Nuclear Receptor Coactivator 7 |
| POF1B | POF1B Actin Binding Protein |
| ATP10B | ATPase Phospholipid Transporting 10B (Putative) |
| PELI2 | Pellino E3 Ubiquitin Protein Ligase Family Member 2 |
| SPCS3 | Signal Peptidase Complex Subunit 3 |
| TANC2 | Tetratricopeptide Repeat, Ankyrin Repeat And Coiled-Coil Containing 2 |
| VTA1 | Vesicle Trafficking 1 |
| ADGRF1 | Adhesion G Protein-Coupled Receptor F1 |
| BRMS1L | BRMS1 Like Transcriptional Repressor |
| MAGEC2 | MAGE Family Member C2 |
| NXPH1 | Neurexophilin 1 |
| RASSF8 | Ras Association Domain Family Member 8 |
| RHBDD1 | Rhomboid Domain Containing 1 |
| TXNDC2 | Thioredoxin Domain Containing 2 |
| KCTD8 | Potassium Channel Tetramerization Domain Containing 8 |
| MAP9 | Microtubule Associated Protein 9 |
| FERD3L | Fer3 Like BHLH Transcription Factor |
| ZNF662 | Zinc Finger Protein 662 |
| HDX | Highly Divergent Homeobox |
| OR10R2 | Olfactory Receptor Family 10 Subfamily R Member 2 |
| CTXN3 | Cortexin 3 |
| CDC14C | Cell Division Cycle 14C |
| LINC01235 | Long Intergenic Non-Protein Coding RNA 1235 |
| RPL21P28 | Ribosomal Protein L21 Pseudogene 28 |
| RPL13AP15 | Ribosomal Protein L13a Pseudogene 15 |
| GAPDHP66 | Glyceraldehyde-3-Phosphate Dehydrogenase Pseudogene 66 |
| PA2G4P5 | Proliferation-Associated 2G4 Pseudogene 5 |
| ADH5P2 | ADH5 Pseudogene 2 |
| COX5BP1 | Cytochrome C Oxidase Subunit 5B Pseudogene 1 |
| MRPL2P1 | Mitochondrial Ribosomal Protein L2 Pseudogene 1 |
| RPL7P20 | Ribosomal Protein L7 Pseudogene 20 |
| RPS12P4 | Ribosomal Protein S12 Pseudogene 4 |
| SLC25A5P2 | Solute Carrier Family 25 Member 5 Pseudogene 2 |
| UBBP1 | Ubiquitin B Pseudogene 1 |
| ATP5MGP4 | ATP Synthase Membrane Subunit G Pseudogene 4 |
| FAM3C2P | Family With Sequence Similarity 3 Member C2, Pseudogene |
| HSPA8P1 | Heat Shock Protein Family A (Hsp70) Member 8 Pseudogene 1 |
| RPL17P25 | Ribosomal Protein L17 Pseudogene 25 |
| RPL18P5 | Ribosomal Protein L18 Pseudogene 5 |
| RPL7AP56 | Ribosomal Protein L7a Pseudogene 56 |
| RPS3AP22 | RPS3A Pseudogene 22 |
| ATP5F1AP2 | ATP Synthase F1 Subunit Alpha Pseudogene 2 |
| TRE-CTC4-1 | TRNA-Glu (CTC) 4-1 |
| DRD1 | Dopamine Receptor D1 |
| NR1H3 | Nuclear Receptor Subfamily 1 Group H Member 3 |
| KCNA2 | Potassium Voltage-Gated Channel Subfamily A Member 2 |
| ETS1 | ETS Proto-Oncogene 1, Transcription Factor |
| TFEB | Transcription Factor EB |
| PPP2CA | Protein Phosphatase 2 Catalytic Subunit Alpha |
| PPP2R5D | Protein Phosphatase 2 Regulatory Subunit B'Delta |
| PPP2R5E | Protein Phosphatase 2 Regulatory Subunit B'Epsilon |
| DOK7 | Docking Protein 7 |
| TYR | Tyrosinase |
| CNR2 | Cannabinoid Receptor 2 |
| KHK | Ketohexokinase |
| MPST | Mercaptopyruvate Sulfurtransferase |
| MYBL2 | MYB Proto-Oncogene Like 2 |
| HSD17B8 | Hydroxysteroid 17-Beta Dehydrogenase 8 |
| LGALS4 | Galectin 4 |
| ZNF346 | Zinc Finger Protein 346 |
| GJE1 | Gap Junction Protein Epsilon 1 |
| HNRNPL | Heterogeneous Nuclear Ribonucleoprotein L |
| APC | APC Regulator Of WNT Signaling Pathway |
| CCNB1 | Cyclin B1 |
| BSX | Brain Specific Homeobox |
| LOC110740340 | CYP2D6 Promoter |
| XBP1 | X-Box Binding Protein 1 |
| FGFR1 | Fibroblast Growth Factor Receptor 1 |
| SHH | Sonic Hedgehog Signaling Molecule |
| FGF8 | Fibroblast Growth Factor 8 |
| GLI2 | GLI Family Zinc Finger 2 |
| CDON | Cell Adhesion Associated, Oncogene Regulated |
| TGIF1 | TGFB Induced Factor Homeobox 1 |
| STAG2 | STAG2 Cohesin Complex Component |
| DLL1 | Delta Like Canonical Notch Ligand 1 |
| GAS1 | Growth Arrest Specific 1 |
| PLCH1 | Phospholipase C Eta 1 |
| SIX3 | SIX Homeobox 3 |
| ZIC2 | Zic Family Member 2 |
| FOXH1 | Forkhead Box H1 |
| CRIPTO | Cripto, EGF-CFC Family Member |
| DISP1 | Dispatched RND Transporter Family Member 1 |
| STIL | STIL Centriolar Assembly Protein |
| TPT1 | Tumor Protein, Translationally-Controlled 1 |
| CYP2C19 | Cytochrome P450 Family 2 Subfamily C Member 19 |
| EPHB2 | EPH Receptor B2 |
| PSME1 | Proteasome Activator Subunit 1 |
| PIK3CG | Phosphatidylinositol-4,5-Bisphosphate 3-Kinase Catalytic Subunit Gamma |
| ADCYAP1R1 | ADCYAP Receptor Type I |
| GNAL | G Protein Subunit Alpha L |
| CHGB | Chromogranin B |
| IDH1 | Isocitrate Dehydrogenase (NADP(+)) 1 |
| MIR34C | MicroRNA 34c |
| MIRLET7B | MicroRNA Let-7b |
| AMH | Anti-Mullerian Hormone |
| CREB3 | CAMP Responsive Element Binding Protein 3 |
| DICER1 | Dicer 1, Ribonuclease III |
| CDK5R1 | Cyclin Dependent Kinase 5 Regulatory Subunit 1 |
| CIDEC | Cell Death Inducing DFFA Like Effector C |
| OGG1 | 8-Oxoguanine DNA Glycosylase |
| CAMK1 | Calcium/Calmodulin Dependent Protein Kinase I |
| MIR219A1 | MicroRNA 219a-1 |
| FGF19 | Fibroblast Growth Factor 19 |
| OPRM1 | Opioid Receptor Mu 1 |
| FAT1 | FAT Atypical Cadherin 1 |
| HSPE1 | Heat Shock Protein Family E (Hsp10) Member 1 |
| ACTN1 | Actinin Alpha 1 |
| PSMD2 | Proteasome 26S Subunit Ubiquitin Receptor, Non-ATPase 2 |
| TLN1 | Talin 1 |
| GFPT2 | Glutamine-Fructose-6-Phosphate Transaminase 2 |
| CAND1 | Cullin Associated And Neddylation Dissociated 1 |
| PDIA6 | Protein Disulfide Isomerase Family A Member 6 |
| OIP5-AS1 | OIP5 Antisense RNA 1 |
| CTBP1-DT | CTBP1 Divergent Transcript |
| INTS6-AS1 | INTS6 Antisense RNA 1 |
| NDUFA6-DT | NDUFA6 Divergent Transcript |
| PITPNA-AS1 | PITPNA Antisense RNA 1 |
| ICAM1 | Intercellular Adhesion Molecule 1 |
| DHCR7 | 7-Dehydrocholesterol Reductase |
| LIMK1 | LIM Domain Kinase 1 |
| TPO | Thyroid Peroxidase |
| IGFBP2 | Insulin Like Growth Factor Binding Protein 2 |
| SOS1 | SOS Ras/Rac Guanine Nucleotide Exchange Factor 1 |
| PEX14 | Peroxisomal Biogenesis Factor 14 |
| FEN1 | Flap Structure-Specific Endonuclease 1 |
| DROSHA | Drosha Ribonuclease III |
| MIR3614 | MicroRNA 3614 |
| COX7B | Cytochrome C Oxidase Subunit 7B |
| GNA11 | G Protein Subunit Alpha 11 |
| HELLS | Helicase, Lymphoid Specific |
| IL5 | Interleukin 5 |
| IRX2-DT | IRX2 Divergent Transcript |
| FOXP3 | Forkhead Box P3 |
| NEDD4L | NEDD4 Like E3 Ubiquitin Protein Ligase |
| ADRA2C | Adrenoceptor Alpha 2C |
| KNG1 | Kininogen 1 |
| TSC2 | TSC Complex Subunit 2 |
| FBP1 | Fructose-Bisphosphatase 1 |
| HLA-DRB1 | Major Histocompatibility Complex, Class II, DR Beta 1 |
| SFTPB | Surfactant Protein B |
| SFTPC | Surfactant Protein C |
| ALAS1 | 5'-Aminolevulinate Synthase 1 |
| IMMT | Inner Membrane Mitochondrial Protein |
| LINC01194 | Long Intergenic Non-Protein Coding RNA 1194 |
| PLIN1 | Perilipin 1 |
| TLR4 | Toll Like Receptor 4 |
| MC4R | Melanocortin 4 Receptor |
| IFNAR1 | Interferon Alpha And Beta Receptor Subunit 1 |
| RPA2 | Replication Protein A2 |
| DPP4 | Dipeptidyl Peptidase 4 |
| MEIS1 | Meis Homeobox 1 |
| TDO2 | Tryptophan 2,3-Dioxygenase |
| ITGB1 | Integrin Subunit Beta 1 |
| ENO2 | Enolase 2 |
| SULT1A3 | Sulfotransferase Family 1A Member 3 |
| SIK3 | SIK Family Kinase 3 |
| PAGE5 | PAGE Family Member 5 |
| IL1A | Interleukin 1 Alpha |
| IL18 | Interleukin 18 |
| TRRAP | Transformation/Transcription Domain Associated Protein |
| MT3 | Metallothionein 3 |
| RAN | RAN, Member RAS Oncogene Family |
| KDM2B | Lysine Demethylase 2B |
| FBXO9 | F-Box Protein 9 |
| FBXO22 | F-Box Protein 22 |
| COPS8 | COP9 Signalosome Subunit 8 |
| HK1 | Hexokinase 1 |
| PGK1 | Phosphoglycerate Kinase 1 |
| ACACA | Acetyl-CoA Carboxylase Alpha |
| PRKD1 | Protein Kinase D1 |
| RPS19 | Ribosomal Protein S19 |
| ENO1 | Enolase 1 |
| HMGA1 | High Mobility Group AT-Hook 1 |
| HNRNPA2B1 | Heterogeneous Nuclear Ribonucleoprotein A2/B1 |
| HSD17B10 | Hydroxysteroid 17-Beta Dehydrogenase 10 |
| DDOST | Dolichyl-Diphosphooligosaccharide--Protein Glycosyltransferase Non-Catalytic Subunit |
| VDAC1 | Voltage Dependent Anion Channel 1 |
| NME1 | NME/NM23 Nucleoside Diphosphate Kinase 1 |
| NME2 | NME/NM23 Nucleoside Diphosphate Kinase 2 |
| PRKD3 | Protein Kinase D3 |
| MCCC2 | Methylcrotonyl-CoA Carboxylase Subunit 2 |
| ERO1A | Endoplasmic Reticulum Oxidoreductase 1 Alpha |
| PSMD3 | Proteasome 26S Subunit, Non-ATPase 3 |
| CCT7 | Chaperonin Containing TCP1 Subunit 7 |
| PSMC5 | Proteasome 26S Subunit, ATPase 5 |
| CAPZB | Capping Actin Protein Of Muscle Z-Line Subunit Beta |
| EIF3E | Eukaryotic Translation Initiation Factor 3 Subunit E |
| RAB1A | RAB1A, Member RAS Oncogene Family |
| RAB5B | RAB5B, Member RAS Oncogene Family |
| SMCHD1 | Structural Maintenance Of Chromosomes Flexible Hinge Domain Containing 1 |
| TAGLN2 | Transgelin 2 |
| PSMC6 | Proteasome 26S Subunit, ATPase 6 |
| PSMD1 | Proteasome 26S Subunit, Non-ATPase 1 |
| HNRNPH3 | Heterogeneous Nuclear Ribonucleoprotein H3 |
| AHNAK | AHNAK Nucleoprotein |
| AURKAIP1 | Aurora Kinase A Interacting Protein 1 |
| RALY | RALY Heterogeneous Nuclear Ribonucleoprotein |
| RPL36 | Ribosomal Protein L36 |
| SEPTIN7 | Septin 7 |
| APMAP | Adipocyte Plasma Membrane Associated Protein |
| CPNE9 | Copine Family Member 9 |
| H2BC11 | H2B Clustered Histone 11 |
| HID1 | HID1 Domain Containing |
| MAGEC1 | MAGE Family Member C1 |
| RPL39 | Ribosomal Protein L39 |
| HMGB1P1 | High Mobility Group Box 1 Pseudogene 1 |
| RAP1BL | RAP1B Like (Pseudogene) |
| TRAF2 | TNF Receptor Associated Factor 2 |
| ORMDL3 | ORMDL Sphingolipid Biosynthesis Regulator 3 |
| GMNN | Geminin DNA Replication Inhibitor |
| TREM2 | Triggering Receptor Expressed On Myeloid Cells 2 |
| MIR22 | MicroRNA 22 |
| MIR29B2 | MicroRNA 29b-2 |
| MIR29C | MicroRNA 29c |
| CBL | Cbl Proto-Oncogene |
| TSHR | Thyroid Stimulating Hormone Receptor |
| GTF2IRD1 | GTF2I Repeat Domain Containing 1 |
| SEC16A | SEC16 Homolog A, Endoplasmic Reticulum Export Factor |
| PENK | Proenkephalin |
| CCNA2 | Cyclin A2 |
| PFAS | Phosphoribosylformylglycinamidine Synthase |
| CIRBP | Cold Inducible RNA Binding Protein |
| DIRAS3 | DIRAS Family GTPase 3 |
| SNHG7 | Small Nucleolar RNA Host Gene 7 |
| SNORD14D | Small Nucleolar RNA, C/D Box 14D |
| SNORA23 | Small Nucleolar RNA, H/ACA Box 23 |
| PDGFB | Platelet Derived Growth Factor Subunit B |
| RPS6KB1 | Ribosomal Protein S6 Kinase B1 |
| CTSL | Cathepsin L |
| CTSH | Cathepsin H |
| BLZF1 | Basic Leucine Zipper Nuclear Factor 1 |
| AMY1A | Amylase Alpha 1A |
| MIR381 | MicroRNA 381 |
| MIR300 | MicroRNA 300 |
| TIMP1 | TIMP Metallopeptidase Inhibitor 1 |
| MIR92A1 | MicroRNA 92a-1 |
| MAT1A | Methionine Adenosyltransferase 1A |
| TWIST1 | Twist Family BHLH Transcription Factor 1 |
| MAP2K7 | Mitogen-Activated Protein Kinase Kinase 7 |
| NOX4 | NADPH Oxidase 4 |
| TRN-GTT2-1 | TRNA-Asn (Anticodon GTT) 2-1 |
| KCNH4 | Potassium Voltage-Gated Channel Subfamily H Member 4 |
| ID4 | Inhibitor Of DNA Binding 4 |
| TRPV6 | Transient Receptor Potential Cation Channel Subfamily V Member 6 |
| HEBP1 | Heme Binding Protein 1 |
| JUND | JunD Proto-Oncogene, AP-1 Transcription Factor Subunit |
| CACNA1G | Calcium Voltage-Gated Channel Subunit Alpha1 G |
| ESRRB | Estrogen Related Receptor Beta |
| HBA1 | Hemoglobin Subunit Alpha 1 |
| MIAT | Myocardial Infarction Associated Transcript |
| PRKCD | Protein Kinase C Delta |
| NRG1 | Neuregulin 1 |
| EPHB1 | EPH Receptor B1 |
| GNAO1 | G Protein Subunit Alpha O1 |
| ISG15 | ISG15 Ubiquitin Like Modifier |
| PDE8B | Phosphodiesterase 8B |
| ALDH6A1 | Aldehyde Dehydrogenase 6 Family Member A1 |
| CNTN2 | Contactin 2 |
| FOXP1 | Forkhead Box P1 |
| SF3B1 | Splicing Factor 3b Subunit 1 |
| ADCY8 | Adenylate Cyclase 8 |
| CA14 | Carbonic Anhydrase 14 |
| DLX5 | Distal-Less Homeobox 5 |
| EIF2B2 | Eukaryotic Translation Initiation Factor 2B Subunit Beta |
| IL1RAPL1 | Interleukin 1 Receptor Accessory Protein Like 1 |
| NFASC | Neurofascin |
| PRPF3 | Pre-MRNA Processing Factor 3 |
| ROBO2 | Roundabout Guidance Receptor 2 |
| CCS | Copper Chaperone For Superoxide Dismutase |
| CYP51A1 | Cytochrome P450 Family 51 Subfamily A Member 1 |
| GNL3 | G Protein Nucleolar 3 |
| MANBA | Mannosidase Beta |
| NCK2 | NCK Adaptor Protein 2 |
| SLC26A5 | Solute Carrier Family 26 Member 5 |
| CRB2 | Crumbs Cell Polarity Complex Component 2 |
| FANCE | FA Complementation Group E |
| KRIT1 | KRIT1 Ankyrin Repeat Containing |
| PACS1 | Phosphofurin Acidic Cluster Sorting Protein 1 |
| PDIA2 | Protein Disulfide Isomerase Family A Member 2 |
| SF3B2 | Splicing Factor 3b Subunit 2 |
| STAB1 | Stabilin 1 |
| VAMP3 | Vesicle Associated Membrane Protein 3 |
| ARHGAP15 | Rho GTPase Activating Protein 15 |
| MLH3 | MutL Homolog 3 |
| MUS81 | MUS81 Structure-Specific Endonuclease Subunit |
| ACYP2 | Acylphosphatase 2 |
| CD248 | CD248 Molecule |
| DRG2 | Developmentally Regulated GTP Binding Protein 2 |
| POLR2J | RNA Polymerase II Subunit J |
| SART1 | Spliceosome Associated Factor 1, Recruiter Of U4/U6.U5 Tri-SnRNP |
| SGSM3 | Small G Protein Signaling Modulator 3 |
| UNC5D | Unc-5 Netrin Receptor D |
| BICC1 | BicC Family RNA Binding Protein 1 |
| DENND1A | DENN Domain Containing 1A |
| ERI1 | Exoribonuclease 1 |
| LRRN3 | Leucine Rich Repeat Neuronal 3 |
| MPRIP | Myosin Phosphatase Rho Interacting Protein |
| ORAI2 | ORAI Calcium Release-Activated Calcium Modulator 2 |
| PINX1 | PIN2 (TERF1) Interacting Telomerase Inhibitor 1 |
| PPM1L | Protein Phosphatase, Mg2+/Mn2+ Dependent 1L |
| PTGR2 | Prostaglandin Reductase 2 |
| RIN1 | Ras And Rab Interactor 1 |
| RNF10 | Ring Finger Protein 10 |
| SPDEF | SAM Pointed Domain Containing ETS Transcription Factor |
| TAF11 | TATA-Box Binding Protein Associated Factor 11 |
| HIKESHI | Heat Shock Protein Nuclear Import Factor Hikeshi |
| HORMAD1 | HORMA Domain Containing 1 |
| MPPED2 | Metallophosphoesterase Domain Containing 2 |
| NOL4 | Nucleolar Protein 4 |
| NPL | N-Acetylneuraminate Pyruvate Lyase |
| PHACTR1 | Phosphatase And Actin Regulator 1 |
| RFT1 | RFT1 Homolog |
| RGS8 | Regulator Of G Protein Signaling 8 |
| RHOD | Ras Homolog Family Member D |
| SEM1 | SEM1 26S Proteasome Subunit |
| SSH3 | Slingshot Protein Phosphatase 3 |
| ZNF76 | Zinc Finger Protein 76 |
| BRMS1 | BRMS1 Transcriptional Repressor And Anoikis Regulator |
| COQ10B | Coenzyme Q10B |
| DNAJC2 | DnaJ Heat Shock Protein Family (Hsp40) Member C2 |
| FAM167A | Family With Sequence Similarity 167 Member A |
| GTDC1 | Glycosyltransferase Like Domain Containing 1 |
| MYO15A | Myosin XVA |
| PATJ | PATJ Crumbs Cell Polarity Complex Component |
| RAX | Retina And Anterior Neural Fold Homeobox |
| SFMBT1 | Scm Like With Four Mbt Domains 1 |
| SNRPC | Small Nuclear Ribonucleoprotein Polypeptide C |
| ANKRD44 | Ankyrin Repeat Domain 44 |
| ANP32E | Acidic Nuclear Phosphoprotein 32 Family Member E |
| BLTP3A | Bridge-Like Lipid Transfer Protein Family Member 3A |
| LRWD1 | Leucine Rich Repeats And WD Repeat Domain Containing 1 |
| MTRF1L | Mitochondrial Translation Release Factor 1 Like |
| NUDT3 | Nudix Hydrolase 3 |
| PPP1R3B | Protein Phosphatase 1 Regulatory Subunit 3B |
| DNAJC16 | DnaJ Heat Shock Protein Family (Hsp40) Member C16 |
| EPC2 | Enhancer Of Polycomb Homolog 2 |
| FRMD8 | FERM Domain Containing 8 |
| ILRUN | Inflammation And Lipid Regulator With UBA-Like And NBR1-Like Domains |
| MED9 | Mediator Complex Subunit 9 |
| SEC11C | SEC11 Homolog C, Signal Peptidase Complex Subunit |
| ZCCHC2 | Zinc Finger CCHC-Type Containing 2 |
| ANKIB1 | Ankyrin Repeat And IBR Domain Containing 1 |
| BARHL2 | BarH Like Homeobox 2 |
| FCF1 | FCF1 RRNA-Processing Protein |
| LIN52 | Lin-52 DREAM MuvB Core Complex Component |
| SAMD11 | Sterile Alpha Motif Domain Containing 11 |
| EIF1AD | Eukaryotic Translation Initiation Factor 1A Domain Containing |
| ETAA1 | ETAA1 Activator Of ATR Kinase |
| FBXO34 | F-Box Protein 34 |
| PRKRIP1 | PRKR Interacting Protein 1 |
| RFTN2 | Raftlin Family Member 2 |
| SPTSSB | Serine Palmitoyltransferase Small Subunit B |
| TMEM151A | Transmembrane Protein 151A |
| WDCP | WD Repeat And Coiled Coil Containing |
| XKR6 | XK Related 6 |
| YLPM1 | YLP Motif Containing 1 |
| ZNRD2 | Zinc Ribbon Domain Containing 2 |
| CPLX4 | Complexin 4 |
| NRARP | NOTCH Regulated Ankyrin Repeat Protein |
| EXD3 | Exonuclease 3'-5' Domain Containing 3 |
| MIDEAS | Mitotic Deacetylase Associated SANT Domain Protein |
| PCNX3 | Pecanex 3 |
| RGSL1 | Regulator Of G Protein Signaling Like 1 |
| SMCR8 | SMCR8-C9orf72 Complex Subunit |
| FAM185A | Family With Sequence Similarity 185 Member A |
| SPTY2D1 | SPT2 Chromatin Protein Domain Containing 1 |
| CIMIP2A | Ciliary Microtubule Inner Protein 2A |
| POLR2J3 | RNA Polymerase II Subunit J3 |
| RASA4B | RAS P21 Protein Activator 4B |
| OR4F16 | Olfactory Receptor Family 4 Subfamily F Member 16 |
| DPY19L2P2 | DPY19L2 Pseudogene 2 |
| FAM87B | Family With Sequence Similarity 87 Member B |
| LINC00115 | Long Intergenic Non-Protein Coding RNA 115 |
| LINC00208 | Long Intergenic Non-Protein Coding RNA 208 |
| FOXP1-IT1 | FOXP1 Intronic Transcript 1 |
| IGKV1OR2-108 | Immunoglobulin Kappa Variable 1/OR2-108 (Non-Functional) |
| MIR1284 | MicroRNA 1284 |
| LINC01128 | Long Intergenic Non-Protein Coding RNA 1128 |
| MEIS1-AS2 | MEIS1 Antisense RNA 2 |
| B4GAT1-DT | B4GAT1 Divergent Transcript |
| FAM41C | Family With Sequence Similarity 41 Member C |
| ILRUN-AS1 | ILRUN Antisense RNA 1 |
| LINC01322 | Long Intergenic Non-Protein Coding RNA 1322 |
| LINC01933 | Long Intergenic Non-Protein Coding RNA 1933 |
| LINC02067 | Long Intergenic Non-Protein Coding RNA 2067 |
| MIDEAS-AS1 | MIDEAS Antisense RNA 1 |
| NRG1-IT1 | NRG1 Intronic Transcript 1 |
| BET1-AS1 | BET1 Antisense RNA 1 |
| KLC2-AS1 | KLC2 Antisense RNA 1 |
| LINC01470 | Long Intergenic Non-Protein Coding RNA 1470 |
| LINC01688 | Long Intergenic Non-Protein Coding RNA 1688 |
| LINC02609 | Long Intergenic Non-Protein Coding RNA 2609 |
| SMIM40 | Small Integral Membrane Protein 40 |
| FBXO34-AS1 | FBXO34 Antisense RNA 1 |
| LOC124902694 | Uncharacterized LOC124902694 |
| MIR548A1HG | MIR548A1 Host Gene |
| SCUBE3-AS1 | SCUBE3 Antisense RNA 1 |
| CRYZP1 | Crystallin Zeta Pseudogene 1 |
| ENSG00000228655 | Novel Transcript, Antisense To ARHGAP15 |
| FAM86B3P | Family With Sequence Similarity 86 Member B3, Pseudogene |
| LINC01241 | Long Intergenic Non-Protein Coding RNA 1241 |
| LOC101928626 | Uncharacterized LOC101928626 |
| OACYLP | O-Acyltransferase Like, Pseudogene |
| ENSG00000224984 | Novel Transcript |
| ENSG00000237301 | Novel Transcript |
| ENSG00000254458 | Novel Transcript |
| ENSG00000255038 | Novel Transcript, Antisense To SF3B2 |
| ENSG00000255310 | Novel Transcript |
| ENSG00000261116 | Novel Transcript, Overlapping To FAM83B |
| ENSG00000265511 | Novel Transcript |
| ENSG00000285708 | Novel Protein |
| HMGN1P1 | High Mobility Group Nucleosome Binding Domain 1 Pseudogene 1 |
| LINC02840 | Long Intergenic Non-Protein Coding RNA 2840 |
| LOC124900524 | Small Nucleolar RNA SNORA4 |
| RNU6-1029P | RNA, U6 Small Nuclear 1029, Pseudogene |
| RNU6-1136P | RNA, U6 Small Nuclear 1136, Pseudogene |
| RPL7AP39 | Ribosomal Protein L7a Pseudogene 39 |
| EEF1GP4 | Eukaryotic Translation Elongation Factor 1 Gamma Pseudogene 4 |
| ENSG00000236453 | Novel Transcript |
| ENSG00000236938 | Novel Transcript |
| ENSG00000243107 | Novel Transcript |
| ENSG00000254936 | Novel Transcript |
| ENSG00000260647 | Novel Transcript, Antisense To TOP3A |
| OR7E111P | Olfactory Receptor Family 7 Subfamily E Member 111 Pseudogene |
| RPL10P19 | Ribosomal Protein L10 Pseudogene 19 |
| RPL23AP95 | Ribosomal Protein L23a Pseudogene 95 |
| SERBP1P3 | SERPINE1 MRNA Binding Protein 1 Pseudogene 3 |
| SNORD3I | Small Nucleolar RNA, C/D Box 3I |
| SRSF6P2 | SRSF6 Pseudogene 2 |
| UPF3AP1 | UPF3A Pseudogene 1 |
| ACTG1P24 | Actin Gamma 1 Pseudogene 24 |
| ENSG00000226965 | Novel Transcript |
| ENSG00000253656 | Novel Transcript |
| ENSG00000269925 | Novel Transcript, Sense Intronic To VAMP3 |
| ENSG00000285953 | Novel Protein |
| LOC100419436 | Ribosomal Protein S2 Pseudogene |
| LOC128092250 | Uncharacterized LOC128092250 |
| MAPRE1P2 | MAPRE1 Pseudogene 2 |
| MARK2P10 | MARK2 Pseudogene 10 |
| NPM1P22 | Nucleophosmin 1 Pseudogene 22 |
| RN7SL714P | RNA, 7SL, Cytoplasmic 714, Pseudogene |
| RSL24D1P5 | Ribosomal L24 Domain Containing 1 Pseudogene 5 |
| SNORD13C | Small Nucleolar RNA, C/D Box 13C |
| EEF1A1P12 | Eukaryotic Translation Elongation Factor 1 Alpha 1 Pseudogene 12 |
| ENSG00000289212 | Novel Transcript |
| POLR2J3-UPK3BL2 | POLR2J3-UPK3BL2 Readthrough |
| RNA5SP228 | RNA, 5S Ribosomal Pseudogene 228 |
| RNA5SP358 | RNA, 5S Ribosomal Pseudogene 358 |
| RNU6-400P | RNA, U6 Small Nuclear 400, Pseudogene |
| RNU6-472P | RNA, U6 Small Nuclear 472, Pseudogene |
| RNU6-533P | RNA, U6 Small Nuclear 533, Pseudogene |
| SRSF3P1 | SRSF3 Pseudogene 1 |
| ENSG00000276839 | Metazoan Signal Recognition Particle RNA |
| ENSG00000289027 | Novel Protein |
| GARIN3P1 | GARIN3 Pseudogene 1 |
| lnc-ENTPD8-2 |  |
| CLUHP8 | Clustered Mitochondria Homolog Pseudogene 8 |
| ENSG00000287870 | Novel Transcript |
| ENSG00000290077 | Novel Transcript, Sense Intronic To RP11-79P21.2and FOXP1 |
| HSALNG0007013 |  |
| HSALNG0026758 |  |
| LOC124900511 | Uncharacterized LOC124900511 |
| MK280269-045 |  |
| NONHSAG035203.2 |  |
| RF00017-5502 |  |
| SNODB424 |  |
| lnc-CLDN23-8 |  |
| lnc-ENTPD8-1 |  |
| lnc-KYNU-7 |  |
| lnc-NAPEPLD-7 |  |
| lnc-NOC2L-9 |  |
| lnc-PER2-5 |  |
| lnc-SF3B1-5 |  |
| piR-61101-085 |  |
| CM034951-004 |  |
| ENSG00000274346 | Novel Zinc Finger Protein Pseudogene |
| ENSG00000289560 | Novel Transcript |
| HSALNG0008944 |  |
| HSALNG0009912 |  |
| HSALNG0018052 |  |
| HSALNG0030254 |  |
| HSALNG0060181 |  |
| HSALNG0060516-001 |  |
| HSALNG0063445 |  |
| HSALNG0102409 |  |
| HSALNG0135545 |  |
| HSALNG0135546 |  |
| HSALNG0150020 |  |
| KR153203-015 |  |
| LOC105375451 | Uncharacterized LOC105375451 |
| LOC105378808 | Uncharacterized LOC105378808 |
| LOC105379241 | Uncharacterized LOC105379241 |
| LOC107986003 | Uncharacterized LOC107986003 |
| LOC124901540 | Small Nucleolar RNA U13 |
| MK280144-567 |  |
| RF00017-5506 |  |
| RF00017-8175 |  |
| lnc-CRB2-4 |  |
| lnc-LRRD1-4 |  |
| lnc-NOXA1-1 |  |
| lnc-WRN-10 |  |
| piR-33303-073 |  |
| piR-44085-015 |  |
| piR-48867-002 |  |
| piR-49018 |  |
| piR-57138-015 |  |
| piR-58312 |  |
| CM034957-286 |  |
| HSALNG0004569 |  |
| HSALNG0050748 |  |
| HSALNG0060208 |  |
| HSALNG0060548 |  |
| HSALNG0063404 |  |
| HSALNG0064423-002 |  |
| HSALNG0075408 |  |
| HSALNG0082763 |  |
| HSALNG0111470 |  |
| LOC105374186 | Uncharacterized LOC105374186 |
| LOC124905121 | Uncharacterized LOC124905121 |
| MK279975 |  |
| MK280073-543 |  |
| NONHSAG029010.2 |  |
| RF00017-328 |  |
| lnc-BOLL-6 |  |
| piR-36746-006 |  |
| piR-43107-277 |  |
| piR-46847-250 |  |
| HSALNG0060213 |  |
| HSALNG0150175 |  |
| LOC100419686 | Zinc Finger Protein 256 Pseudogene |
| lnc-CPNE8-5 |  |
| piR-32214-189 |  |
| piR-37910 |  |
| piR-42076-007 |  |
| piR-46002-536 |  |
| CELF2 | CUGBP Elav-Like Family Member 2 |
| PRSS8 | Serine Protease 8 |
| CCL17 | C-C Motif Chemokine Ligand 17 |
| CCL4 | C-C Motif Chemokine Ligand 4 |
| SLC17A8 | Solute Carrier Family 17 Member 8 |
| CCL13 | C-C Motif Chemokine Ligand 13 |
| PEBP1 | Phosphatidylethanolamine Binding Protein 1 |
| ADRA1A | Adrenoceptor Alpha 1A |
| RGS20 | Regulator Of G Protein Signaling 20 |
| ITGB3 | Integrin Subunit Beta 3 |
| PGR | Progesterone Receptor |
| GRIA2 | Glutamate Ionotropic Receptor AMPA Type Subunit 2 |
| INSR | Insulin Receptor |
| LHCGR | Luteinizing Hormone/Choriogonadotropin Receptor |
| MAOB | Monoamine Oxidase B |
| FBXL17 | F-Box And Leucine Rich Repeat Protein 17 |
| NPM1 | Nucleophosmin 1 |
| RANBP2 | RAN Binding Protein 2 |
| MARS1 | Methionyl-TRNA Synthetase 1 |
| TLK2 | Tousled Like Kinase 2 |
| EFTUD2 | Elongation Factor Tu GTP Binding Domain Containing 2 |
| SCYL2 | SCY1 Like Pseudokinase 2 |
| PRDX2 | Peroxiredoxin 2 |
| NR1I2 | Nuclear Receptor Subfamily 1 Group I Member 2 |
| SLC16A1 | Solute Carrier Family 16 Member 1 |
| YWHAB | Tyrosine 3-Monooxygenase/Tryptophan 5-Monooxygenase Activation Protein Beta |
| MMP2 | Matrix Metallopeptidase 2 |
| EIF4EBP1 | Eukaryotic Translation Initiation Factor 4E Binding Protein 1 |
| KAT8 | Lysine Acetyltransferase 8 |
| USF1 | Upstream Transcription Factor 1 |
| CRX | Cone-Rod Homeobox |
| AR | Androgen Receptor |
| KCNH7 | Potassium Voltage-Gated Channel Subfamily H Member 7 |
| PTS | 6-Pyruvoyltetrahydropterin Synthase |
| PRKN | Parkin RBR E3 Ubiquitin Protein Ligase |
| MT-TK | Mitochondrially Encoded TRNA-Lys (AAA/G) |
| SLC5A2 | Solute Carrier Family 5 Member 2 |
| ENPP1 | Ectonucleotide Pyrophosphatase/Phosphodiesterase 1 |
| ENG | Endoglin |
| CCKBR | Cholecystokinin B Receptor |
| GHSR | Growth Hormone Secretagogue Receptor |
| AKT1 | AKT Serine/Threonine Kinase 1 |
| PLA2G7 | Phospholipase A2 Group VII |
| CETP | Cholesteryl Ester Transfer Protein |
| CHI3L1 | Chitinase 3 Like 1 |
| MIR342 | MicroRNA 342 |
| BRF1 | BRF1 RNA Polymerase III Transcription Initiation Factor Subunit |
| COL1A1 | Collagen Type I Alpha 1 Chain |
| VTRNA1-1 | Vault RNA 1-1 |
| TUBB | Tubulin Beta Class I |
| PRMT1 | Protein Arginine Methyltransferase 1 |
| YWHAQ | Tyrosine 3-Monooxygenase/Tryptophan 5-Monooxygenase Activation Protein Theta |
| DYNC1H1 | Dynein Cytoplasmic 1 Heavy Chain 1 |
| SNRNP200 | Small Nuclear Ribonucleoprotein U5 Subunit 200 |
| HNRNPH1 | Heterogeneous Nuclear Ribonucleoprotein H1 |
| RTN2 | Reticulon 2 |
| PSMC2 | Proteasome 26S Subunit, ATPase 2 |
| NASP | Nuclear Autoantigenic Sperm Protein |
| MAFK | MAF BZIP Transcription Factor K |
| PDPK1 | 3-Phosphoinositide Dependent Protein Kinase 1 |
| CYP19A1 | Cytochrome P450 Family 19 Subfamily A Member 1 |
| ANXA1 | Annexin A1 |
| ADRA2B | Adrenoceptor Alpha 2B |
| APRT | Adenine Phosphoribosyltransferase |
| MEN1 | Menin 1 |
| DST | Dystonin |
| ATP5F1D | ATP Synthase F1 Subunit Delta |
| CGB5 | Chorionic Gonadotropin Subunit Beta 5 |
| ZNF423 | Zinc Finger Protein 423 |
| EIF4G1 | Eukaryotic Translation Initiation Factor 4 Gamma 1 |
| ADRA2A | Adrenoceptor Alpha 2A |
| HCRTR1 | Hypocretin Receptor 1 |
| MBL2 | Mannose Binding Lectin 2 |
| ABCC2 | ATP Binding Cassette Subfamily C Member 2 |
| YWHAH | Tyrosine 3-Monooxygenase/Tryptophan 5-Monooxygenase Activation Protein Eta |
| BRCA2 | BRCA2 DNA Repair Associated |
| NMNAT1 | Nicotinamide Nucleotide Adenylyltransferase 1 |
| BLNK | B Cell Linker |
| PIAS1 | Protein Inhibitor Of Activated STAT 1 |
| SFN | Stratifin |
| BTC | Betacellulin |
| INPP5D | Inositol Polyphosphate-5-Phosphatase D |
| BEST1 | Bestrophin 1 |
| CLCN6 | Chloride Voltage-Gated Channel 6 |
| IRF4 | Interferon Regulatory Factor 4 |
| LOXL3 | Lysyl Oxidase Like 3 |
| PDE1B | Phosphodiesterase 1B |
| STX4 | Syntaxin 4 |
| PEA15 | Proliferation And Apoptosis Adaptor Protein 15 |
| SLC8A2 | Solute Carrier Family 8 Member A2 |
| H1-5 | H1.5 Linker Histone, Cluster Member |
| KMT5B | Lysine Methyltransferase 5B |
| ADGRB3 | Adhesion G Protein-Coupled Receptor B3 |
| BRWD1 | Bromodomain And WD Repeat Domain Containing 1 |
| DMAP1 | DNA Methyltransferase 1 Associated Protein 1 |
| DOK5 | Docking Protein 5 |
| SCAMP2 | Secretory Carrier Membrane Protein 2 |
| MTREX | Mtr4 Exosome RNA Helicase |
| SYCP2 | Synaptonemal Complex Protein 2 |
| DUSP8 | Dual Specificity Phosphatase 8 |
| FOXL1 | Forkhead Box L1 |
| KCNV1 | Potassium Voltage-Gated Channel Modifier Subfamily V Member 1 |
| RNPC3 | RNA Binding Region (RNP1, RRM) Containing 3 |
| CFAP65 | Cilia And Flagella Associated Protein 65 |
| FKBP11 | FKBP Prolyl Isomerase 11 |
| SLC39A12 | Solute Carrier Family 39 Member 12 |
| URGCP | Upregulator Of Cell Proliferation |
| ZNF75D | Zinc Finger Protein 75D |
| DENND2D | DENN Domain Containing 2D |
| RNF103 | Ring Finger Protein 103 |
| MAP7D1 | MAP7 Domain Containing 1 |
| OR2W1 | Olfactory Receptor Family 2 Subfamily W Member 1 |
| TCTA | T Cell Leukemia Translocation Altered |
| GLMP | Glycosylated Lysosomal Membrane Protein |
| ZNF211 | Zinc Finger Protein 211 |
| PRR14L | Proline Rich 14 Like |
| DIO2 | Iodothyronine Deiodinase 2 |
| NFE2 | Nuclear Factor, Erythroid 2 |
| RCVRN | Recoverin |
| HAMP | Hepcidin Antimicrobial Peptide |
| HNMT | Histamine N-Methyltransferase |
| UTS2 | Urotensin 2 |
| JUN | Jun Proto-Oncogene, AP-1 Transcription Factor Subunit |
| PEPD | Peptidase D |
| SCP2 | Sterol Carrier Protein 2 |
| DISC1 | DISC1 Scaffold Protein |
| THBS1 | Thrombospondin 1 |
| TCN1 | Transcobalamin 1 |
| ECE1 | Endothelin Converting Enzyme 1 |
| GNAI2 | G Protein Subunit Alpha I2 |
| BMP6 | Bone Morphogenetic Protein 6 |
| SDHD | Succinate Dehydrogenase Complex Subunit D |
| BAZ1B | Bromodomain Adjacent To Zinc Finger Domain 1B |
| POU1F1 | POU Class 1 Homeobox 1 |
| IL15 | Interleukin 15 |
| NTRK2 | Neurotrophic Receptor Tyrosine Kinase 2 |
| MTTP | Microsomal Triglyceride Transfer Protein |
| ATG7 | Autophagy Related 7 |
| CLDN4 | Claudin 4 |
| HOMER1 | Homer Scaffold Protein 1 |
| PAX4 | Paired Box 4 |
| ATF5 | Activating Transcription Factor 5 |
| NFILZ | NFIL3 Like Basic Leucine Zipper |
| SLC1A3 | Solute Carrier Family 1 Member 3 |
| EGFR | Epidermal Growth Factor Receptor |
| TRPV5 | Transient Receptor Potential Cation Channel Subfamily V Member 5 |
| THPO | Thrombopoietin |
| SLC43A2 | Solute Carrier Family 43 Member 2 |
| SYVN1 | Synoviolin 1 |
| BRINP1 | BMP/Retinoic Acid Inducible Neural Specific 1 |
| NPHP3-ACAD11 | NPHP3-ACAD11 Readthrough (NMD Candidate) |
| SLC7A2 | Solute Carrier Family 7 Member 2 |
| HSPA4 | Heat Shock Protein Family A (Hsp70) Member 4 |
| DUSP1 | Dual Specificity Phosphatase 1 |
| GIPR | Gastric Inhibitory Polypeptide Receptor |
| SNORD116-1 | Small Nucleolar RNA, C/D Box 116-1 |
| ABCC4 | ATP Binding Cassette Subfamily C Member 4 |
| PDE5A | Phosphodiesterase 5A |
| CAMK2A | Calcium/Calmodulin Dependent Protein Kinase II Alpha |
| GRIN1 | Glutamate Ionotropic Receptor NMDA Type Subunit 1 |
| VGF | VGF Nerve Growth Factor Inducible |
| YY1 | YY1 Transcription Factor |
| MADD | MAP Kinase Activating Death Domain |
| LINC01554 | Long Intergenic Non-Protein Coding RNA 1554 |
| ATP7B | ATPase Copper Transporting Beta |
| MMP8 | Matrix Metallopeptidase 8 |
| IL1R1 | Interleukin 1 Receptor Type 1 |
| LIPC | Lipase C, Hepatic Type |
| GPD1 | Glycerol-3-Phosphate Dehydrogenase 1 |
| TRIP12 | Thyroid Hormone Receptor Interactor 12 |
| MIR98 | MicroRNA 98 |
| GRM5 | Glutamate Metabotropic Receptor 5 |
| PTGER3 | Prostaglandin E Receptor 3 |
| QDPR | Quinoid Dihydropteridine Reductase |
| UCP1 | Uncoupling Protein 1 |
| GDNF | Glial Cell Derived Neurotrophic Factor |
| SULT1A1 | Sulfotransferase Family 1A Member 1 |
| WEE1 | WEE1 G2 Checkpoint Kinase |
| APLNR | Apelin Receptor |
| NMB | Neuromedin B |
| CYP11A1 | Cytochrome P450 Family 11 Subfamily A Member 1 |
| BCL2A1 | BCL2 Related Protein A1 |
| MGP | Matrix Gla Protein |
| GHRHR | Growth Hormone Releasing Hormone Receptor |
| C9orf72 | C9orf72-SMCR8 Complex Subunit |
| MSMB | Microseminoprotein Beta |
| PRND | Prion Like Protein Doppel |
| TXNDC9 | Thioredoxin Domain Containing 9 |
| ERI3 | ERI1 Exoribonuclease Family Member 3 |
| SPRN | Shadow Of Prion Protein |
| MIRLET7I | MicroRNA Let-7i |
| PANK2 | Pantothenate Kinase 2 |
| NUP98 | Nucleoporin 98 And 96 Precursor |
| RAE1 | Ribonucleic Acid Export 1 |
| GPRASP1 | G Protein-Coupled Receptor Associated Sorting Protein 1 |
| CTCF | CCCTC-Binding Factor |
| CTSD | Cathepsin D |
| PKLR | Pyruvate Kinase L/R |
| CDH1 | Cadherin 1 |
| ANGPTL2 | Angiopoietin Like 2 |
| CARMN | Cardiac Mesoderm Enhancer-Associated Non-Coding RNA |
| LBX1 | Ladybird Homeobox 1 |
| GRM8 | Glutamate Metabotropic Receptor 8 |
| PTH2R | Parathyroid Hormone 2 Receptor |
| SULT2A1 | Sulfotransferase Family 2A Member 1 |
| OGFR | Opioid Growth Factor Receptor |
| PPP4C | Protein Phosphatase 4 Catalytic Subunit |
| SLC17A5 | Solute Carrier Family 17 Member 5 |
| VTN | Vitronectin |
| BECN1 | Beclin 1 |
| NRXN1 | Neurexin 1 |
| ENOX2 | Ecto-NOX Disulfide-Thiol Exchanger 2 |
| MIRLET7F1 | MicroRNA Let-7f-1 |
| APOC4-APOC2 | APOC4-APOC2 Readthrough (NMD Candidate) |
| CDKN1B | Cyclin Dependent Kinase Inhibitor 1B |
| SUGP1 | SURP And G-Patch Domain Containing 1 |
| GCKR | Glucokinase Regulator |
| TACR1 | Tachykinin Receptor 1 |
| GNB4 | G Protein Subunit Beta 4 |
| MTAP | Methylthioadenosine Phosphorylase |
| MT2A | Metallothionein 2A |
| SLC6A1 | Solute Carrier Family 6 Member 1 |
| SELL | Selectin L |
| CAVIN2 | Caveolae Associated Protein 2 |
| MDS2 | Myelodysplastic Syndrome 2 Translocation Associated |
| RAB4B-EGLN2 | RAB4B-EGLN2 Readthrough (NMD Candidate) |
| OPRL1 | Opioid Related Nociceptin Receptor 1 |
| ADRB3 | Adrenoceptor Beta 3 |
| CREB3L1 | CAMP Responsive Element Binding Protein 3 Like 1 |
| TTF2 | Transcription Termination Factor 2 |
| DEFB1 | Defensin Beta 1 |
| EIF3B | Eukaryotic Translation Initiation Factor 3 Subunit B |
| TOR2A | Torsin Family 2 Member A |
| MSH6 | MutS Homolog 6 |
| NPFF | Neuropeptide FF-Amide Peptide Precursor |
| LOC106099062 | HBB Recombination Region |
| LOC107133510 | Origin Of Replication At HBB |
| ITGAV | Integrin Subunit Alpha V |
| ITGA2 | Integrin Subunit Alpha 2 |
| CD79A | CD79a Molecule |
| P2RX4 | Purinergic Receptor P2X 4 |
| PTGFR | Prostaglandin F Receptor |
| UMOD | Uromodulin |
| P2RX6 | Purinergic Receptor P2X 6 |
| RARRES2 | Retinoic Acid Receptor Responder 2 |
| GUCA2B | Guanylate Cyclase Activator 2B |
| LSM12 | LSM12 Homolog |
| RLN3 | Relaxin 3 |
| ALDH2 | Aldehyde Dehydrogenase 2 Family Member |
| NPAS3 | Neuronal PAS Domain Protein 3 |
| TMEM11 | Transmembrane Protein 11 |
| MIR26B | MicroRNA 26b |
| MIR203A | MicroRNA 203a |
| MIR485 | MicroRNA 485 |
| GABBR2 | Gamma-Aminobutyric Acid Type B Receptor Subunit 2 |
| SNRPN | Small Nuclear Ribonucleoprotein Polypeptide N |
| F5 | Coagulation Factor V |
| GRN | Granulin Precursor |
| SI | Sucrase-Isomaltase |
| ADAMTS7 | ADAM Metallopeptidase With Thrombospondin Type 1 Motif 7 |
| SSPN | Sarcospan |
| PSMD12 | Proteasome 26S Subunit, Non-ATPase 12 |
| SNORD74 | Small Nucleolar RNA, C/D Box 74 |
| PAH | Phenylalanine Hydroxylase |
| CD9 | CD9 Molecule |
| LMOD1 | Leiomodin 1 |
| FKBP5 | FKBP Prolyl Isomerase 5 |
| CCR5 | C-C Motif Chemokine Receptor 5 |
| ARX | Aristaless Related Homeobox |
| FADS1 | Fatty Acid Desaturase 1 |
| MIR19B1 | MicroRNA 19b-1 |
| GH-LCR | Growth Hormone Locus Control Region |
| USP46 | Ubiquitin Specific Peptidase 46 |
| FN1 | Fibronectin 1 |
| MYOC | Myocilin |
| MYLK | Myosin Light Chain Kinase |
| KCNJ1 | Potassium Inwardly Rectifying Channel Subfamily J Member 1 |
| HCFC1 | Host Cell Factor C1 |
| MRTFA | Myocardin Related Transcription Factor A |
| PKP1 | Plakophilin 1 |
| SEC13 | SEC13 Homolog, Nuclear Pore And COPII Coat Complex Component |
| VPS4A | Vacuolar Protein Sorting 4 Homolog A |
| CRYGD | Crystallin Gamma D |
| KCNG2 | Potassium Voltage-Gated Channel Modifier Subfamily G Member 2 |
| MYL5 | Myosin Light Chain 5 |
| ODC1 | Ornithine Decarboxylase 1 |
| CDC25A | Cell Division Cycle 25A |
| NR4A1 | Nuclear Receptor Subfamily 4 Group A Member 1 |
| FOSB | FosB Proto-Oncogene, AP-1 Transcription Factor Subunit |
| JUNB | JunB Proto-Oncogene, AP-1 Transcription Factor Subunit |
| MIR122 | MicroRNA 122 |
| PTX3 | Pentraxin 3 |
| DIO3 | Iodothyronine Deiodinase 3 |
| MIR1291 | MicroRNA 1291 |
| DCAF8 | DDB1 And CUL4 Associated Factor 8 |
| MIR106B | MicroRNA 106b |
| PHB2 | Prohibitin 2 |
| CXCR4 | C-X-C Motif Chemokine Receptor 4 |
| MIR26A1 | MicroRNA 26a-1 |
| RBX1 | Ring-Box 1 |
| GRIA3 | Glutamate Ionotropic Receptor AMPA Type Subunit 3 |
| TAF1 | TATA-Box Binding Protein Associated Factor 1 |
| CHD8 | Chromodomain Helicase DNA Binding Protein 8 |
| ATP10A | ATPase Phospholipid Transporting 10A (Putative) |
| TELO2 | Telomere Maintenance 2 |
| KIF15 | Kinesin Family Member 15 |
| QRFP | Pyroglutamylated RFamide Peptide |
| CASP8 | Caspase 8 |
| NFKBIA | NFKB Inhibitor Alpha |
| FAS | Fas Cell Surface Death Receptor |
| IRS2 | Insulin Receptor Substrate 2 |
| RAPGEF3 | Rap Guanine Nucleotide Exchange Factor 3 |
| G6PC1 | Glucose-6-Phosphatase Catalytic Subunit 1 |
| OPRK1 | Opioid Receptor Kappa 1 |
| NAT2 | N-Acetyltransferase 2 |
| CYP20A1 | Cytochrome P450 Family 20 Subfamily A Member 1 |
| FHL5 | Four And A Half LIM Domains 5 |
| JPX | JPX Transcript, XIST Activator |
| MIR190B | MicroRNA 190b |
| MIR379 | MicroRNA 379 |
| LINC01191 | Long Intergenic Non-Protein Coding RNA 1191 |
| MT-RNR1 | Mitochondrially Encoded 12S RRNA |
| SNORD25 | Small Nucleolar RNA, C/D Box 25 |
| LOC109279247 | FGF21/FUT1 Promoter Region |
| STIN2-VNTR | Serotonin Transporter Intronic VNTR Enhancer |
| FGF2 | Fibroblast Growth Factor 2 |
| EXOSC5 | Exosome Component 5 |
| FXR1 | FMR1 Autosomal Homolog 1 |
| FTL | Ferritin Light Chain |
| C19orf12 | Chromosome 19 Open Reading Frame 12 |
| RPS6KA5 | Ribosomal Protein S6 Kinase A5 |
| DAOA-AS1 | DAOA Antisense RNA 1 |
| CHRNA2 | Cholinergic Receptor Nicotinic Alpha 2 Subunit |
| SLC6A9 | Solute Carrier Family 6 Member 9 |
| SPP1 | Secreted Phosphoprotein 1 |
| ANG | Angiogenin |
| PIGA | Phosphatidylinositol Glycan Anchor Biosynthesis Class A |
| IGF2R | Insulin Like Growth Factor 2 Receptor |
| CANX | Calnexin |
| AKAP13 | A-Kinase Anchoring Protein 13 |
| CRELD1 | Cysteine Rich With EGF Like Domains 1 |
| MIR144 | MicroRNA 144 |
| TARID | TCF21 Antisense RNA Inducing Promoter Demethylation |
| CREB3L3 | CAMP Responsive Element Binding Protein 3 Like 3 |
| DHFR | Dihydrofolate Reductase |
| HIF1AN | Hypoxia Inducible Factor 1 Subunit Alpha Inhibitor |
| ZBTB20 | Zinc Finger And BTB Domain Containing 20 |
| UBQLN4 | Ubiquilin 4 |
| NEIL1 | Nei Like DNA Glycosylase 1 |
| IL6R | Interleukin 6 Receptor |
| COMP | Cartilage Oligomeric Matrix Protein |
| LTF | Lactotransferrin |
| CCR2 | C-C Motif Chemokine Receptor 2 |
| CTNS | Cystinosin, Lysosomal Cystine Transporter |
| DLK1 | Delta Like Non-Canonical Notch Ligand 1 |
| GABRA6 | Gamma-Aminobutyric Acid Type A Receptor Subunit Alpha6 |
| HRH2 | Histamine Receptor H2 |
| SRD5A1 | Steroid 5 Alpha-Reductase 1 |
| INHBB | Inhibin Subunit Beta B |
| SCGB1A1 | Secretoglobin Family 1A Member 1 |
| LCN1 | Lipocalin 1 |
| PMCH | Pro-Melanin Concentrating Hormone |
| DLEU2 | Deleted In Lymphocytic Leukemia 2 |
| TNFRSF11A | TNF Receptor Superfamily Member 11a |
| CDC73 | Cell Division Cycle 73 |
| FOXF1 | Forkhead Box F1 |
| BOLA3 | BolA Family Member 3 |
| NQO2 | N-Ribosyldihydronicotinamide:Quinone Dehydrogenase 2 |
| GAPVD1 | GTPase Activating Protein And VPS9 Domains 1 |
| SLC24A3 | Solute Carrier Family 24 Member 3 |
| RPS6KA1 | Ribosomal Protein S6 Kinase A1 |
| SLC1A2 | Solute Carrier Family 1 Member 2 |
| SKP2 | S-Phase Kinase Associated Protein 2 |
| FBXO31 | F-Box Protein 31 |
| TUG1 | Taurine Up-Regulated 1 |
| EGF | Epidermal Growth Factor |
| CYP17A1 | Cytochrome P450 Family 17 Subfamily A Member 1 |
| SLC6A2 | Solute Carrier Family 6 Member 2 |
| AMBP | Alpha-1-Microglobulin/Bikunin Precursor |
| ADCY5 | Adenylate Cyclase 5 |
| ANKK1 | Ankyrin Repeat And Kinase Domain Containing 1 |
| SLC32A1 | Solute Carrier Family 32 Member 1 |
| GALNS | Galactosamine (N-Acetyl)-6-Sulfatase |
| MERTK | MER Proto-Oncogene, Tyrosine Kinase |
| DUSP6 | Dual Specificity Phosphatase 6 |
| CD81 | CD81 Molecule |
| DDIT3 | DNA Damage Inducible Transcript 3 |
| EHMT2 | Euchromatic Histone Lysine Methyltransferase 2 |
| MASP2 | MBL Associated Serine Protease 2 |
| PRDX6 | Peroxiredoxin 6 |
| CTSS | Cathepsin S |
| SAG | S-Antigen Visual Arrestin |
| ST3GAL3 | ST3 Beta-Galactoside Alpha-2,3-Sialyltransferase 3 |
| ATF3 | Activating Transcription Factor 3 |
| CYP1A2 | Cytochrome P450 Family 1 Subfamily A Member 2 |
| NFE2L1 | NFE2 Like BZIP Transcription Factor 1 |
| CEBPE | CCAAT Enhancer Binding Protein Epsilon |
| CHRNA9 | Cholinergic Receptor Nicotinic Alpha 9 Subunit |
| EEA1 | Early Endosome Antigen 1 |
| FABP7 | Fatty Acid Binding Protein 7 |
| HTR2B | 5-Hydroxytryptamine Receptor 2B |
| TRNT1 | TRNA Nucleotidyl Transferase 1 |
| COP1 | COP1 E3 Ubiquitin Ligase |
| RGR | Retinal G Protein Coupled Receptor |
| ATF7 | Activating Transcription Factor 7 |
| DUSP4 | Dual Specificity Phosphatase 4 |
| IAPP | Islet Amyloid Polypeptide |
| INHBE | Inhibin Subunit Beta E |
| PRSS2 | Serine Protease 2 |
| SAA4 | Serum Amyloid A4, Constitutive |
| BATF3 | Basic Leucine Zipper ATF-Like Transcription Factor 3 |
| CEBPG | CCAAT Enhancer Binding Protein Gamma |
| INHBC | Inhibin Subunit Beta C |
| MRGPRX1 | MAS Related GPR Family Member X1 |
| ANGPTL8 | Angiopoietin Like 8 |
| MIR541 | MicroRNA 541 |
| SNORA4 | Small Nucleolar RNA, H/ACA Box 4 |
| SNORA8 | Small Nucleolar RNA, H/ACA Box 8 |
| RAD51-AS1 | RAD51 Antisense RNA 1 |
| SNORA13 | Small Nucleolar RNA, H/ACA Box 13 |
| LINP1 | LncRNA In Non-Homologous End Joining Pathway 1 |
| MDC1-AS1 | MDC1 Antisense RNA 1 |
| MIR511 | MicroRNA 511 |
| PURPL | P53 Upregulated Regulator Of P53 Levels |
| LNCTAM34A | Long Non Coding Transcriptional Activator Of MiR34a |
| DINOL | Damage Induced Long Noncoding RNA |
| IL2RB | Interleukin 2 Receptor Subunit Beta |
| PTGS2 | Prostaglandin-Endoperoxide Synthase 2 |
| NPR1 | Natriuretic Peptide Receptor 1 |
| AKR1B1 | Aldo-Keto Reductase Family 1 Member B |
| COL18A1 | Collagen Type XVIII Alpha 1 Chain |
| TGFBI | Transforming Growth Factor Beta Induced |
| IFNB1 | Interferon Beta 1 |
| AADAC | Arylacetamide Deacetylase |
| PTMA | Prothymosin Alpha |
| ITLN1 | Intelectin 1 |
| GALP | Galanin Like Peptide |
| ENSG00000228741 | Spermatogenesis Associated 13 |
| PSMB1 | Proteasome 20S Subunit Beta 1 |
| UBE2O | Ubiquitin Conjugating Enzyme E2 O |
| UCHL5 | Ubiquitin C-Terminal Hydrolase L5 |
| TBL3 | Transducin Beta Like 3 |
| COPS6 | COP9 Signalosome Subunit 6 |
| ZNF207 | Zinc Finger Protein 207 |
| USP38 | Ubiquitin Specific Peptidase 38 |
| ZNF174 | Zinc Finger Protein 174 |
| ZNF44 | Zinc Finger Protein 44 |
| CX3CR1 | C-X3-C Motif Chemokine Receptor 1 |
| MIR30A | MicroRNA 30a |
| SUZ12 | SUZ12 Polycomb Repressive Complex 2 Subunit |
| PTK2 | Protein Tyrosine Kinase 2 |
| ACSF3 | Acyl-CoA Synthetase Family Member 3 |
| SLC22A8 | Solute Carrier Family 22 Member 8 |
| WNK1 | WNK Lysine Deficient Protein Kinase 1 |
| HSD17B4 | Hydroxysteroid 17-Beta Dehydrogenase 4 |
| SLC12A4 | Solute Carrier Family 12 Member 4 |
| IPO4 | Importin 4 |
| MMP1 | Matrix Metallopeptidase 1 |
| CHRM3 | Cholinergic Receptor Muscarinic 3 |
| HRH3 | Histamine Receptor H3 |
| NPHP1 | Nephrocystin 1 |
| TNFSF11 | TNF Superfamily Member 11 |
| CALB2 | Calbindin 2 |
| DUSP19 | Dual Specificity Phosphatase 19 |
| SYP | Synaptophysin |
| NCOR2 | Nuclear Receptor Corepressor 2 |
| SNORD102 | Small Nucleolar RNA, C/D Box 102 |
| CAMK2D | Calcium/Calmodulin Dependent Protein Kinase II Delta |
| GNRHR | Gonadotropin Releasing Hormone Receptor |
| HCN2 | Hyperpolarization Activated Cyclic Nucleotide Gated Potassium And Sodium Channel 2 |
| ASCL1 | Achaete-Scute Family BHLH Transcription Factor 1 |
| GABRG2 | Gamma-Aminobutyric Acid Type A Receptor Subunit Gamma2 |
| MEG3 | Maternally Expressed 3 |
| RNASEH2C | Ribonuclease H2 Subunit C |
| GLIS3 | GLIS Family Zinc Finger 3 |
| MIR135B | MicroRNA 135b |
| ERBB2 | Erb-B2 Receptor Tyrosine Kinase 2 |
| ELANE | Elastase, Neutrophil Expressed |
| ADK | Adenosine Kinase |
| AHCY | Adenosylhomocysteinase |
| TBXA2R | Thromboxane A2 Receptor |
| PRKCZ | Protein Kinase C Zeta |
| AFP | Alpha Fetoprotein |
| BACE1 | Beta-Secretase 1 |
| NR1H4 | Nuclear Receptor Subfamily 1 Group H Member 4 |
| PLK4 | Polo Like Kinase 4 |
| AQP5 | Aquaporin 5 |
| AVPR2 | Arginine Vasopressin Receptor 2 |
| BIRC5 | Baculoviral IAP Repeat Containing 5 |
| GLDC | Glycine Decarboxylase |
| LPAR1 | Lysophosphatidic Acid Receptor 1 |
| PAFAH1B1 | Platelet Activating Factor Acetylhydrolase 1b Regulatory Subunit 1 |
| SPRY2 | Sprouty RTK Signaling Antagonist 2 |
| TGFA | Transforming Growth Factor Alpha |
| ACACB | Acetyl-CoA Carboxylase Beta |
| MKI67 | Marker Of Proliferation Ki-67 |
| NR5A2 | Nuclear Receptor Subfamily 5 Group A Member 2 |
| RBP4 | Retinol Binding Protein 4 |
| ADD1 | Adducin 1 |
| EXOSC3 | Exosome Component 3 |
| HLA-G | Major Histocompatibility Complex, Class I, G |
| IL18R1 | Interleukin 18 Receptor 1 |
| SLC16A2 | Solute Carrier Family 16 Member 2 |
| SLC6A19 | Solute Carrier Family 6 Member 19 |
| TBXT | T-Box Transcription Factor T |
| ADH7 | Alcohol Dehydrogenase 7 (Class IV), Mu Or Sigma Polypeptide |
| CXCL1 | C-X-C Motif Chemokine Ligand 1 |
| DDAH1 | Dimethylarginine Dimethylaminohydrolase 1 |
| FANCM | FA Complementation Group M |
| FBP2 | Fructose-Bisphosphatase 2 |
| PDK4 | Pyruvate Dehydrogenase Kinase 4 |
| S100A6 | S100 Calcium Binding Protein A6 |
| AOC1 | Amine Oxidase Copper Containing 1 |
| ARR3 | Arrestin 3 |
| DIO1 | Iodothyronine Deiodinase 1 |
| EXOSC2 | Exosome Component 2 |
| HPGDS | Hematopoietic Prostaglandin D Synthase |
| IL11 | Interleukin 11 |
| ME1 | Malic Enzyme 1 |
| CMKLR1 | Chemerin Chemokine-Like Receptor 1 |
| CXCL5 | C-X-C Motif Chemokine Ligand 5 |
| MAP1LC3A | Microtubule Associated Protein 1 Light Chain 3 Alpha |
| MIP | Major Intrinsic Protein Of Lens Fiber |
| OSBP | Oxysterol Binding Protein |
| PTGER1 | Prostaglandin E Receptor 1 |
| S100A1 | S100 Calcium Binding Protein A1 |
| SLC2A6 | Solute Carrier Family 2 Member 6 |
| TNRC6A | Trinucleotide Repeat Containing Adaptor 6A |
| GPBAR1 | G Protein-Coupled Bile Acid Receptor 1 |
| SMN2 | Survival Of Motor Neuron 2, Centromeric |
| TPCN2 | Two Pore Segment Channel 2 |
| TPCN1 | Two Pore Segment Channel 1 |
| CSN2 | Casein Beta |
| MREG | Melanoregulin |
| RABEP2 | Rabaptin, RAB GTPase Binding Effector Protein 2 |
| GNRH2 | Gonadotropin Releasing Hormone 2 |
| KIAA1549L | KIAA1549 Like |
| AMY1C | Amylase Alpha 1C |
| AMY1B | Amylase Alpha 1B |
| NBPF14 | NBPF Member 14 |
| MIRLET7D | MicroRNA Let-7d |
| MIRLET7A2 | MicroRNA Let-7a-2 |
| MIR16-2 | MicroRNA 16-2 |
| MIRLET7G | MicroRNA Let-7g |
| LINC00458 | Long Intergenic Non-Protein Coding RNA 458 |
| SNORD50A | Small Nucleolar RNA, C/D Box 50A |
| SNORD44 | Small Nucleolar RNA, C/D Box 44 |
| LOC106799833 | CYP11B1 Recombination Region |
| LOC110596866 | CYP7A1 5' Regulatory Region |
| RIPOR2 | RHO Family Interacting Cell Polarization Regulator 2 |
| ADORA2B | Adenosine A2b Receptor |
| PTPRS | Protein Tyrosine Phosphatase Receptor Type S |
| TXK | TXK Tyrosine Kinase |
| KMT2D | Lysine Methyltransferase 2D |
| ENHO | Energy Homeostasis Associated |
| ROCR | Regulator Of Chondrogenesis RNA |
| SP1 | Sp1 Transcription Factor |
| NMBR | Neuromedin B Receptor |
| STXBP1 | Syntaxin Binding Protein 1 |
| CUL3 | Cullin 3 |
| PAX6 | Paired Box 6 |
| RAB27A | RAB27A, Member RAS Oncogene Family |
| FOXA2 | Forkhead Box A2 |
| RNF217-AS1 | RNF217 Antisense RNA 1 (Head To Head) |
| CCL11 | C-C Motif Chemokine Ligand 11 |
| CCL5 | C-C Motif Chemokine Ligand 5 |
| CLSPN | Claspin |
| FGFR2 | Fibroblast Growth Factor Receptor 2 |
| GRIK2 | Glutamate Ionotropic Receptor Kainate Type Subunit 2 |
| PI4KA | Phosphatidylinositol 4-Kinase Alpha |
| TRPV4 | Transient Receptor Potential Cation Channel Subfamily V Member 4 |
| BRD4 | Bromodomain Containing 4 |
| SETD2 | SET Domain Containing 2, Histone Lysine Methyltransferase |
| GNAI3 | G Protein Subunit Alpha I3 |
| OPA1 | OPA1 Mitochondrial Dynamin Like GTPase |
| VAPB | VAMP Associated Protein B And C |
| ATXN2 | Ataxin 2 |
| UBQLN2 | Ubiquilin 2 |
| VPS35 | VPS35 Retromer Complex Component |
| TINF2 | TERF1 Interacting Nuclear Factor 2 |
| TRIP13 | Thyroid Hormone Receptor Interactor 13 |
| COX11 | Cytochrome C Oxidase Copper Chaperone COX11 |
| MYO9A | Myosin IXA |
| IRF2BP2 | Interferon Regulatory Factor 2 Binding Protein 2 |
| PPP1R21 | Protein Phosphatase 1 Regulatory Subunit 21 |
| CD47 | CD47 Molecule |
| CDH13 | Cadherin 13 |
| GAD2 | Glutamate Decarboxylase 2 |
| AVPR1B | Arginine Vasopressin Receptor 1B |
| GRIN2A | Glutamate Ionotropic Receptor NMDA Type Subunit 2A |
| CHD2 | Chromodomain Helicase DNA Binding Protein 2 |
| NPY4R | Neuropeptide Y Receptor Y4 |
| CORT | Cortistatin |
| PDE2A | Phosphodiesterase 2A |
| EPOR | Erythropoietin Receptor |
| DRD5 | Dopamine Receptor D5 |
| SENP1 | SUMO Specific Peptidase 1 |
| RAC1 | Rac Family Small GTPase 1 |
| SLC30A1 | Solute Carrier Family 30 Member 1 |
| TAC3 | Tachykinin Precursor 3 |
| CHML | CHM Like Rab Escort Protein |
| HNF1A-AS1 | HNF1A Antisense RNA 1 |
| SNORD24 | Small Nucleolar RNA, C/D Box 24 |
| TPH2 | Tryptophan Hydroxylase 2 |
| NFATC2 | Nuclear Factor Of Activated T Cells 2 |
| ALG1 | ALG1 Chitobiosyldiphosphodolichol Beta-Mannosyltransferase |
| MIR149 | MicroRNA 149 |
| CTBP2 | C-Terminal Binding Protein 2 |
| NALF1 | NALCN Channel Auxiliary Factor 1 |
