# Supplementary material for: A diagnostic model for Parkinson’s disease based on circadian rhythm-related genes
Source: J Transl Med. 2024 Jul 8;22:635. doi: 10.1186/s12967-024-05424-z (PMC11229228; doi:10.1186/s12967-024-05424-z)
Supplement: Supplementary file 2 — Supplementary Material 2 [file 12967_2024_5424_MOESM2_ESM.docx]

| **Supplementary Table 2. Primer sequences (5'->3').** | | |
| --- | --- | --- |
| **Gene** | **Forward primer** | **Reverse primer** |
| AGTR1 | ATTTAGCACTGGCTGACTTATGC | CAGCGGTATTCCATAGCTGTG |
| XPA | AGG CGC TCT CAC TCA GAA AG | CTTTCTGAGTGAGAGCGCCT |
| CALR | CCTGCCGTCTACTTCAAGGAG | GAACTTGCCGGAACTGAGAAC |
| RBM14 | ACTTGGAAGATTTTCGTGGGC | CGGAACCATACCCTTGGTGG |
